# Supplementary material for: Specialised and persistent raw material procurement by humans in the Middle Pleistocene
Source: Nat Commun. 2026 Apr 7;17:2702. doi: 10.1038/s41467-026-70783-8 (PMC13057256; doi:10.1038/s41467-026-70783-8)
Supplement: Supplementary file 1 — Supplementary Information [file 41467_2026_70783_MOESM1_ESM.pdf]

Specialised and persistent raw material procurement by humans in the Middle Pleistocene

Will, M., Sommer, C., Möller, G.H.D., Botha, G., Blessing, M.A., Msimanga, L., Mazel, A., Val, A., Venditti, F., Riedesel, S.

Supplementary Information

Supplementary Information Guide

Supplementary Note 1: Geology and geography of the Jojosi Dongas (p. 2-13)

(incl. Supplementary Tables 1-3 and Figures 1-7)

Supplementary Note 2: Luminescence dating (p. 14-18)

(incl. Supplementary Tables 4-6 and Figure 8)

Supplementary Note 3: Excavations and archaeology of the Jojosi Dongas (p. 19-39)

(incl. Supplementary Discussion 1-3 and Supplementary Figures 9-40)

Supplementary Note 4: Lithic assemblages of Jojosi 5, 6, and 7 (p. 40-61)

(incl. Supplementary Tables 7-44 and Figures 41-46)

Supplementary Note 5: Use-wear analysis (p. 62-67)

(incl. Supplementary Table 45-46 and Figures 47-50)

Supplementary Note 6: Zooarchaeological analysis of Jojosi 7 (p. 68-69)

(incl. Supplementary Figure 51)

Supplementary Note 1: Geology and geography of the Jojosi Dongas

1. Overall site description

The Jojosi site lies at the headwaters of the Jojosi River, where a thick channel sandstone within the Vryheid Formation forms a cliff up to 20 m high. Siltstone and shale underlying the sandstone lie in contact with the top of an intrusive dolerite sill, which underlies the intensely gullied landscape over an area of some 30 km that is traversed by the river channel downstream towards the east. The dolerite hills towards the south (1233m) and north (1252m) define the hillslope basin within which the sheetwash colluvial deposits and interbedded palaeosols, correlated with the Masotcheni Formation[1-2], mantle the lower slopes. The present Jojosi channel is incised into bedrock, and the former channel gravels form a terrace deposit elevated some 5m above the present channel base.

2. Methods

2.1 Stratigraphic Description

We documented six sedimentary profiles: two representing the geoscientific reference profile of the site (Jojosi Triple Junction) and four describing the sedimentary succession at archaeological sites (Jojosi 1, Jojosi 5, Jojosi 6, and Jojosi 7). Stratigraphic classification followed the system of Botha[2], which has proven effective for establishing a framework for Late Pleistocene sedimentary deposits of the Masotcheni Formation in KwaZulu-Natal. This approach integrates allostratigraphic and pedostratigraphic techniques to capture both erosional and depositional histories of rock units and the development of pedoderms. In this study, we correlate rock units across all sites and describe the soil characteristics of Jojosi 7 in detail.

2.2 Sediment Analysis

A total of 23 samples for texture and chemical analysis, and 20 samples for mineralogical analysis, were collected. Texture and chemical analyses (full-fraction) were performed at the Soil Science Laboratory, CEDARA College of Agriculture, Hilton, South Africa. Mineralogical composition was determined using semi-quantitative X-ray diffraction (XRD) at the XRD Laboratory, Council for Geoscience, Pretoria, South Africa.

2.3 UAV and GPS mapping

During the 2022–2024 field seasons, we acquired multiple UAV datasets using DJI Phantom 4 Pro and DJI Air 2 platforms to produce annual high-resolution photogrammetric products. We used the Structure-from-Motion software Agisoft Metashape 2.2.1 to create Orthophotos with a spatial resolution of up to 2 cm, Digital Surface Models with spatial resolutions up to 10 cm, and 3D Models. The resulting data were used to create spatial maps for surveying archaeological sites and stratigraphic contacts. Furthermore, we produced orthographic profile sections of gully sidewalls to assist in the mapping and correlation of stratigraphic units over extended distances. Geospatial coordinates of archaeological sites, geoscientific features, and ground control points for stereophotogrammetry were recorded using a Real-Time Kinematic (RTK) differential GPS (ZED-F9P by ArduSimple), receiving correction signals from the Newcastle, Greytown, and Ulundi reference stations via the South African GNSS base station network TrigNet.

3. Profile descriptions

3.1 Triple Junction

Although no archaeological remains were found at the Triple Junction, the locality is considered key to the local stratigraphy and relevant to the general understanding of the landscape. The west-facing wall of a modern channel reveals three earlier generations of gully incision and subsequent infill. Including the basal saprolite, there are four different rock units. Profiles S124 and S134, which are adjacent, help to explain the stratigraphic relationships.

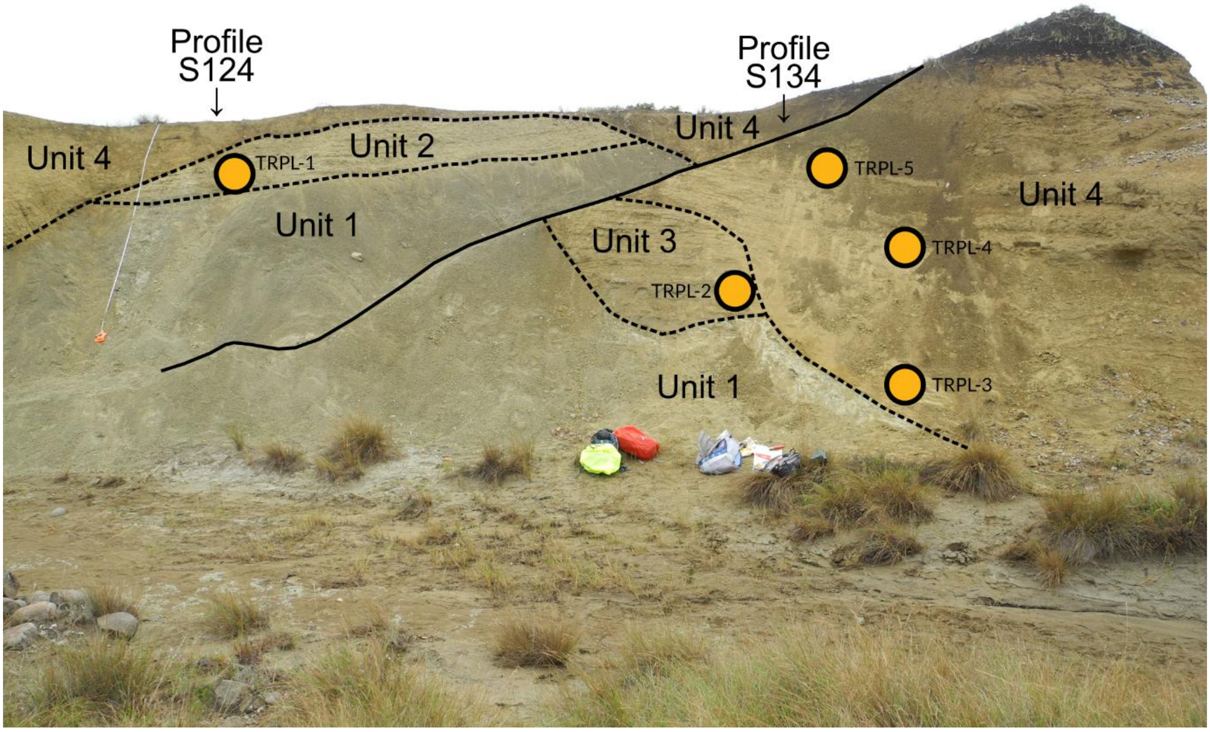

Supplementary Figure 1. Overview of the Jojosi Triple Junction (TRPL) site and luminescence samples (yellow dots) that feature two profiles nearby. Measure tape = 7.5 m.

The profile S124 rises 4.5 m above the modern channel. The basal saprolite (Unit 1) is exposed up to a depth of 1.60 m. The unit is structureless to massive and shows no signs of bedding. The colour is dominated by a greenish grey matrix (GLEY1 5/10GY) and dark slickensides (GLEY1 2.5/N) that represent a manganese film on the surface of aggregates. Greyish green (5GY 5/2) mottles with sharp and diffuse boundaries are found especially in the lower parts of the profile. The matrix consists predominantly of clay and has a subangular, blocky structure. Rare saprock corestones, derived from the original dolerite, can be identified by their yellowish color variation (5Y 7/4), diffuse boundaries, and sandy loam texture. Irregular carbonate nodules account for two percent of the overall matrix but can reach higher densities in association with green mottles (up to five percent). The contact between unit 1 and unit 2 is sharp planar at a depth of 1.6 m and inclined towards the north (following downslope). Unit 2 has a maximal thickness of 0.50 m and is characterized by non-parallel to discontinuous curved bedding. The texture of lenses ranges from loam to loamy sand. The aggregates are angular and blocky, and the colours range from pale yellow (5Y 7/4) to light olive grey (5Y 6/2). The contact between units 2 and 4 at 1.20 m is sharp, planar, and inclined downslope (northerly direction). Unit 4 is 1.20 m thick and has a light olive brown matrix (2.5Y 5/3). A 2–

5 cm-thick layer of clay (5Y 2.5/1) has developed directly above the boundary within Unit 4. The unit is very thinly bedded (<30 mm) to laminated (<10 mm), and the depositional concave bedding surfaces define the infilled channel form. The matrix consists of sandy loam, and the aggregates have an angular to subangular, blocky structure. Dark grey (2.5Y 4/1) clay cutans cover the aggregates.

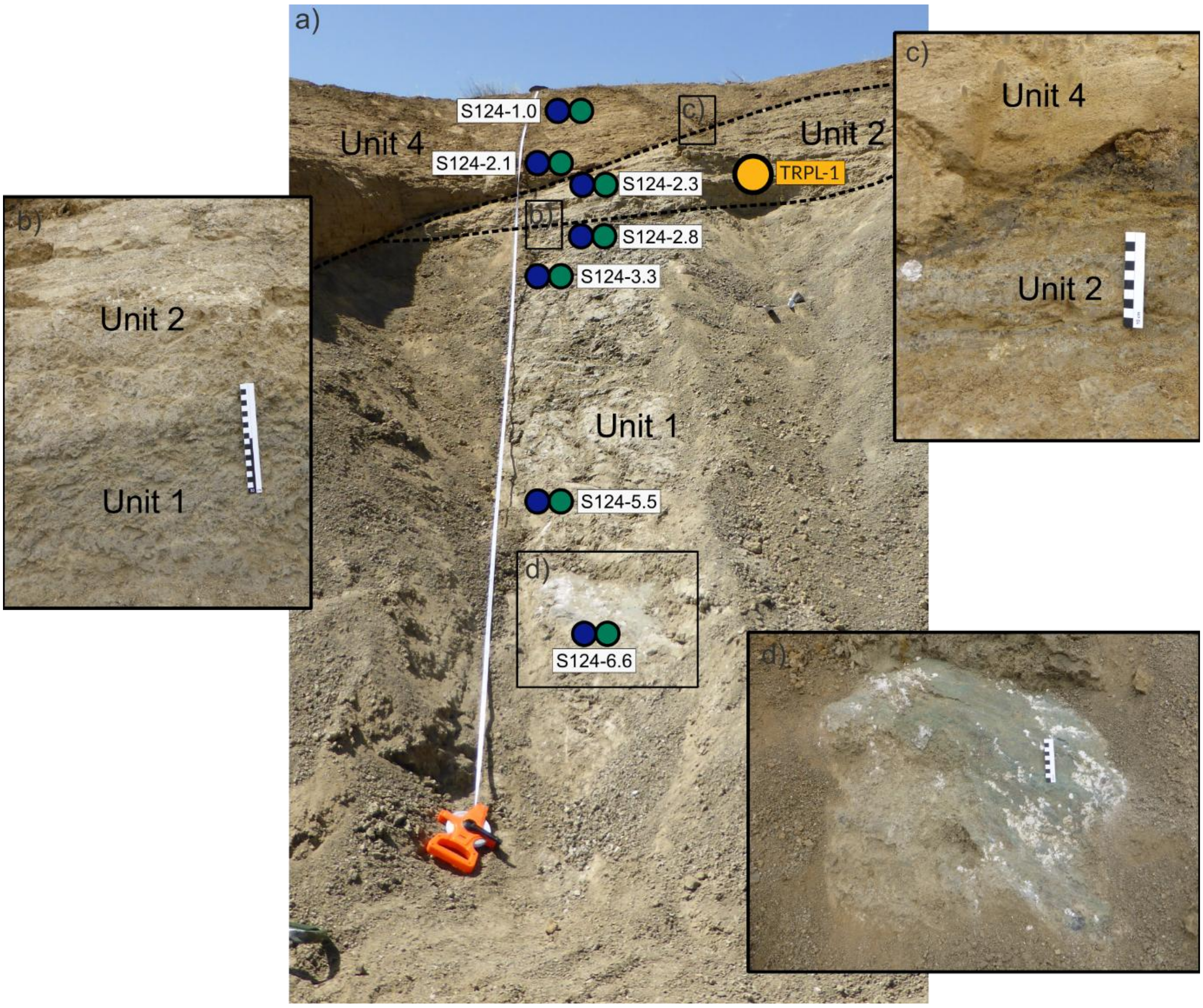

Supplementary Figure 2. Profile S124 of the Jojosi Triple Junction site with locations of luminescence (yellow), sediment texture (green), and mineralogical (blue) samples. a) full profile; b) detail of the contact between units 1 and 2; c) detail of the contacts between units 2 and 4; d) detail of clayey and greyish green mottles within the saprolite of unit 1. Measure tape = 7.5 m.

Profile S134 is exposed 10 m south of profile S124. Two gully cut-and-fill generations are nested on the saprolite base. The contact between units 1 and 3 is sharp and V-shaped at a low angle. Unit 3 is 2.00 m thick and displays a range of bedforms, including planar and wavy parallel as well as curved non-parallel, with typically well-sorted layers. Layers dominated by coarse sand or very fine sand (sandy loam) are up to 20 cm thick, have a granular structure, and their colour ranges from brown (10YR 4/3, wet) to pale brown (10 YR 6/3, dry). More silty layers are dark brown (10YR 3/3) and granular. Clay and clay loam beds are typically thinner (2–4 cm) and characterized by light greyish olive colour (10Y 6/2), a subangular blocky structure, and dark clay cutans covering aggregates. Rounded carbonate nodules up to 2 cm and pebble-sized rounded dolerite fragments account for not more than 2% of the matrix. Krotovinas indicate bioturbation. Unit 4 is deposited in a U-shaped palaeo-gully incision into

units 1 and 3 to a depth that is similar to the modern gully channel. The exposure shows a maximal height of 6,00 m. The sediments are stratified, and beds in the lower section follow the channel cross-section with a curved parallel bedding, whereas higher deposits are more parallel or slightly curved. The texture is predominantly loamy sand that breaks into subangular blocky aggregates, but also with less than 5 cm thickness of clay or sand. The colour is light olive brown (2.5Y 5/6), and clasts up to pebble size, and rounded carbonate nodules make up to 2% of the matrix. Soil formation affected the top 1 m of unit 4.

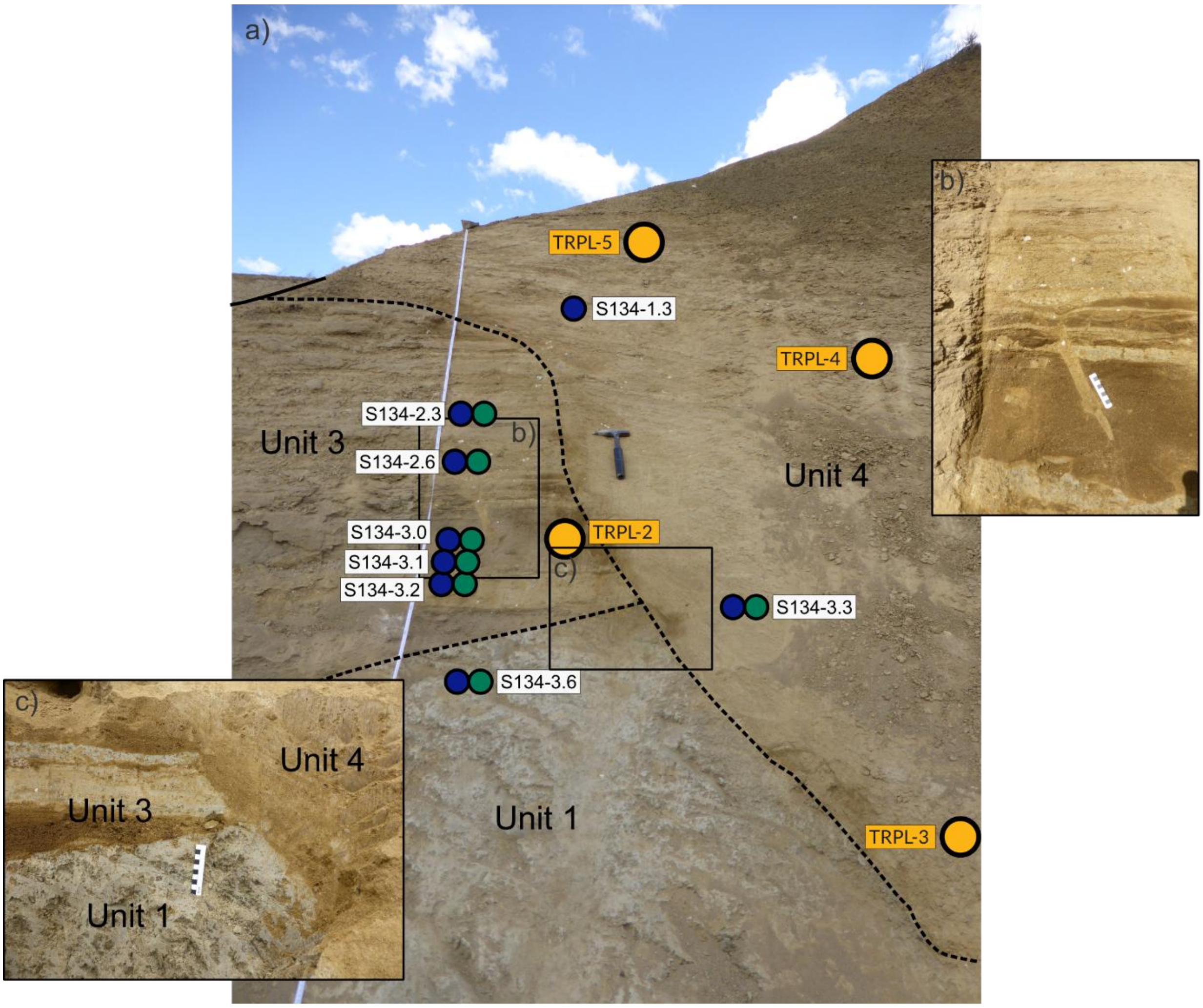

Supplementary Figure 3. Profile S134 of the Jojosi Triple Junction site with locations of luminescence (yellow), sediment texture (green), and mineralogical (blue) samples. a) full profile; b) detail of sorted and banded deposits of sedimentary unit 3. Bioturbation indicated by Krotovina; c) detail of the contact between units 1, 3, and 4. . Measure tape = 4.5 m.

3.2 Jojosi 5

Jojosi 5 is located near the top of an interfluve between two gully channels that extend downslope from the grass-covered remains of the original surface. The profile reaches a maximum height of 4.5 meters and is partially covered by dark residual vertisol and carbonate nodules in its highest areas. The lower parts, exposed farther downslope, are covered by patches of sparse vegetation. Archaeological material is located in the lower part, where the interfluve is 3.2 m high above the bottom of the gully channel.

Basal Rock Unit 1 consists of *in situ* weathered, pale greenish-grey ("olive", 5Y 5/3) saprolite that extends up to 2.1 meters below the top edge of the profile. It has a soft, clayey matrix and lacks any rock structure from the original dolerite.

The base of the overlying Rock Unit 4 is a sharp erosive surface at a depth of 2.1 m and is covered by a basal pebble line. A few sand lenses, like the one chosen for the OSL sample JOJOSI-85U, are also deposited on the bottom of this layer. Most of the section consists of beds that range from very thinly bedded to thickly laminated throughout the succession. The parallel, slightly curved bedding can be observed over distances of many meters. The material is well-cemented sandy loam that breaks into subangular, blocky aggregates. Carbonate nodules with diameters up to 5 cm and very fine (less than 2 mm) iron-manganese concretions account for less than 2% of the matrix. Rounded pebbles and boulders are rare within the succession. The archaeological material is located near the top of the profile, buried under a few decimetres of sediment.

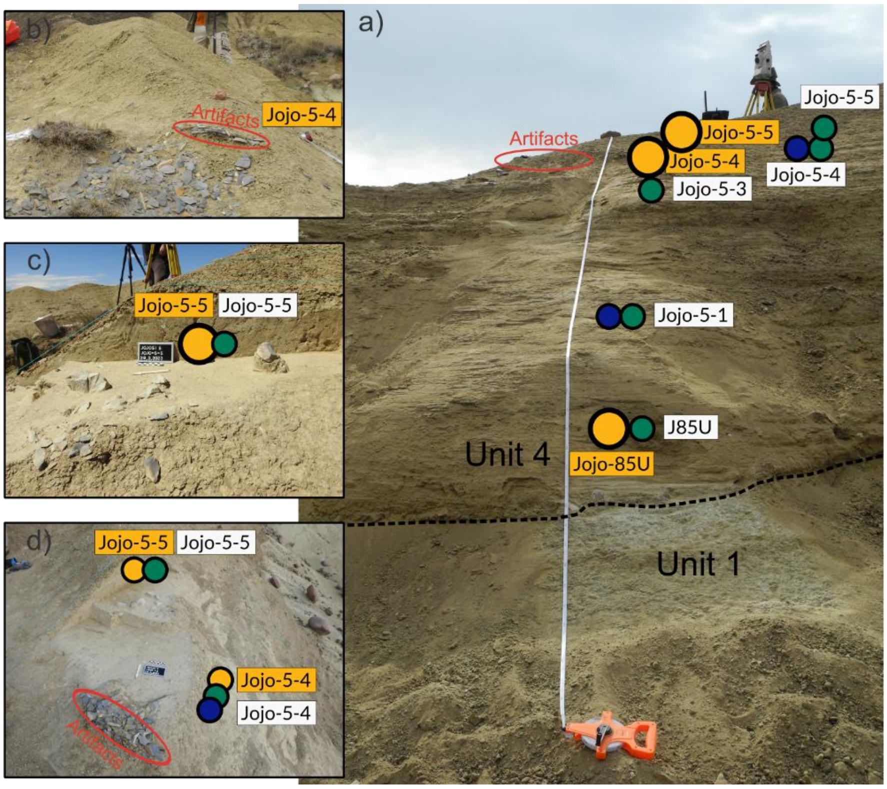

Supplementary Figure 4. Profile of the site Jojosi 5 with the locations of luminescence (yellow), sediment texture (green), and mineralogical (blue) samples. a) full profile photographed towards east; b) detail of archaeological deposits photographed towards south; c) detail of the luminescence and sediment samples Jojo-5-5 directly overlying the archaeological deposits photographed towards south. d) the luminescence and sediment samples Jojo-5-4 and Jojo-5-5 directly under- and overlie the archaeological deposits. Measure tape = 5.4 m.

3.2 Jojosi 6

The Jojosi 6 profile is located 20 meters south (upslope) from the Jojosi 5 profile and is exposed on the same rock face. This allows the units to be directly correlated. A minor gully headcut has eroded an intact remnant of the original surface here. This has created a semi-circular, half-bowl shape with a diameter of ~20 m that exposes a gully head up to 9.8 m deep. The presence of grassy vegetation on the lower slope of Unit 4 indicates stable conditions and a low erosion rate.

The deeply weathered saprolite of Unit 1 is exposed on the surface to a thickness of about 2.5 m. At a depth of 7.3 m, a sharp erosive contact with massive rounded dolerite boulders separates Units 1 and 4. Unit 4 exposed here has many sedimentary characteristics identical to those of the Jojosi 5 profile. Strongly cemented sandy loam is deposited in parallel bedding. Carbonate nodules (less than 5 cm) and very fine iron-manganese nodules (less than 2 mm) are found in the matrix alongside rare rounded pebbles. The uppermost part of Unit 4, which has a maximum thickness of 1.3 meters, is characterized by recent soil formation. The vertisol has an intact grass cover and an Av horizon. Its texture ranges from clay to silty loam, and the aggregates are angular-blocky in the subsoil. In contrast, the topsoil is granular to subangular. It can be clearly distinguished by its dark brown colour (10YR 3/3).

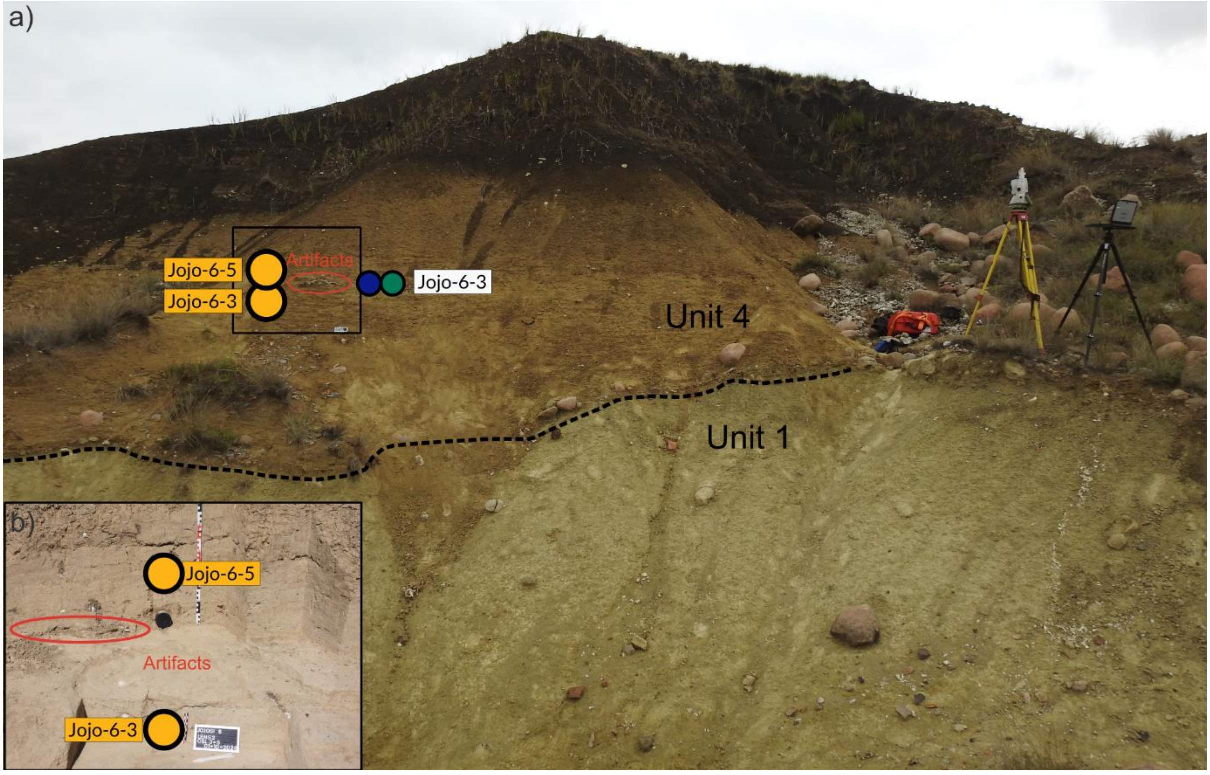

Supplementary Figure 5. Profile of the site Jojosi 6 with the locations of luminescence (yellow), sediment texture (green), and mineralogical (blue) samples. a) full profile photographed towards south; b) detail of archaeological deposits and the location of the luminescence samples. Tripod ~1.2 m.

3.3 Jojosi 1

The topography of Jojosi 1 is comparable to that of Jojosi 5, featuring an interfluve that separates two gully channels. This interfluve extends 50 meters in a northerly direction and is shielded by the remnants of the original surface that are located upslope. The interfluve is 7 m thick at its maximum and slopes downward to the north. At Jojosi 1, the ridge rises 3.05 meters above the western channel and 4.4 meters above the deeper, incised eastern channel. The southern part of the ridge is still close to the elevation of the original surface, with remains of the original vertisol subsoil covering the top. Images from the 1990s excavations[3] show that the vertisol residuals extended farther south then, but they have since been eroded. Hundreds of large, both rounded and angular, clasts, as well as hundreds of lithic artifacts, remain as lag debris in the channels.

The basal saprolite (Unit 1) starts at a depth of 2.2 m. It is of pale greenish-grey colour and darker when wet (5GY 5/2). Green mottles run through this bed. The texture is clayey and forms subangular to angular blocky aggregates. The contact with the overlying Unit 4 at a depth of 2.2 m is sharp erosive and marked by a line of boulders directly above the contact, which is well exposed from the deeper incised eastern channel. Unit 4 is reddish-brown (10YR 3/2). Thinly bedded and laminated layers of sandy loam are deposited in discontinuous curved beds. Subrounded carbonate nodules with up to 5 cm in diameter account for less than 5% of the matrix. Iron-manganese concretions (< 1 mm) and cutans thereof on sediment aggregates and ped surfaces are present. Larger clasts are virtually absent. The archaeological material, excavated in the early 1990s, was deposited ca. 0.5 m below the current top of the profile.

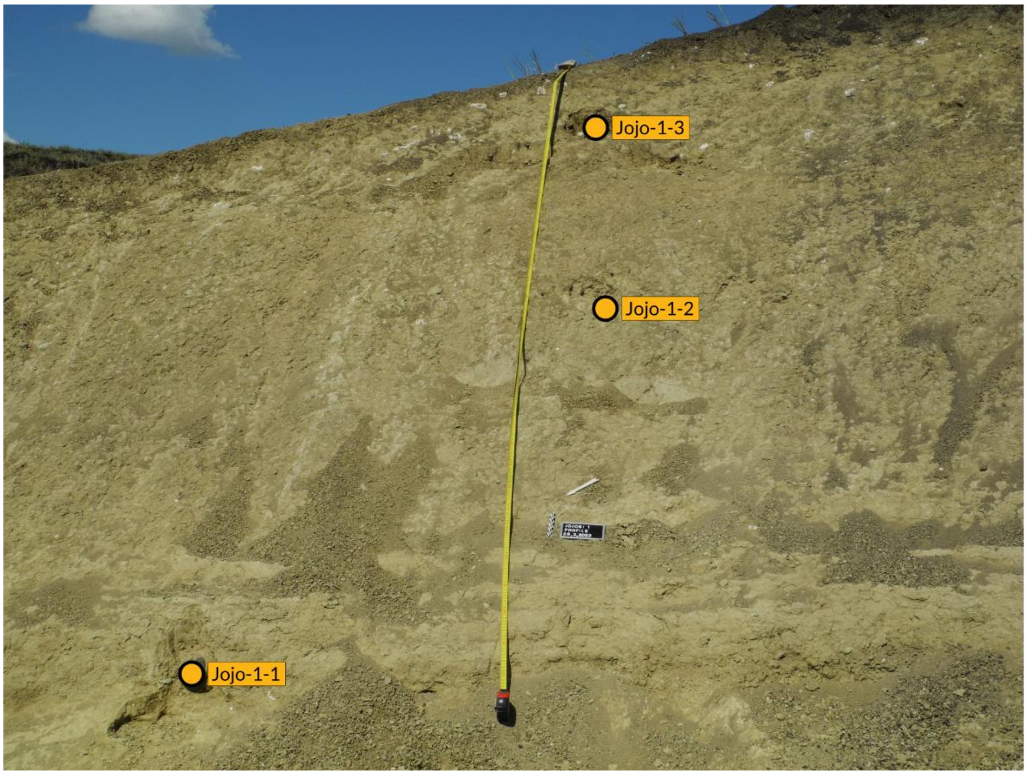

Supplementary Figure 6. Profile of the site Jojosi 1 with the locations of luminescence (yellow), sediment texture (green), and mineralogical (blue) samples. The former location of the archaeological deposits excavated by A. Mazel in the early 1990s was reconstructed to lie between the luminescence samples Jojo-1-2 and Jojo-1-3. Measure tape = 3.8 m.

3.4 Jojosi 7

Jojosi 7 is located approximately 30 meters northwest of Jojosi 1. The archaeological material is found in the remains of a small, elongated sediment bar that is 8 m long, 4 m wide, and 3 m high above the channel bottom. It was likely an extension of the interfluve that held Jojosi 1, but later became disconnected.

Unit 1 starts at a depth of 1.0–1.1 meters. The deeper parts are more clay-enriched, while the shallower parts have a loamier texture. Greenish mottles and slickensides are present, and the sediment breaks down into angular and subangular, blocky aggregates. The boundary with underlying Unit 4 is a sharp, erosive one associated with pebbles. Unit 4 has a maximum thickness of 1.1 meters and is affected by the processes of vertisol soil formation. The A horizon spans the top 10 cm and is characterized by a dark brown colour (10 YR 3/3), a clay

texture, and loose, granular-to-subangular aggregates. Fine root channels are abundant. The Bv horizon ranges from 10 to 70 cm. It is yellow-brown (10YR 5/4) in colour and consists of a clay matrix that breaks into angular, blocky aggregates. Unit 4 has a C horizon that ranges between 70 and 105 cm deep. It is characterized by a transition to a sandy texture. It breaks down into platy to subangular aggregates and is yellow-brown in colour (10YR 5/8). The second C horizon develops in the uppermost part of Unit 1, at depths greater than 105 cm. It is grey-olive in colour (10 Y 5/2), and the clayey matrix breaks into angular, blocky aggregates. The A horizon is especially susceptible to swelling and shrinking when wetted. Discontinuous cracks are found in the A and B horizons. Furthermore, the skeletal content of these horizons decreases from the top and accounts for up to 5% of the matrix. Carbonate nodules up to 5 cm in size are found in the A, B, and first C horizons. There, the sediment did not react to calcium carbonate testing with 10% HCl. However, the C horizon (Unit 1), where carbonate nodules are absent, reacts with hydrochloric acid.

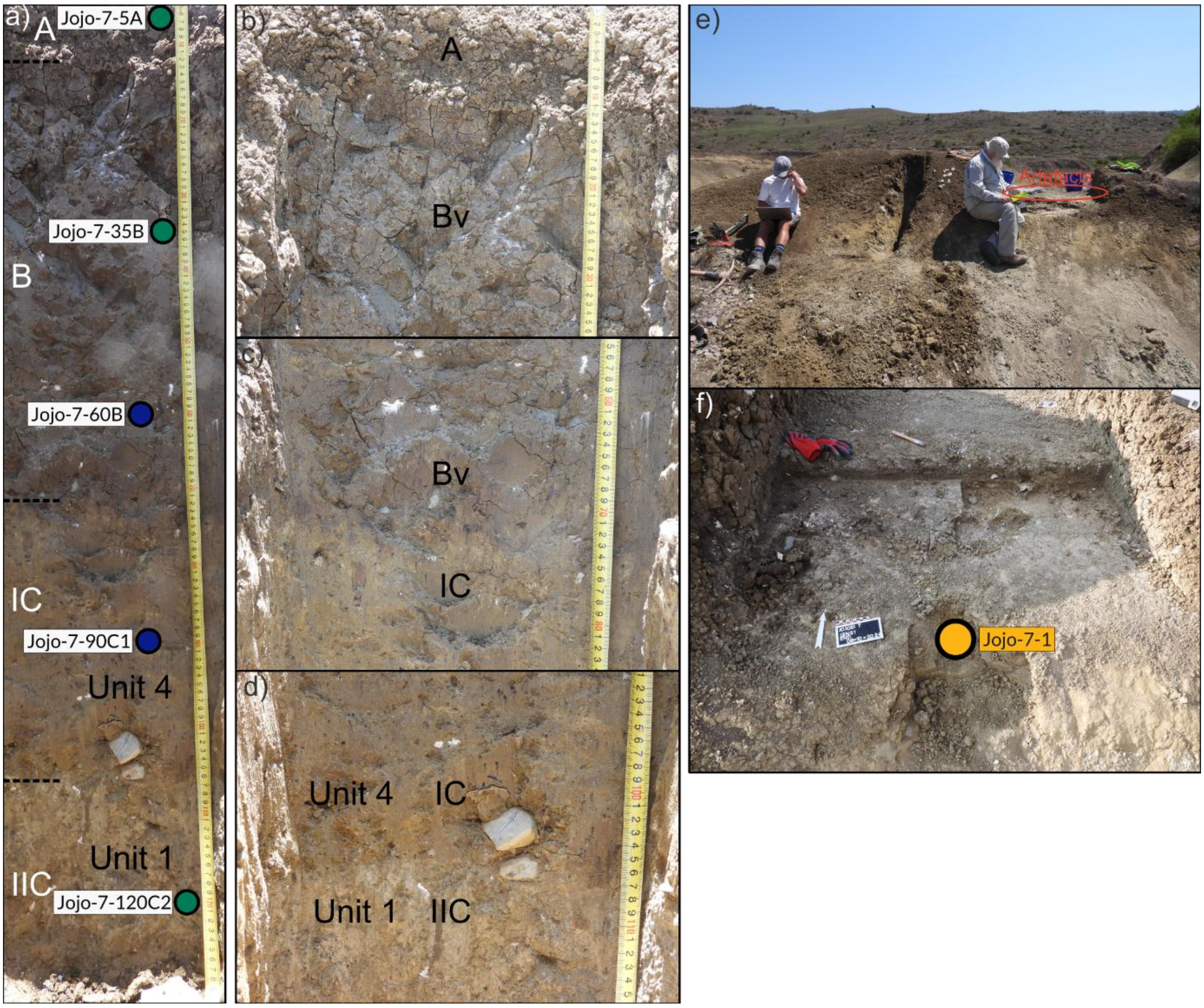

Supplementary Figure 7. Profile of the site Jojosi 7 with the locations of luminescence (yellow), sediment texture (green), and mineralogical (blue) samples. a) full profile; b) detail of the contact between soil horizons A and B; c) detail of the contact between soil horizons B and IC; d) contact between the soil horizons IC (Rock Unit 4) and IIC (Rock Unit 1); e) overview over the site with locations of the sediment profile (left) and the archaeological excavation (right); f) detail of the luminescence sample Jojosi 7 directly underlying the archaeological remains. Measure tape = 1.3 m.

4. Soil sampling results

4.1 Sediment texture results

Supplementary Table 1: Results of the full fraction sediment texture analysis carried out by the CEDARA-College of Agriculture, Hilton, South Africa.

Sample ID	Profile	Stratigraphic unit	Lab no.	Clay (% cor.)	Coarse Silt (% cor.)	Fine Silt (% cor.)	Coarse Sand (% cor.)	Medium Sand (% cor.)	Fine Sand (% cor.)	Very fine Sand (% cor.)	Silt (% cor.)	Sand (% cor.)
J85 U	Jojosi 5	RU 4	FS90	9	3	8	11	32	29	8	12	80
Jojo-5-1	Jojosi 5	RU 4	FS14	11	5	12	4	23	33	13	16	73
Jojo-5-3	Jojosi 5	RU 4	FS16	23	7	22	4	17	18	10	29	48
Jojo-5-4	Jojosi 5	RU 4	FS17	19	3	16	7	27	19	8	20	61
Jojo-5-5	Jojosi 5	RU 4	FS364	28	7	14	6	25	18	1	21	51
Jojo-6-3	Jojosi 6	RU 4	FS365	19	10	14	6	24	23	5	23	58
Jojo-7-5A	Jojosi 7	RU 4	FS358	58	4	8	7	11	5	8	12	30
jojo-7-35B	Jojosi 7	RU 4	FS359	54	9	15	4	8	5	5	24	22
Jojo-7-120C2	Jojosi 7	RU 4	FS362	53	5	21	3	6	7	4	26	20
Jojo-S124-1.0	S124	RU 4	FS26	17	5	15	6	28	21	8	20	63
Jojo-S124-2.1	S124	RU 4	FS27	16	5	15	7	20	25	12	20	64
Jojo-S124-2.3	S124	RU 2	FS28	5	5	7	16	31	25	12	11	84
Jojo-S124-2.8	S124	RU 1	FS30	66	5	17	2	4	4	3	22	12
Jojo-S124-3.3	S124	RU 1	FS31	11	5	13	9	25	24	13	18	71
Jojo-S124-5.5	S124	RU 1	FS33	62	6	21	1	3	4	3	27	11
Jojo-S124-6.6	S124	RU 1	FS34	59	6	24	1	3	4	2	31	10
Jojo-S134-3.6	S134	RU 1	FS84	41	25	21	3	3	5	2	46	13
Jojo-S134-3.2	S134	RU 3	FS40	40	4	43	2	4	5	3	47	13
Jojo-S134-3.0	S134	RU 3	FS38	2	2	4	12	58	19	3	5	93
Jojo-S134-3.1	S134	RU 3	FS39	35	8	27	1	7	14	8	35	30
Jojo-S134-2.6	S134	RU 3	FS37	46	7	23	1	7	11	5	30	24
Jojo-S134-2.3	S134	RU 3	FS36	13	7	14	1	1	17	47	21	66
Jojo-S134-3.3	S134	RU 4	FS41	48	6	33	1	3	5	3	39	13

4.2 Sediment chemistry results

Supplementary Table 2: Results of the sediment chemistry analysis carried out by the CEDARA-College of Agriculture, Hilton, South Africa.

Sample ID	Profile	Stratigraphic unit	Lab No.	Sample Density (g/ml)	Phosphorus (mg/l)	Potassium (mg/l)	Calcium (mg/l)	Magnesium (mg/l)	Zinc (mg/l)	Manganese (mg/l)	Copper (mg/l)	Zinc (mg/l)	Exch. Acidity (cmol/l)	Total cations (cmol/l)	Acid sat. (%)	pH (KCl)
J85 U	Jojosi 5	RU 4	F1039	1.21	1	25	1032	2933	0.2	1	0.2	0.2	0.11	29.46	0	7.12
Jojo-5-1	Jojosi 5	RU 4	F676	1.2	1	17	984	3862	0.3	1	0.6	0.3	0.05	36.79	0	6.07
Jojo-5-3	Jojosi 5	RU 4	F678	0.98	1	76	1156	5082	0.2	1	0.6	0.2	0.06	47.85	0	6.45
Jojo-5-4	Jojosi 5	RU 4	F679	1.1	1	19	843	4492	0.3	1	0.6	0.3	0.05	41.28	0	6.36
Jojo-5-5	Jojosi 5	RU 4	F3320	1.02	1	31	850	4170	0.4	1	0.6	0.4	0.09	38.73	0	6.5
Jojo-6-3	Jojosi 6	RU 4	F3321	1.03	1	49	678	4210	0.7	1	0.5	0.7	0.05	38.21	0	6.46
Jojo-7-5A	Jojosi 7	RU 4	F3314	1.04	1	66	1939	6080	0.8	1	1.8	0.8	0.07	59.96	0	6.76
jojo-7-35B	Jojosi 7	RU 4	F3315	1.02	1	44	1621	5690	0.8	1	1.5	0.8	0.09	55.12	0	7.23
Jojo-7-120C2	Jojosi 7	RU 4	F3318	0.82	1	60	1213	4910	0.6	1	0.8	0.6	0.06	46.68	0	7.29
Jojo-S124-1.0	S124	RU 4	F688	1.03	1	14	1102	4692	0.2	1	0.7	0.2	0.06	44.21	0	6.34
Jojo-S124-2.1	S124	RU 4	F689	1.07	1	19	1047	4452	0.1	1	0.6	0.1	0.04	41.96	0	6.25
Jojo-S124-2.3	S124	RU 2	F690	0.97	1	22	1188	5212	0	1	0.6	0	0.06	48.94	0	6.18
Jojo-S124-2.8	S124	RU 1	F692	0.86	1	53	1220	6052	0.2	1	1	0.2	0.04	56.07	0	6.07
Jojo-S124-3.3	S124	RU 1	F693	0.91	1	47	1277	6712	0.2	1	0.9	0.2	0.05	61.79	0	6.08
Jojo-S124-5.5	S124	RU 1	F695	0.84	1	47	1408	6802	0	1	0.9	0	0.05	63.18	0	6.37
Jojo-S124-6.6	S124	RU 1	F696	0.83	1	46	1866	6032	0	1	0.8	0	0.06	59.14	0	7.1
Jojo-S134-3.6	S134	RU 1	F1033	0.86	1	70	1231	5593	0.1	2	0.6	0.1	0.08	52.43	0	6.45
Jojo-S134-3.2	S134	RU 3	F702	0.98	1	26	1590	5992	0	1	0.7	0	0.08	57.4	0	7.06
Jojo-S134-3.0	S134	RU 3	F700	1.43	1	5	548	2592	0	1	0.5	0	0.04	24.12	0	6.4
Jojo-S134-3.1	S134	RU 3	F701	1.06	1	21	1022	4502	0	1	0.7	0	0.05	42.26	0	6.38
Jojo-S134-2.6	S134	RU 3	F699	0.94	1	31	3227	5772	0	1	0.9	0	0.08	63.77	0	7.17
Jojo-S134-2.3	S134	RU 3	F698	1.3	1	12	879	3342	0	1	0.6	0	0.04	31.96	0	6.03

4.3 Mineralogical results

Supplementary Table 3: Results of the semi-quantitative X-Ray Diffraction Analysis carried out by M. Safi at the XRD Laboratory of the Council for Geoscience, Pretoria, South Africa.

Sample ID	Profile	Stratigraphic unit	Ortho-pyroxene (wt %)	K-Feldspar (wt %)	Plagioclase (wt %)	Quartz (wt %)	Smectite / Illite (wt %)
Jojo-5-1	Jojosi 5	RU 4	65	0	13	7	15
Jojo-5-4	Jojosi 5	RU 4	38	0	12	11	39
Jojo-6-3	Jojosi 6	RU 4	64	0	6	4	26
Jojo-7-60B	Jojosi 7	RU 4	27	0	5	8	60
Jojo-7-90C1	Jojosi 7	RU 4	32	0	5	8	55
Jojo-S124-1.0	S124	RU 4	54	3	6	6	31
Jojo-S124-2.1	S124	RU 4	57	0	10	9	24
Jojo-S124-2.3	S124	RU 2	25	0	11	19	45
Jojo-S124-2.8	S124	RU 1	0	0	8	20	72
Jojo-S124-3.3	S124	RU 1	48	0	8	9	35
Jojo-S124-5.5	S124	RU 1	0	0	7	20	73
Jojo-S124-6.6	S124	RU 1	0	0	9	21	70
Jojo-S134-3.6	S134	RU 1	0	4	7	20	69
Jojo-S134-3.2	S134	RU 3	36	2	4	7	51
Jojo-S134-3.0	S134	RU 3	80	3	5	6	6
Jojo-S134-3.1	S134	RU 3	0	5	7	27	61
Jojo-S134-2.6	S134	RU 3	0	0	17	16	67
Jojo-S134-2.3	S134	RU 3	73	4	6	1	16
Jojo-S134-1.3	S134	RU 4	43	10	9	9	29

5. Summary

The Jojosi hillslope deposits are distinct from most other Masotcheni deposits in the region, having been derived largely by the erosion of dolerite-derived regolith, whereas the hillwash deposits in the surrounding region are commonly derived from erosion of weathered Vryheid Formation sandstone and shale bedrock. The base of the Jojosi sedimentary succession is a buried, clay-rich saprolite formed within the dolerite bedrock (Unit 1). This palaeosol profile was eroded and provided the pre-weathered sediment that was deposited downslope by sheetwash and gully channel transport processes. Cyclical gully cut-and infill processes during the Middle and Late Pleistocene resulted in the accretion of a composite regolith profile in which stratified sandy loamy sheetwash deposits buried earlier incised gullies, locally preserving intact lithic knapping deposits. The deposits of Unit 4, reaching several meters in thickness, exhibit consistently thin bedding, indicative of uninterrupted sedimentary accumulation in the absence of erosional discontinuities. The archaeological materials were gently buried and consequently preserved within a low-energy depositional setting. This explains why the archaeological finds have been preserved *in situ* with the full spectrum of clast sizes intact, and were neither destroyed nor sorted by erosional processes.

The Jojosi sediments contain abundant dolerite saprolite-derived pyroxene and calcic plagioclase grains with small quantities of quartz and K-feldspar grains derived from the Vryheid Formation sandstone outcrops in the west. The abundance of pre-weathered, calcic dolerite saprolite-derived sediment created calcic sediments, which promoted diagenetic cementation and thereby ensured the long-term preservation of the site.

The uppermost ~1 m of sediments, most often associated with Unit 4, is subject to soil formation processes in portions of the area, while in other parts the soil has been removed. These soils are vertic in nature, characterized by swelling and shrinking of active clay minerals. Such dynamics cause vertical displacement of clasts within the soil profile and allow the introduction of superficial clasts into the pedon through shrinkage cracks, a phenomenon widely recognized as complicating archaeological interpretation. While sites Jojosi 1, 5, and 6 are unaffected by these processes, Jojosi 7 presents features that warrant careful consideration. At Jojosi 7, the archaeological material is concentrated as an intact lens with clasts oriented horizontally within the C horizon, indicating that downward migration of the soil profile through the archaeological layer occurred, rather than vertical displacement of artifacts from the surface downward.

Supplementary Note 2: Luminescence dating

1. Sampling strategy and procedure

Samples for luminescence dating were collected during field campaigns in 2022, 2023, and 2024, which took place in the Jojosi donga system near Nqutu, KwaZulu-Natal, in South Africa. Samples were collected by hammering opaque sample tubes into the exposed sediment sections or by carving blocks of sediment from the exposed sections. Samples for dose rate determination were collected from the sediment surrounding the luminescence sample locations, by accounting for variations in the sediment texture and colour, which might suggest variations in gamma dose rate delivered to the luminescence samples (e.g.,[4] Appendix H).

Samples were either collected directly in an archaeological context, taken in the close vicinity of the artefact lenses, to bracket the artefacts, or at locations representing the geomorphological complexity of the Jojosi donga system. In the case of Jojosi 7, it was not possible to bracket the artefact layer with luminescence samples due to the intense soil development. We thus only sampled below the artefact layer. In both the archaeological and the geomorphological case, the aim was to select sampling locations that would provide vital information for the study once the timing of their deposition was constrained.

2. Sample preparation

Samples for dose determination were prepared under subdued red-light conditions in the Cologne Luminescence Laboratory (CLL; University of Cologne). Hydrochloric acid (HCl; 10 %) and hydrogen peroxide (H_2O_2; 10 %) were used to remove carbonates and organic material, respectively. Sodium oxalate ($Na_2C_2O_4$; 0.01 N) was used to disperse the sediment particles. After chemical treatment, the samples were sieved to obtain the 200–250 µm grain size fraction. From this fraction, K-feldspar-rich extracts were separated using a sodiumpolytungstate solution at a density of 2.58 g cm^{-3}. For pre-tests, performed to determine the appropriate measurement protocol, multi-grain aliquots (4 mm in diameter) of the isolated K-feldspar fraction were mounted on stainless-steel discs using silicone oil. To obtain multi-grain equivalent dose distributions, feldspars were mounted as multi-grain aliquots (1 mm diameter, resulting in approximately <30 grains on a single disc[5]) on stainless-steel discs using silicone oil.

3. Luminescence instrumentation and measurement procedure

Luminescence measurements were performed using several Risø TL/OSL DA-20 readers[6], each equipped with a $^{90}Sr/^{90}Y$ beta source and IR LEDs operating at 90 % power (~145 mW cm^{-2} for classic head at 100 %; ~300 mW cm^{-2} for DASH at 100 %) at the CLL and at Risø (Technical University of Denmark, DTU). The feldspar luminescence signal was detected through a combination of a 2 mm thick Schott BG39 filter and a 3 mm thick Corning 7-59 filter or BG3 filter, depending on the reader, allowing the transmission of the blue emission (~410 nm[7]).

The single-aliquot regenerative (SAR) dose protocol[8] was adapted for feldspars as post-IR IRSL protocol[9] with a preheat temperature of 250 °C, a first-IR stimulation temperature of 50 °C, and a post-IR IRSL temperature of 225 °C. Details regarding the tests (dose-recovery preheat plateau, residual preheat plateau, and fading preheat plateau tests) performed to define this protocol are outlined in Riedesel et al.[10]. All steps of the selected post-IR $IRSL_{225}$ protocol are listed in Supplementary Table 4.

Supplementary Table 4. Post-IR $IRSL_{225}$ measurement protocol used for feldspar measurements. [a]For D_e measurements, a test dose of 40 Gy was used for all samples, except for JOJO-TRPL-1 and JOJO-TRPL-2, where a test dose of 75 Gy was used.

Step	Treatment	Observed
1	Beta dose	
2	Preheat 250 °C, 60 s	
3	IRSL 50 °C, 200 s	
4	post-IR IRSL at 225 °C, 300 s	L_x
5	Test dose[a]	
6	Preheat 250 °C, 60 s	
7	IRSL 50 °C, 200 s	
8	post-IR IRSL at 225 °C, 300 s	T_x

The chosen protocol was validated for all samples using a dose recovery test (including residual measurements and subtraction), with all samples exhibiting dose recovery ratios within 10 % of unity (Supplementary Figure 8). Fading was also measured for all samples using the protocol outlined in Table 4 and following the procedure by Auclair et al.[11], with pauses of 0, 1000, 10,000, and 100,000 s inserted between steps 2 and 3. The pauses of 0 s and 10,000 s were repeated at the end of the fading sequence to check for changes in sensitivity. The measurements were reproducible to ±10 %. The obtained fading rates are displayed in Figure 8 for both luminescence signals investigated. Due to the low fading rates (<2 %/decade) of the post-IR $IRSL_{225}$ signal of most samples, the obtained post-IR $IRSL_{225}$ ages were not corrected for fading (see[10], for details).

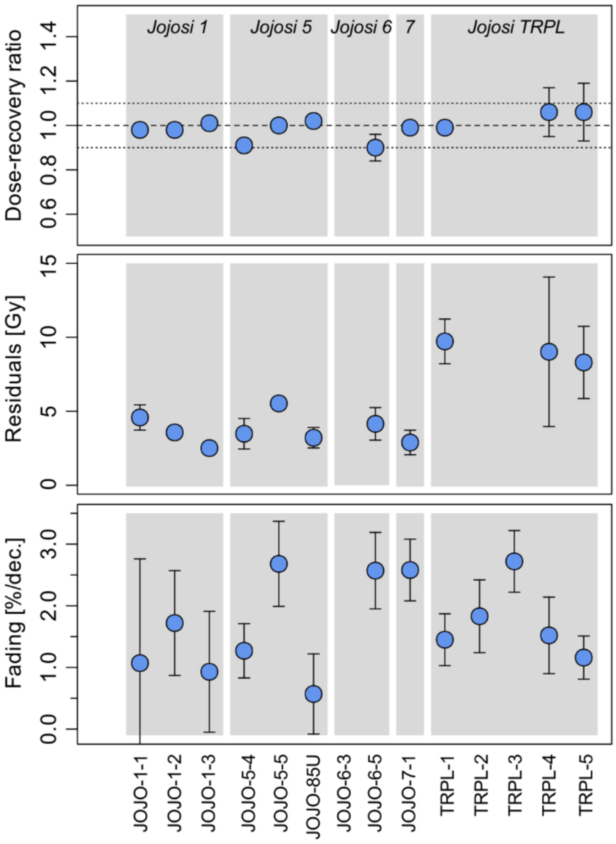

Supplementary Figure 8. Results of the protocol validation. Dose recovery ratios (residuals subtracted), residuals, and fading rates are displayed for the post-IR $IRSL_{225}$ signal for most samples. Due to limited material, not every sample was measured in each test. Empty spaces indicate where a measurement was not performed. The data points represent the average of n=3 aliquots and their standard error of the measurement. Source data are provided in the Source Data file.

To obtain an equivalent dose from the luminescence signal of multi-grain aliquots, we integrated the initial 10 s of the signal and subtracted the last 20 s as a background. Dose response curves were fitted using a single saturating exponential function, L/T=Imax (1– exp (D/D_0), where L/T is the normalised OSL signal, D is the laboratory dose, and D_0 is a curvature parameter). Equivalent doses were accepted with recycling ratios within 20 % of unity, with the relative test dose error smaller than 20 %, and with a Tn signal three standard deviations above the background. Multi-grain aliquots were regarded as saturated when the L_n/T_n ratio and/or the sum of this ratio plus its uncertainty did not intercept the dose response curve and thus lies above the maximum asymptote of the dose response curve (I_{max})[10,12-14].

Riedesel et al.[10] tested and evaluated the performance of four different dose and age models on a data set consisting of nine samples from Jojosi. Based on their results, doses and ages for all samples were calculated using the Bayesian hierarchical model BayLum[15]. BayLum was used to facilitate the R package BayLum[15]. The function Age_Computation() was run with PriorAge and Iter (iterations) suitable for each sample. Prior to accepting the results, they were evaluated for proper convergence. For this, the Gelman and Rubin test of convergence was performed (this is provided by BayLum), and it was ensured that convergence was reached for the age, dose, and the dose dispersion parameters (all below 1.05[15]). Furthermore, the BayLum-provided graphical output, which provides a means of evaluating the MCMC trajectories, was visually inspected. The final ages were calculated using BayLum with stratigraphic control by employing the AgeS_Computation() function available in the BayLum package. Since the lateral correlations between the different sampling sites within the Jojosi donga system are not fully clear, stratigraphic information (indicating dependencies in depth, i.e., above or below a certain sample) was only used within each profile.

4. Dose rate determination

To estimate the external dose rate delivered to the luminescence sample from the surrounding sediment, uranium (U), thorium (Th), and potassium (K) contents were determined by high-resolution gamma spectrometry. An airtight container was filled to maximum capacity with approximately 200 g of dried, homogenised sediment. The filled containers were stored for at least 4 weeks to compensate for radon loss induced by sample preparation, before measurement with an Ortec Profile MSeries GEM Coaxial P-type high-precision Germanium Gamma-Ray detector. Measurement time in this study was 200,000 s. Peak selection for activity calculations included the following peaks: ^{232}Th decay series of 338, 911, 969, 239, and 583 keV; ^{238}U decay series of 295, 352, 609, and 1765 keV; and 40K of 1461 keV. For all luminescence and dosimetry samples, the water content was determined by weighing the freshly collected wet sample and comparing this weight to its weight after drying the sediment for 2 days at 45 °C. The average water content ±standard error of all measurements is 15 % ±2 %. However, to account for unknown but realistic fluctuations in water content in the past, a water content of 15 % ±5 % was used for all samples. We reason that this water content represents probable scenarios over the dated age range: Firstly, we collected the samples in autumn, and rain events had taken place prior to our field campaign, likely wetting the sediments. Krapp et al.[16] showed that the mean annual temperature only varied between 5-10 % for our period of interest. Furthermore, our luminescence dating results indicate that similar environmental conditions resulting in cut-and-fill processes and the donga landscape must have been present over at least the past ~600 ka, resulting in well-drained dongas.

The internal K-concentration was determined using a Risø GM multicounter system[17]. For the measurements on the multicounter system, up to two sample carriers (dependent on material

availability) for each sample were prepared following Bøtter-Jensen and Mejdahl[17], by distributing 100 mg of sample material on the top of an upside-down sample carrier. One sample carrier was loaded with 100 mg of K-feldspar standard (FK-N bought via Service d'Analyse des Roches et des Minéraux, K_2O-concentration 12.81 %, Rb-concentration 860 ppm[18], and one sample carrier was prepared with 100 mg finely ground sucrose (icing sugar) to act as blank measurement. The counting rates (48 h) of two subsamples of the feldspar separates from all nine samples were compared to the counting rates obtained for a K-feldspar standard (FK-N[18]) as the basis for the K-concentration determination. For these calculations, it was assumed that the K to Rb ratio in the samples was similar to the ratio within the standard and that the detected beta radiation was emitted by K and Rb, neglecting potential small contributions from internal U and Th. The average ± standard deviation of the two measurements per sample was used for internal dose rate calculations.

Dose rate and age calculations were performed using the Dose Rate and Age Calculator (DRAC[19]). For samples JOJO-TRPL-2 and JOJO-TRPL-3, a user-defined gamma dose rate was calculated using the scale_GammaDose function[20] available in the R Luminescence package to account for variations in gamma dose rate between layers influencing these two luminescence samples. The dose rate conversion factors by Guérin et al.[21] were used to convert U, Th, and K concentrations into dose rates. Alpha and beta grain size attenuation factors following Bell[22] and Guérin et al.[23] were applied, respectively. An alpha efficiency of 0.11 ±0.03[24] was used. The cosmic dose rate was calculated according to Prescott and Hutton[25], using longitude, latitude, and altitude values gathered in the field using GPS. Details regarding the external U, Th, and K concentrations, the internal K concentrations, and total dose rates are given in Supplementary Table 5.

Supplementary Table 5. Results of the dose rate determination procedures. U, Th, and K contents were determined using high-resolution gamma spectrometry. Internal K concentration was determined using beta counting. Sampling depths were measured in the field. Total dose rates ($\dot{D}$) were calculated using DRAC[19].

Sample ID	CLL No	U [ppm]	Th [ppm]	K [%]	Internal K [%]	Depth [m]	Total $\dot{D}$ [Gy ka-1]
Jojosi 1							
JOJO-1-1	C-L5531	0.48 ± 0.04	2.66 ± 0.19	0.30 ± 0.01	0.74 ± 0.06	3.65 ± 0.05	0.74 ± 0.03
JOJO-1-2	C-L5532	0.42 ± 0.03	2.48 ± 0.18	0.29 ± 0.01	0.76 ± 0.13	2.00 ± 0.05	0.74 ± 0.03
JOJO-1-3	C-L5533	0.46 ± 0.03	2.65 ± 0.19	0.28 ± 0.01	0.90 ± 0.02	0.65 ± 0.05	0.81 ± 0.03
Jojosi 5							
JOJO-85U	C-L5349	0.39 ± 0.03	1.97 ± 0.14	0.28 ± 0.01	2.11 ± 0.09	2.00 ± 0.05	0.8 ± 0.03
JOJO-5-4	C-L5520	0.39 ± 0.03	2.29 ± 0.17	0.29 ± 0.01	0.87 ± 0.03	0.6 ± 0.05	0.78 ± 0.03
JOJO-5-5	C-L5521	0.41 ± 0.03	2.34 ± 0.17	0.28 ± 0.01	0.96 ± 0.07	0.35 ± 0.05	0.81 ± 0.04
Jojosi 6							
JOJO-6-3	C-L5933	0.48 ± 0.04	2.45 ± 0.18	0.33 ± 0.01	1.73 ± 0.62	5.90 ± 0.10	0.79 ± 0.05
JOJO-6-5	C-L5935	0.4 ± 0.03	1.97 ± 0.14	0.27 ± 0.01	1.73 ± 0.62	5.59 ± 0.10	0.70 ± 0.05
Jojosi 7							
JOJO-7-1	C-L5936	0.58 ± 0.04	3.79 ± 0.27	0.3 ± 0.01	0.91 ± 0.01	0.9 ± 0.05	0.91 ± 0.04
Jojosi Triple (TRPL)							
JOJO-TRPL-1	C-L5536	0.41 ± 0.03	2.85 ± 0.2	0.45 ± 0.01	0.72 ± 0.02	1.20 ± 0.05	0.92 ± 0.04
JOJO-TRPL-2	C-L5537	0.31 ± 0.02	1.49 ± 0.11	0.24 ± 0.01	1.15 ± 0.1	3.49 ± 0.05	0.60 ± 0.03
JOJO-TRPL-3	C-L5538	0.43 ± 0.03	2.68 ± 0.19	0.36 ± 0.01	1.71 ± 0.01	4.37 ± 0.05	0.82 ± 0.04
JOJO-TRPL-4	C-L5539	0.47 ± 0.03	2.82 ± 0.2	0.34 ± 0.01	1.09 ± 0.18	2.82 ± 0.05	0.82 ± 0.03
JOJO-TRPL-5	C-L5540	0.44 ± 0.03	2.63 ± 0.19	0.29 ± 0.01	1.09 ± 0.18	1.67 ± 0.05	0.79 ± 0.03

5. Luminescence dating results

Using BayLum, we calculated feldspar post-IR $IRSL_{225}$ multi-grain aliquot ages for all 20 samples from the Jojosi dongas. Details regarding the BayLum doses and ages, as well as the number of accepted and saturated aliquots, can be found in Supplementary Table 6 for each sample. The feldspar luminescence ages taken from sections with archaeological context (Jojosi 1, Jojosi 5, Jojosi 6, and Jojosi 7) constrain human activities within the area (see Figure 2 Main Text). Knapping sites discovered within these four different archaeological sites have different timings. The oldest knapping site within the Jojosi donga system is Jojosi 6. Here, the sample JOJO-6-3 (222-258 ka) and JOJO-6-5 (201-235 ka) bracket the artefact lens. Jojosi 1 is the youngest, with luminescence ages bracketing human activities at this site to 106 to 139 ka. Knapping at Jojosi 5 and Jojosi 7 occurred in between the activities at Jojosi 1 and Jojosi 6, showing that the Jojosi dongas were repeatedly visited by modern humans over tens of thousands of years from at least ~220 ka until at least ~110 ka. The Jojosi dongas as an environment are characterised by sedimentation and erosion, with cut-and-fill processes shaping the landscape since at least ~650 Myr (see Figure 2 Main Text).

Supplementary Table 6. Results of luminescence age calculations using BayLum. The total dose rates are given as orientation. Furthermore, the number of accepted ($n_{accepted}$) and saturated ($n_{saturated}$) aliquots is given in the table. $n_{accepted}$ includes $n_{saturated}$. For BayLum doses and ages 1σ ranges are given, which represent the 68 % credible interval calculated using BayLum. The doses are based on calculations of the individual samples. The ages were calculated with stratigraphic control.

Sample ID	CLL No	Total $\dot{D}$ [Gy ka^{-1}]	$n_{accepted}$ ($n_{saturated}$)	BayLum Dose [Gy]	BayLum Dose [Gy, 1σ range]	BayLum age [ka]	BayLum age [ka, 1σ range]
Jojosi 1							
JOJO-1-1	C-L5531	0.74 ± 0.03	30 (0)	112	101-119	156	141-166
JOJO-1-2	C-L5532	0.74 ± 0.03	35 (0)	96	94-99	132	121-139
JOJO-1-3	C-L5533	0.81 ± 0.03	28 (0)	89.5	85.3-91.3	111	106-117
Jojosi 5							
JOJO-85U	C-L5349	0.8 ± 0.03	35 (2)	180	163-197	218	203-242
JOJO-5-4	C-L5520	0.78 ± 0.03	34 (0)	135	125-137	175	160-187
JOJO-5-5	C-L5521	0.81 ± 0.04	28 (0)	119	114-125	148	136-160
Jojosi 6							
JOJO-6-3	C-L5933	0.74 ± 0.05	32 (0)	182	170-192	242	222-258
JOJO-6-5	C-L5935	0.64 ± 0.05	30 (0)	156	147-166	219	201-235
Jojosi 7							
JOJO-7-1	C-L5936	0.91 ± 0.04	32	205	189-221	226	204-245
Jojosi Triple (TRPL)							
JOJO-TRPL-1	C-L5536	0.92 ± 0.04	39 (14)	568	546-581	622	583-654
JOJO-TRPL-2	C-L5537	0.6 ± 0.03	35 (1)	309	286-330	526	479-561
JOJO-TRPL-3	C-L5538	0.82 ± 0.04	34 (3)	212	202-224	256	236-273
JOJO-TRPL-4	C-L5539	0.82 ± 0.03	36 (0)	184	177-189	236	224-246
JOJO-TRPL-5	C-L5540	0.79 ± 0.03	30 (2)	195	178-205	224	213-237

Supplementary Note 3: Excavations and archaeology of the Jojosi Dongas

Supplementary Discussion 1. Surface archaeology of the Jojosi Dongas and hornfels outcrop

Supplementary Figures 9, 10, and 12 give an impression of the enormous amount and MSA character of the surface archaeology encountered within the donga landscape. Supplementary Figure 11 shows the hornfels outcrop and secondarily derived angular blocks in the dongas. For more details on all aspects of surface archaeology that are not part of this article, see Will et al.[26].

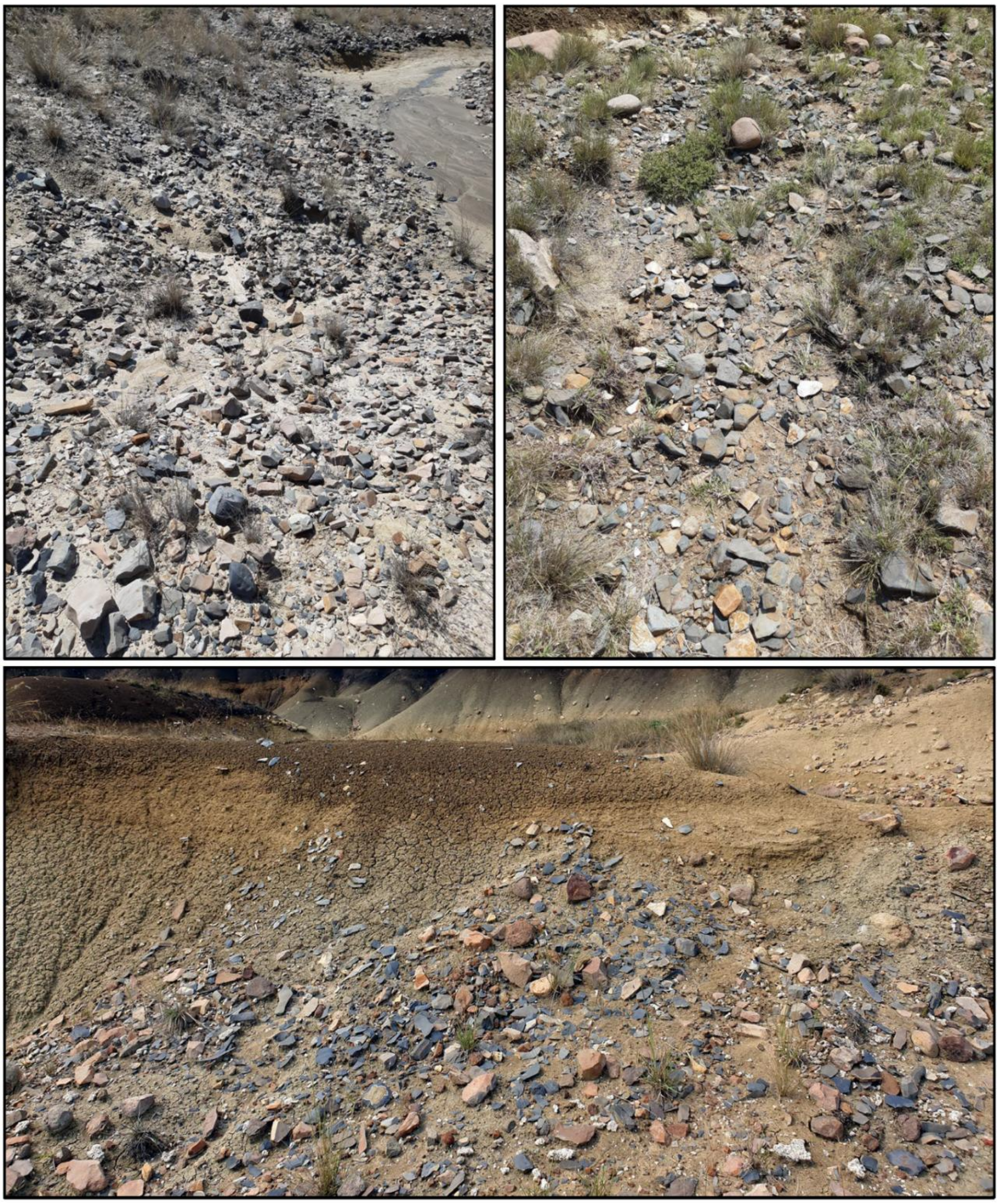

Supplementary Figure 9. Typical high-density scatters of stone tools encountered during surveys in 2022 and 2023 within the Jojosi dongas, with the majority of material belonging either to the MSA or being undiagnostic. All grey and reddish stones (surface patina) visible on the surface are flaked hornfels artefacts, counting many thousands only in the depicted areas of several square meters. The bottom shows an MSA surface concentration with a particularly high density of material and freshly outcropping hornfels artefacts from a mostly eroded artefact lens.

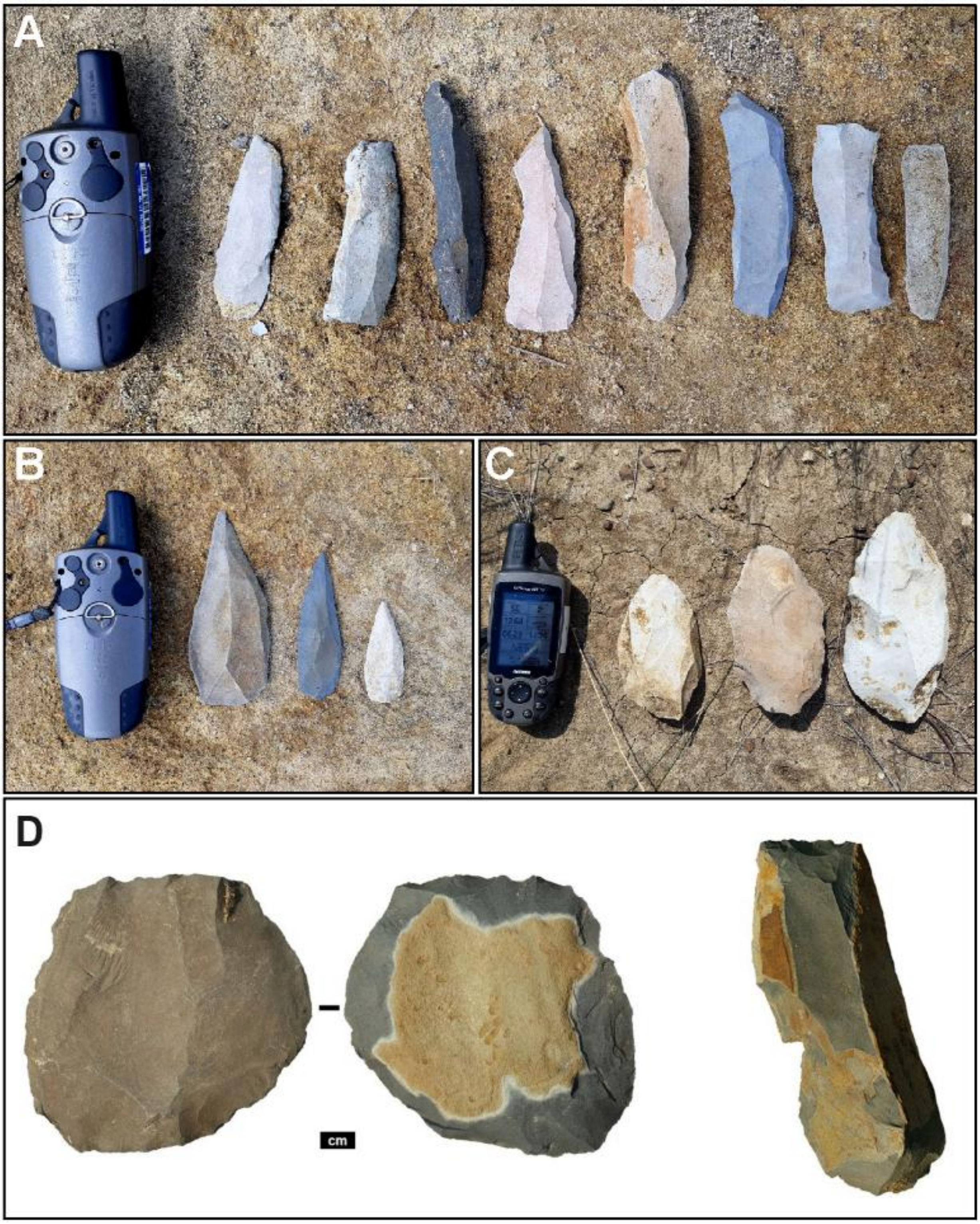

Supplementary Figure 10. Selection of stone tools from the surface of the Jojosi Dongas with a handheld GPS for scale (height 15.5 cm). Large blades (a) and unifacial points (b) selected from MSA concentrations in the main study area. Handaxes from the ESA surface concentration in the northern part of the dongas, beyond the main area of this study (c). Large, recurrent *Levallois* core (left) and unidirectional blade platform core (d). All artefacts are made of hornfels

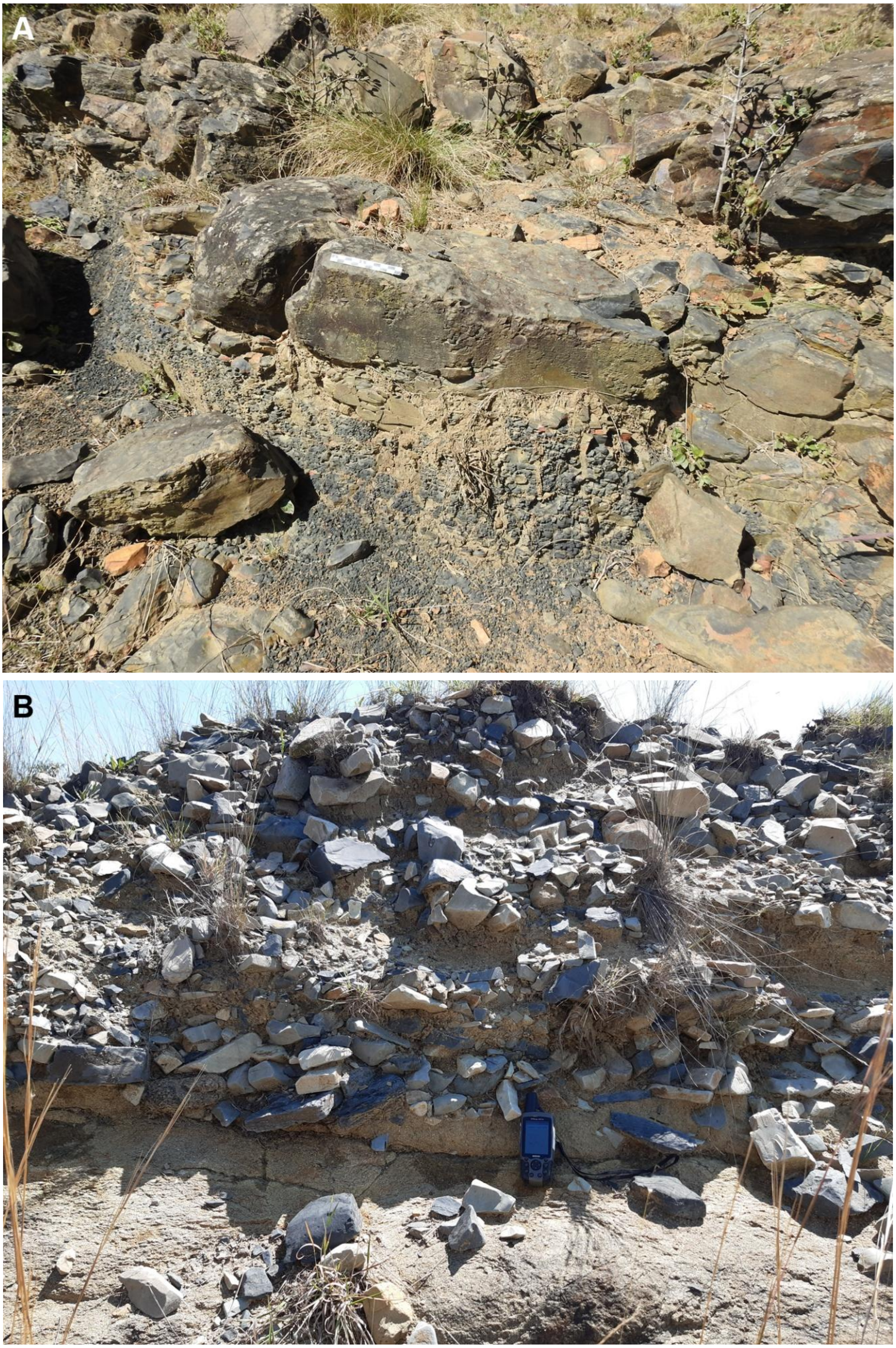

Supplementary Figure 11. The hornfels raw material sources at Jojosi. A) Primary outcrop ca. 500 m west of the active dongas (see also Figure 1c of the main manuscript). B) Example of large angular secondary blocks of raw materials washed into the dongas from primary sources.

Supplementary Discussion 2. Methods of excavations and findings at Jojosi 5, 6, and 7

The open-air setting of Jojosi and the specific donga sediments required adjustments to systems of excavations as usually employed at sheltered Stone Age sites in South Africa. The sediments at Jojosi lack visible stratigraphy/layering, do not form large, even horizontal surfaces such as in cave contexts, and we anticipated no archaeological remains other than those that could be spotted as lenses on the slopes from the outside. Based on these conditions, we excavated the open-air deposits in stratigraphic ‘contexts’ (see below) within arbitrary 25 cm wide transects as spatially delineated excavation units rather than using a traditional square meter grid system. Supplementary Figure 13 provides an image of the transects as used in the field. If the sediments within one context of one transect exceeded 10 cm, we dug them in large spits (typically 10 cm) and down from the surface of the sediments until reaching the lenses of stone artefacts. Once the artefact lenses were reached, the excavation stopped and continued in adjacent areas to carefully uncover the full extent of the stone tool lens following its three-dimensional geometry. Then, the lens was carefully excavated with brushes and small trowels, and stone tools were measured *in situ* with a Total Station and then removed until the bottom of the artefact concentration was reached. The following contexts were defined: Surface as ‘Surface’. Sediments above and around the lenses were recorded as ‘Overburden’, the lenses themselves as ‘Lens’. Lenses consist of multiple layers of stone artefacts stacked on one another in spatially confined zones. The context was recorded for all excavated buckets of sediments and for individually measured finds.

In all contexts, excavators left all stone tools or other remains >2 cm in place. These artefacts are first photographed (including SFMs of entire excavated surfaces) and then piece-plotted using a Total Station and the excavation software EDM developed by Dibble and McPherron. Smaller finds were collected by screening the recovered buckets of sediment. The provenience of materials recovered from the screens can be located to within a 25 cm transect and a thickness of a maximum of 10 cm. In the field and directly following their excavation, crew members used a coarse screen with a mesh of 5 mm and a fine screen with a mesh of 1 mm for sieving the sediment buckets. To be more exact, the 5 mm mesh has two holes per centimetre. The wire is thick, so the actual holes are about 3 mm on a side. The 1 mm mesh has six holes per centimetre, and the actual hole is about 1 mm on a side. Most of the sediments lacked any remains >1 cm at all, with by far the most abundant find category being stone tools and those concentrated within the lenses. Faunal remains were few or absent.

In the field lab, most attention was directed towards sieving sediments that could not be sieved in the field and on sorting the resulting artefacts. We classified the lithic finds into general size classes, including micro-artefacts (1-5 mm) and smaller artefacts (6-10 mm and 11-20 mm). These groupings reflect pragmatic categories that are used to document the large numbers of artefacts that need to be processed. Finds larger than 20 mm are upgraded and treated as single finds in tables and numerical studies, as are tools, tool fragments, and cores of all sizes. Preliminary lithic documentation in the field consisted of basic typological assessments, counts, and raw material identification.

Jojosi 5

In 2023, the first new excavations started on a rich lens of artefacts we had found during surveys in 2022, which we now named as Jojosi 5. A. Mazel had dug at four different spots in the 1990s, which we now refer to as Jojosi 1-4. As a first step, we documented the outline of surface finds just eroding out of the first lens of Jojosi 5 with a Total Station and then collected all surface finds regardless of size, also scraping the 2 cm of the surface and sieving the

resulting sediments (Supplementary Figure 12). These finds are all labelled as coming from the context ‘Surface’ and amount to a total of n=8061 stone tools exclusively made from hornfels (n=2106 pieces <10 mm). We then established a new excavation grid, aligning the 25 cm transect by an X-axis, and started excavating the sediments of the ‘Overburden’ down to the top of Lens 1 (Supplementary Figure 13). Detailed sediment descriptions of the individual sites can be found in Supplement Information 1.

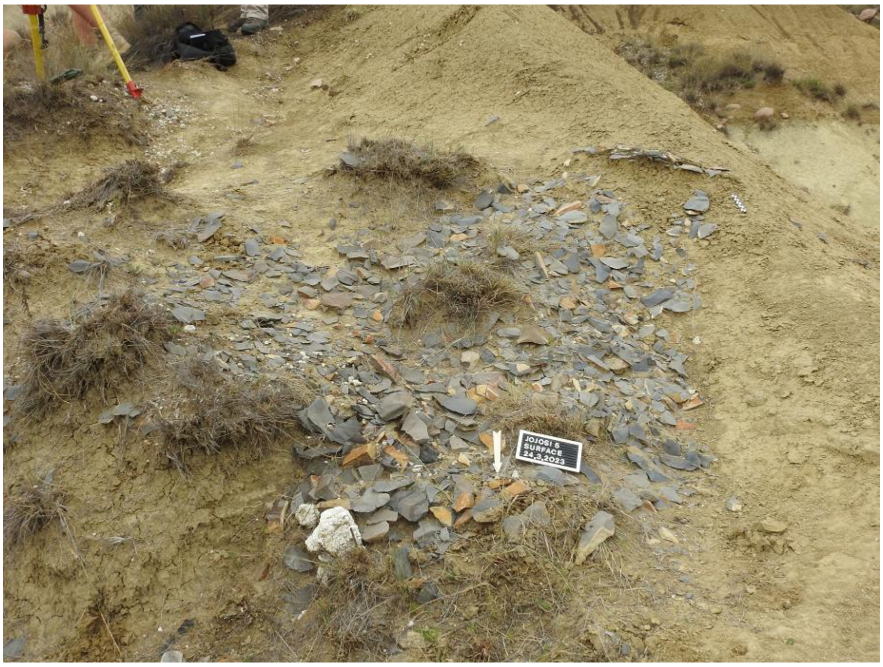

Supplementary Figure 12. Surface of Jojosi 5, Lens 1. Note the high density of recently eroded hornfels artefacts on the surface. The remaining lens can be seen on the top right, still *in situ.* Systematic collection of this area (~6 m²) retrieved 8061 finds with n=2811 artefacts (>20 mm), n=3090 (20-11m), and n=2160 <10 mm.

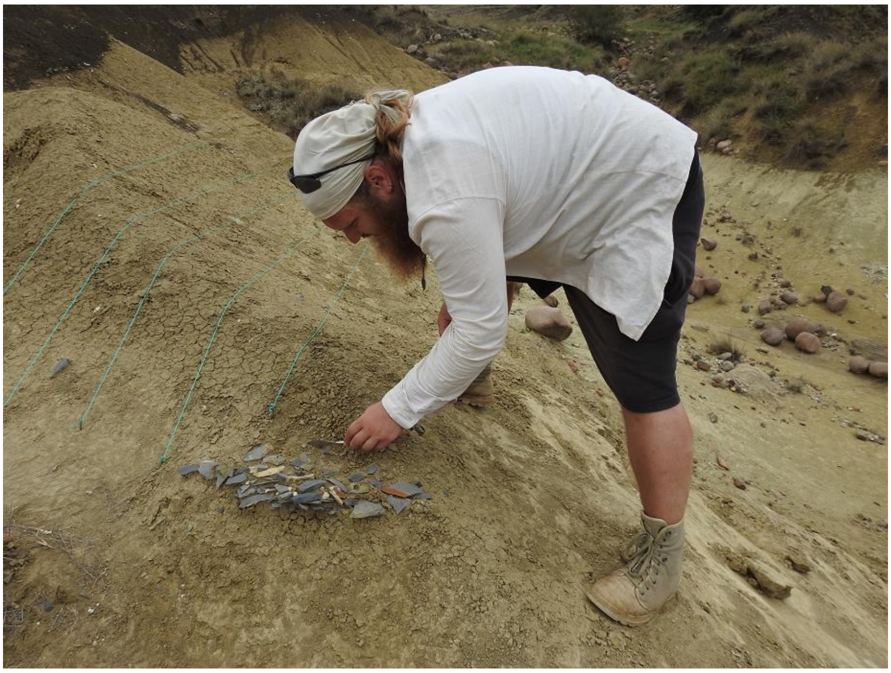

Supplementary Figure 13. Excavation of Lens 1 at Jojosi 5 by Matthias Blessing, showing the arbitrary 25 cm spatial excavation units (“transects”) indicated with green strings and the beginning of the excavations of the Overburden sediments on top of the artefact concentration.

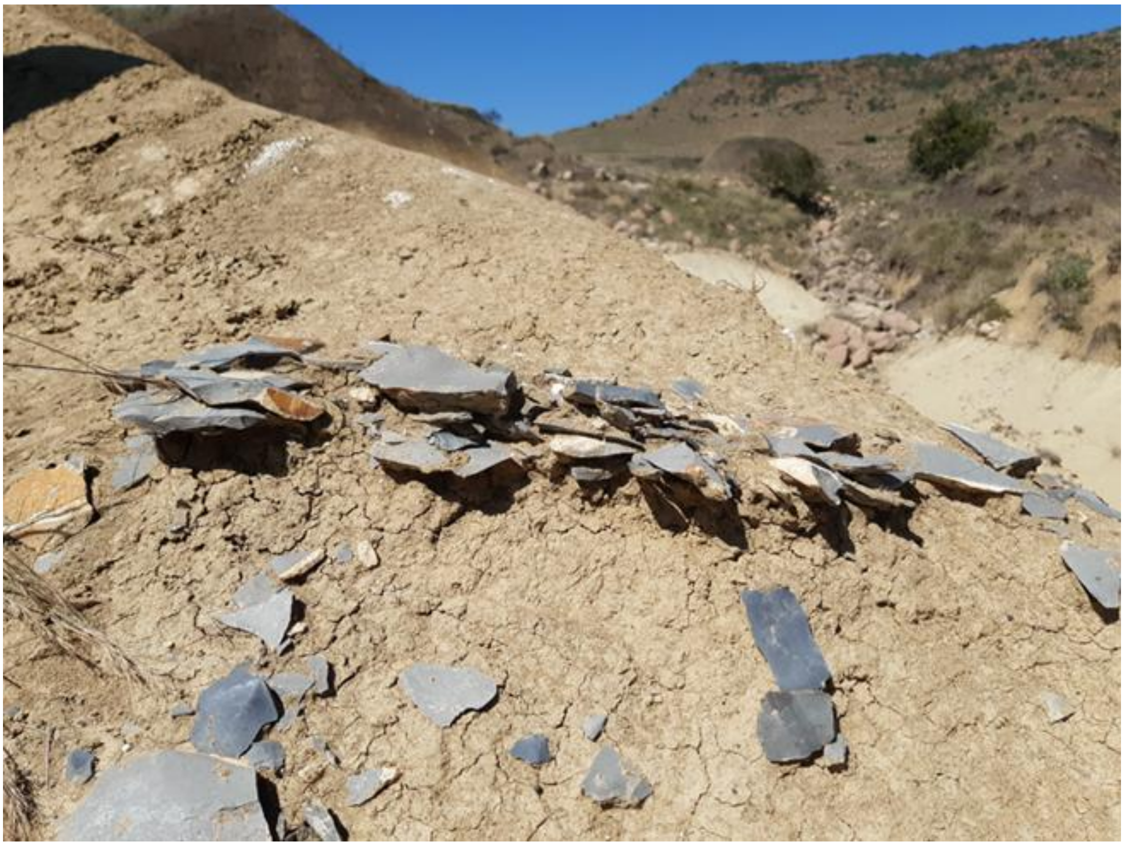

Supplementary Figure 14. Close-up on Jojosi 5 Lens 1 before excavation in 2023. Note that the artefacts lie on top of each other in several stacks, are all in a fresh state, and include small debitage.

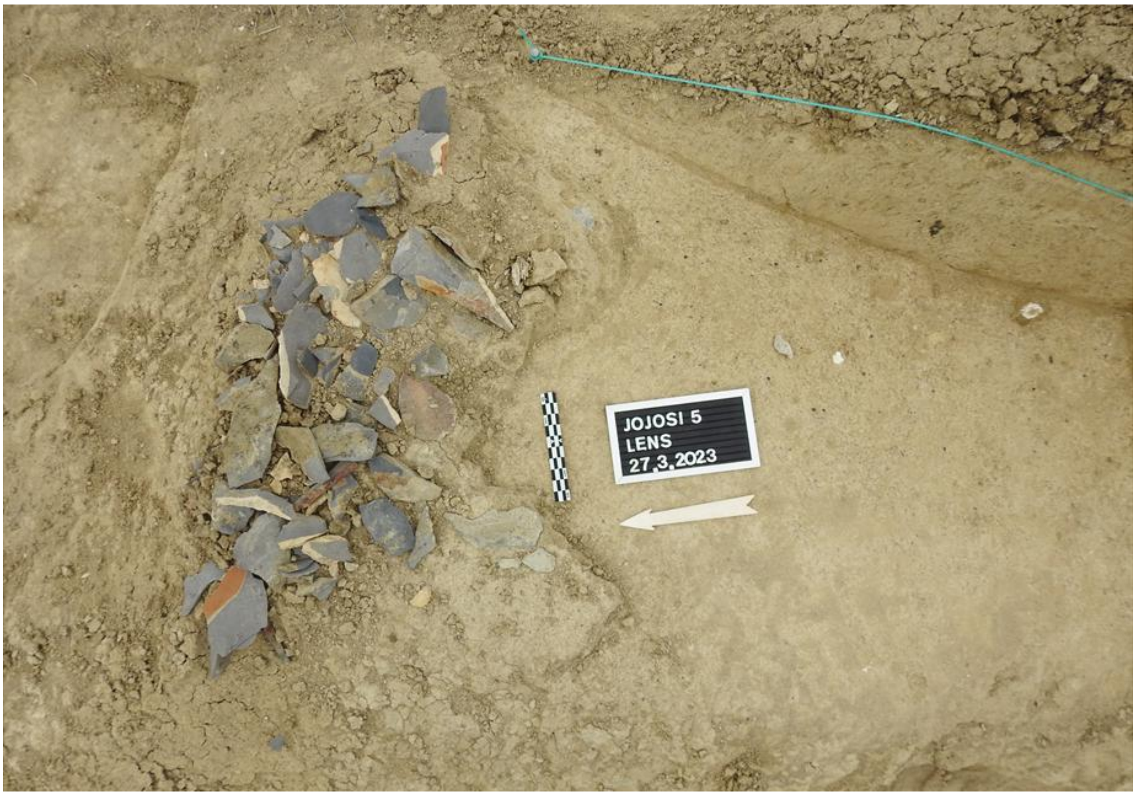

Supplementary Figure 15. Fully exposed Jojosi 5 Lens 1 in plan view. Notice the tight cluster of the artefact and the lack of further artefacts on the same elevation right next to the artefact lens.

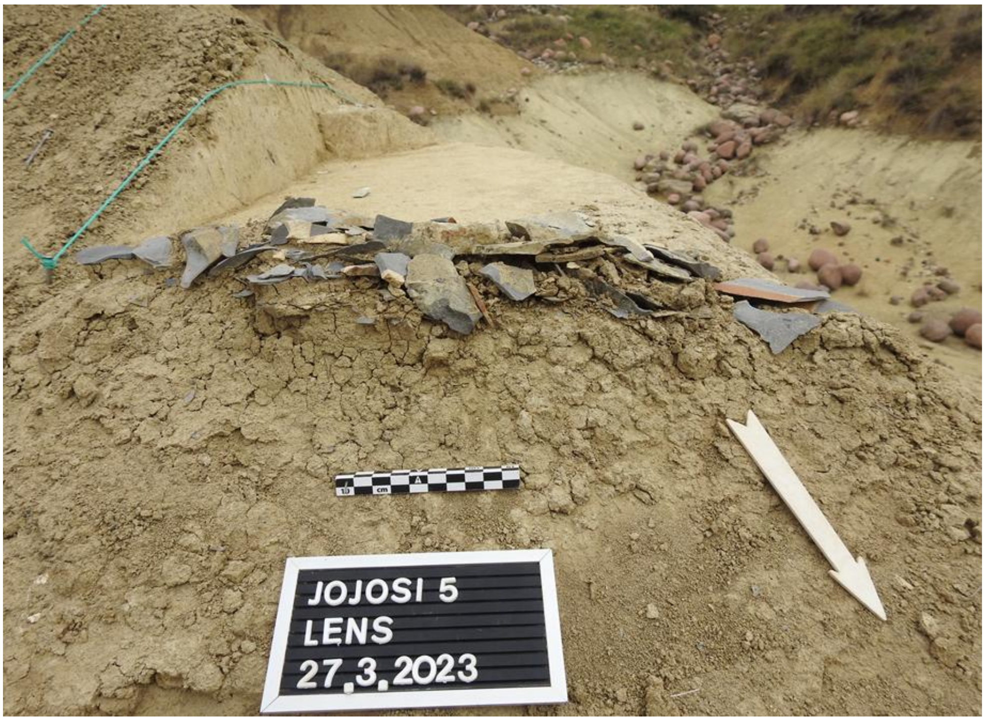

Supplementary Figure 16. Fully exposed Jojosi 5 Lens 1 in section view. Notice the multiple layers of stacked, fresh hornfels artefacts on top of each other (including small and micro debitage) and the lack of further artefacts on the same elevation right next to the artefact lens.

The sediments of the Overburden were almost devoid of any stone tools and other larger rocks and lacked faunal remains (see Supplementary Table 8). After removing a total of ca. 0.5 m³ of Overburden, we carefully uncovered the surface of Lens 1, which was only left in situ about 20 cm into the sediments (Supplementary Figures 14-16). We 3D-plotted all finds from this lens, sieved the sediments to recover small artefacts, and then dug 2 cm below the lens to fully clarify its spatial extent in three dimensions. The lens was about 6-8 cm thick and consisted exclusively of stone artefacts made on hornfels. A total of 153 pieces >2 cm were measured, with a large number of small artefacts (n=954 pieces; <20 mm). We noticed a lack of cores and tools, with all artefacts being blanks. A large proportion of the artefacts bear traces of cortex and appear to be from the initial stages of reduction and core rejuvenation.

We then continued excavating to the south and south-east into the Overburden. After ca. 70 cm without any finds, we hit another lens (‘Lens 2’) that was barely visible in the profiles. This lens is ~60 cm in diameter and was almost fully preserved and not partially eroded like Lens 1 (Supplementary Figure 17). The lens consists of a total of n=71 measured finds and a mix of cores, flakes, and one potential hammerstone (Supplementary Figure 18). Again, we encountered very high numbers of small artefacts (n=711 <2 cm) in the lens but not in the surrounding sediments. All stone tools are made of hornfels. We continued to excavate two more 25 cm transects further south-east of Lens 2, but then decided to stop excavations as no further artefacts or lenses were discovered (Supplementary Figure 19). On top, as the sediments get thicker in this direction, excavation became more and more laborious. In sum, the density of stone tools from the Overburden (n/m³=670) is several magnitudes lower compared to Lens 1 (n/m³=276,750) and Lens 2 (n/m³=223,428), suggesting clear spatial integrity of the original deposition of the stone knapping events and little to no size-sorting or post-depositional movement of artefacts (see Supplementary Table 8). An overview of the spatial distribution of finds in both lenses can be seen in Supplementary Figures 22 & 23.

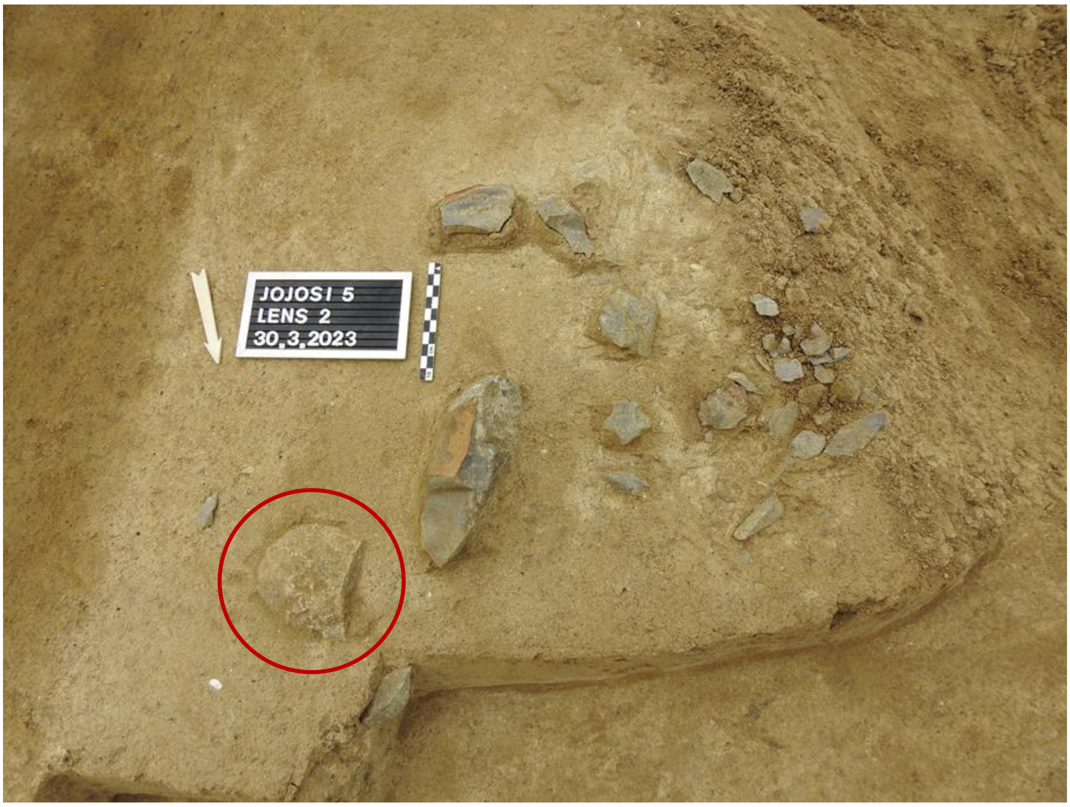

Supplementary Figure 17. Fully exposed Jojosi 5 Lens 2 in plan view. Note the tight clustering as well as the existence of flakes and cores, and a hammerstone (red circle) on the bottom left.

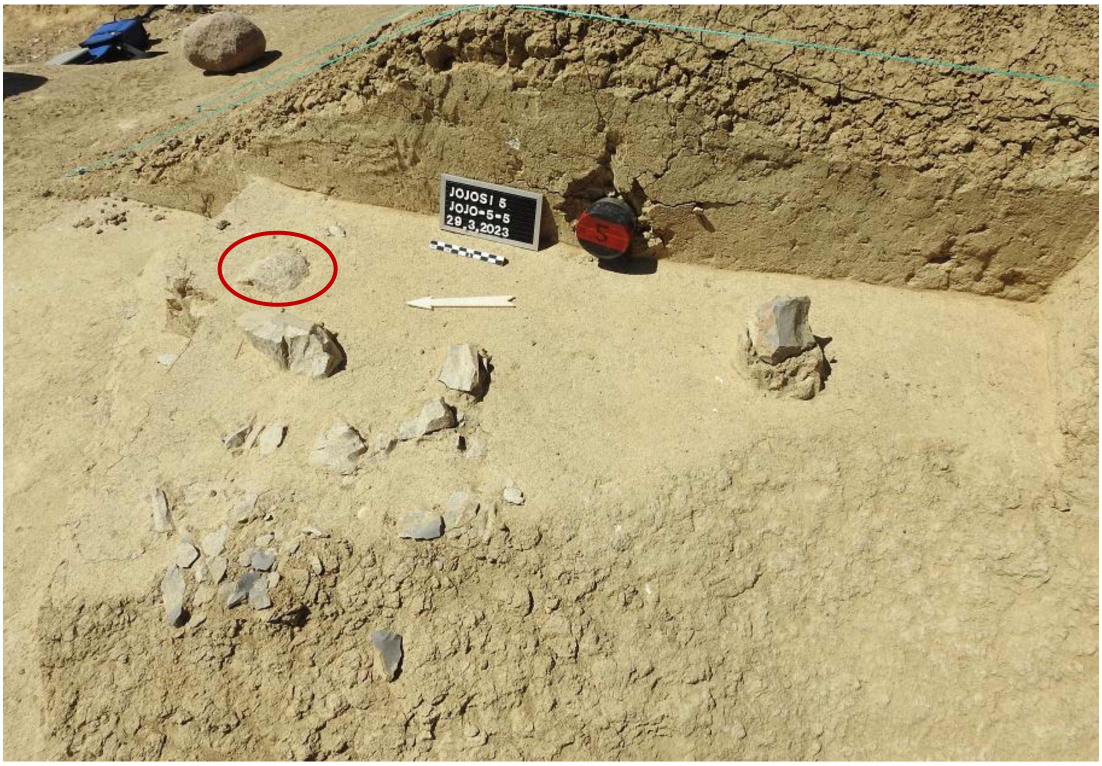

Supplementary Figure 18. Fully exposed Jojosi 5 Lens 2. Note the existence of flakes, cores, and a hammerstone (red circle) on the top left. OSL sample Jojosi-5-5 is seen in the wall of Overburden behind.

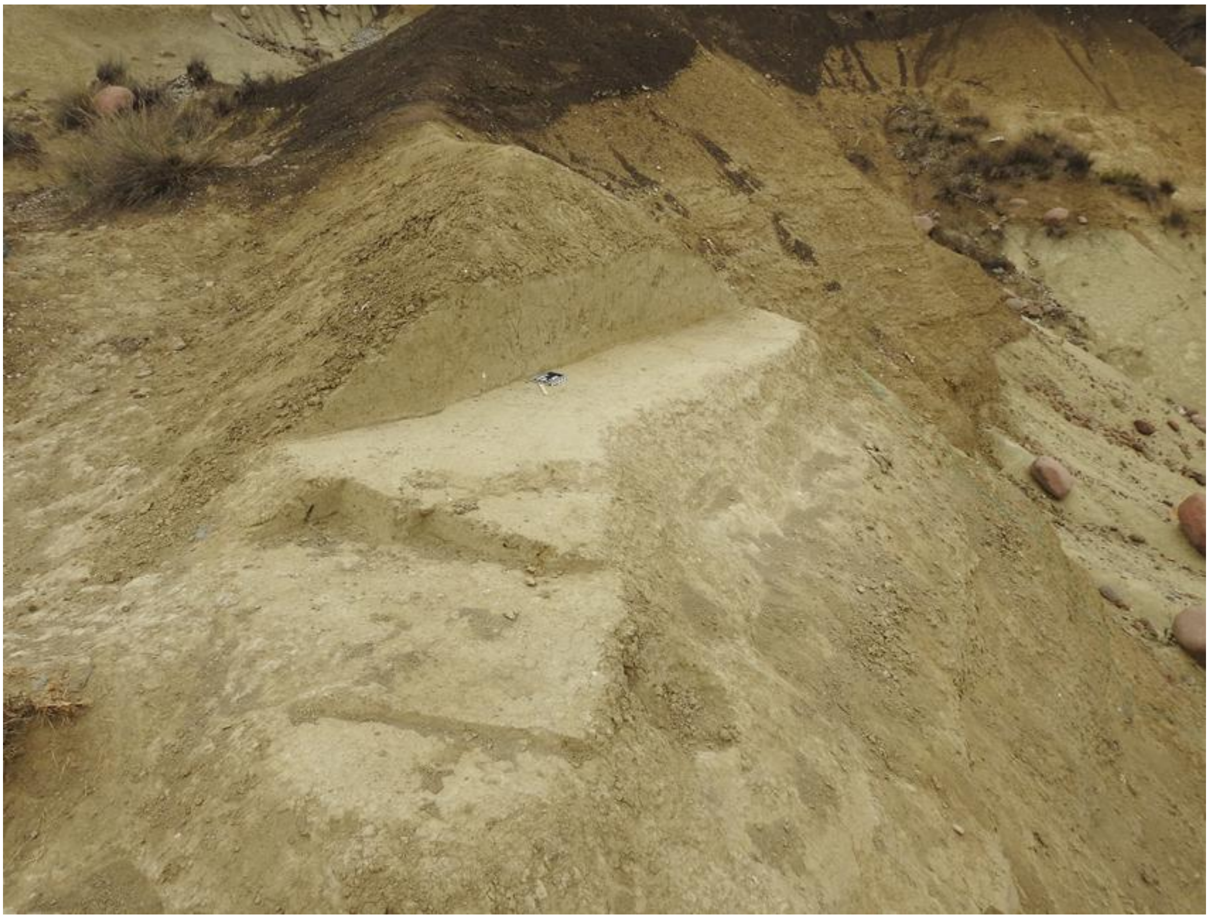

Supplementary Figure 19. Final state of continued excavation at Jojosi 5 in 2023. Note the lack of any additional artefact scatters or concentrations encountered after the artefacts from the lenses were removed.

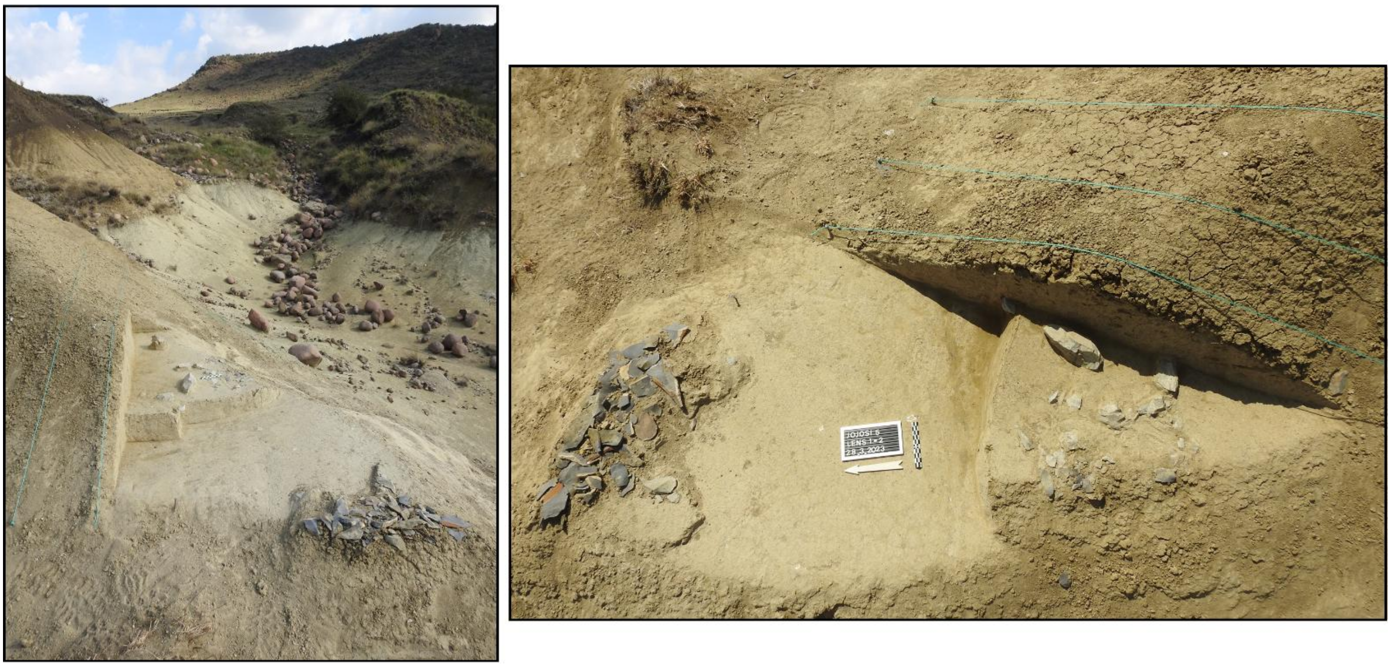

Supplementary Figure 20. Fully exposed Lens 1 (foreground) and Lens 2 (background) of Jojosi 5.

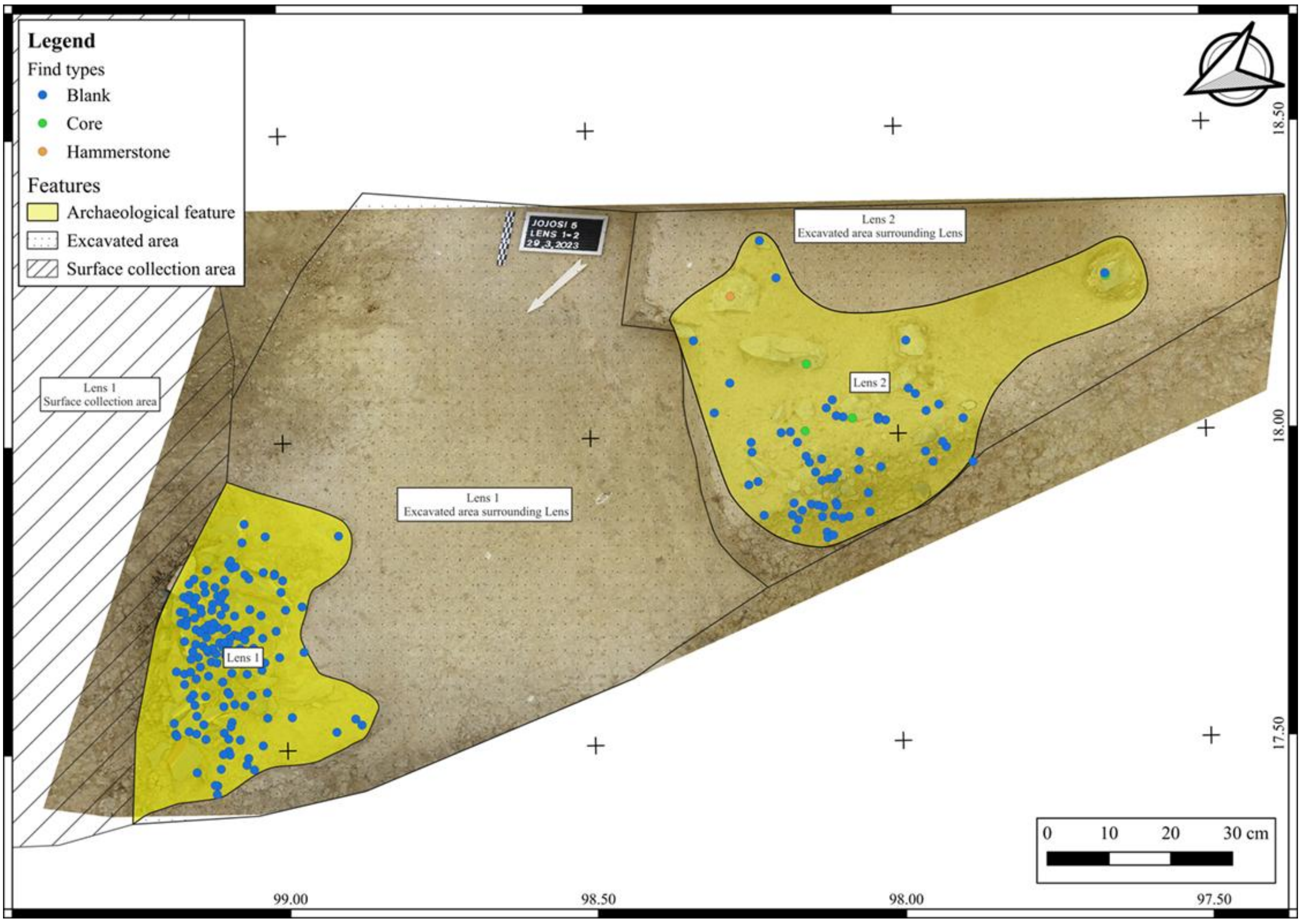

Supplementary Figure 21. SFM of the fully exposed Jojosi 5 Lens 1 and Lens 2 with indication of the distribution of 3D measured finds. Note the tight spatial demarcation and the absence of intervening finds between the lenses.

Jojosi 6

After finishing our work at Jojosi 5 in 2023, we started excavating a lens spotted in the profile, ca. 15 m southeast of this site, which we call Jojosi 6 (Supplementary Figures 22 & 23). Here, excavations followed a slightly different approach due to the different geometry of the sediments. We established a total of eight 25 cm transects along a stable Y-line with the Total Station and then proceeded to dig approximately 15 cm deep within these transects into the slope. The sediments were again of the same consistency, matrix, and colour as in Jojosi 5, belonging to the same sediment body Layer 4 (see Supplementary Information 1 for detailed description). We again excavated in contexts, starting with Overburden into Lens and continuing slightly below it. After ca. 10 cm, we discovered two separate lenses of stone tools (Lens 1 and Lens 2) at Jojosi 6. Some of Lens 1 was already eroding down the slope (Supplementary Figure 24), but Lens 2 was fully intact. Like at Jojosi 5, we first collected all surface finds regardless of size, also scraping the 2 cm of the surface and sieving the resulting sediments. We were even able to refit some of the surface finds, indicating that the artefacts have only recently eroded out of an *in situ* context (Supplementary Figure 25).

The remains of Jojosi 6 Lens 1 are approximately 60 cm in diameter (Supplementary Figure 26), ~6 cm thick, and rich in stone tools (Figure 27). In total, we collected a total of n=7881 finds from screened sediments and measured n=306 lithics >2cm in situ (Supplementary Table 7). All artefacts, regardless of size, are on hornfels. No fauna was preserved. Lens 2 of Jojosi 6 is approximately 40 cm separate from Lens 1 (Supplementary Figure 26). This lens is ca. 5-

6 cm thick and also rich in stone tools. In total, we recovered n=414 lithics >2 cm *in situ* (Supplementary Table 7) and collected a total of n=6719 finds from screened sediments <2 cm during the campaigns in 2023 and 2024. All artefacts are on hornfels. At both lenses, only blanks and no cores or tools were recovered. No fauna was preserved. We could not finish excavating all of Lens 2 within the field season and left the remainder encased in the sediments to be excavated in 2024. The 'Overburden' sediments above and between the lenses have many magnitudes of artefacts fewer (n/m³=1,659), compared to the densities of Lens 1 (n/m³=909,677) and Lens 2 (n/m³=2,377,667), again suggesting that the lenses have discrete spatial structure and integrity, similar to the situation of Jojosi 5 (Supplementary Table 8).

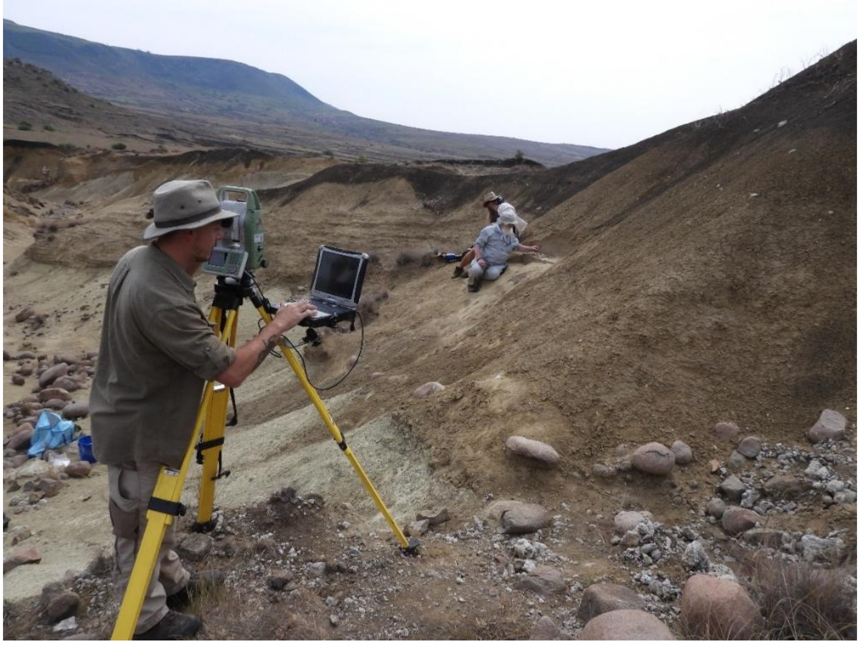

Supplementary Figure 22. Excavation in progress at Jojosi 6 in 2024. Individual finds >2 cm are measured in 3D with a total station and an EDM system, here by G. Möller. A. Mazel and M. Blessing are excavating.

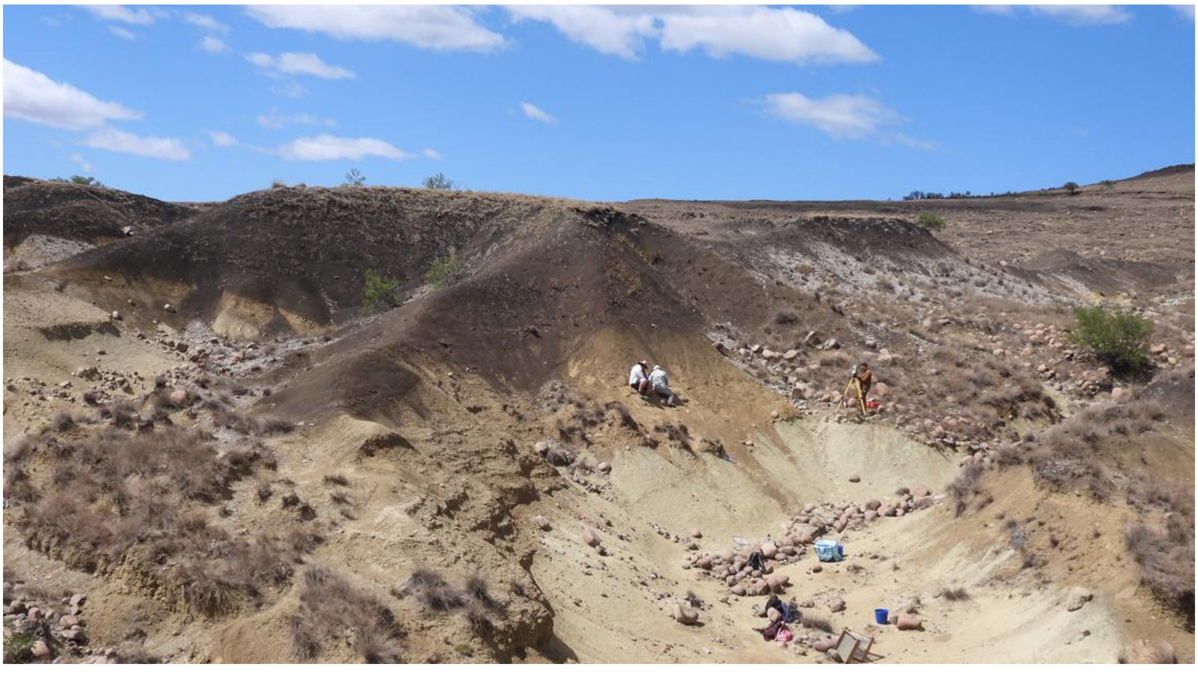

Supplementary Figure 23. Panoramic view of the site of Jojosi 6 in 2024, showing its context within the donga system.

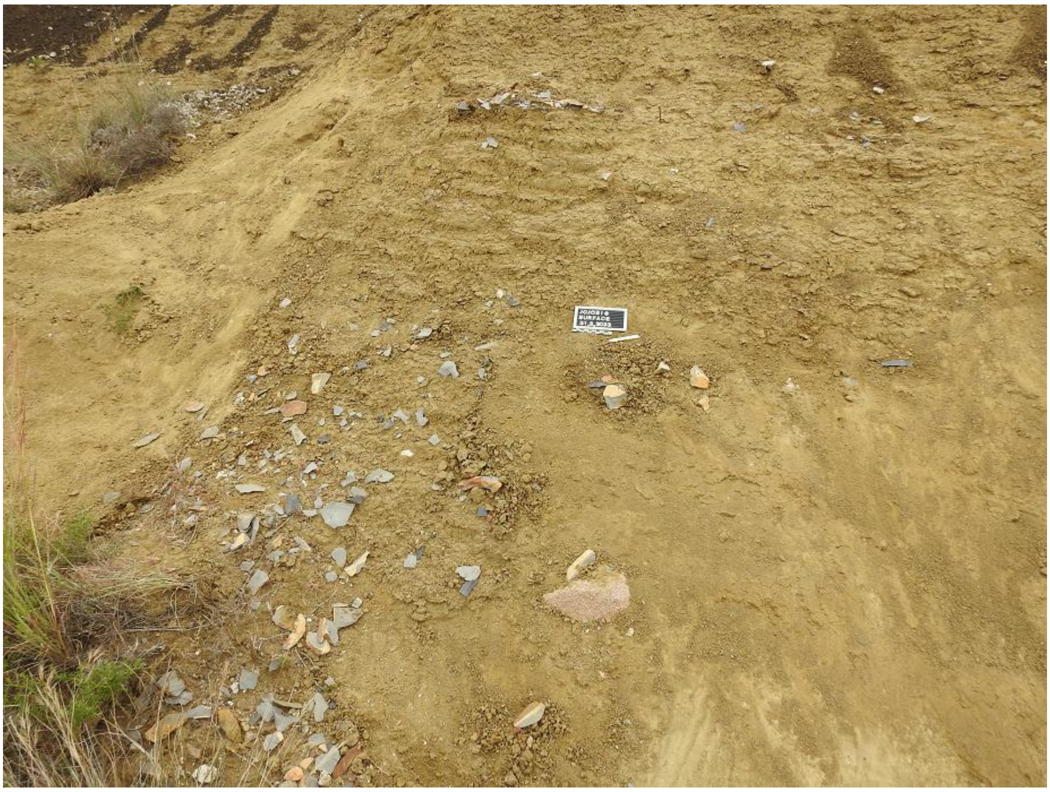

Supplementary Figure 24. View on Jojosi 6 before excavation. Note the *in situ* lens within the slope at the top of the picture, visible as a lateral line, and the scatter of surface artefacts below.

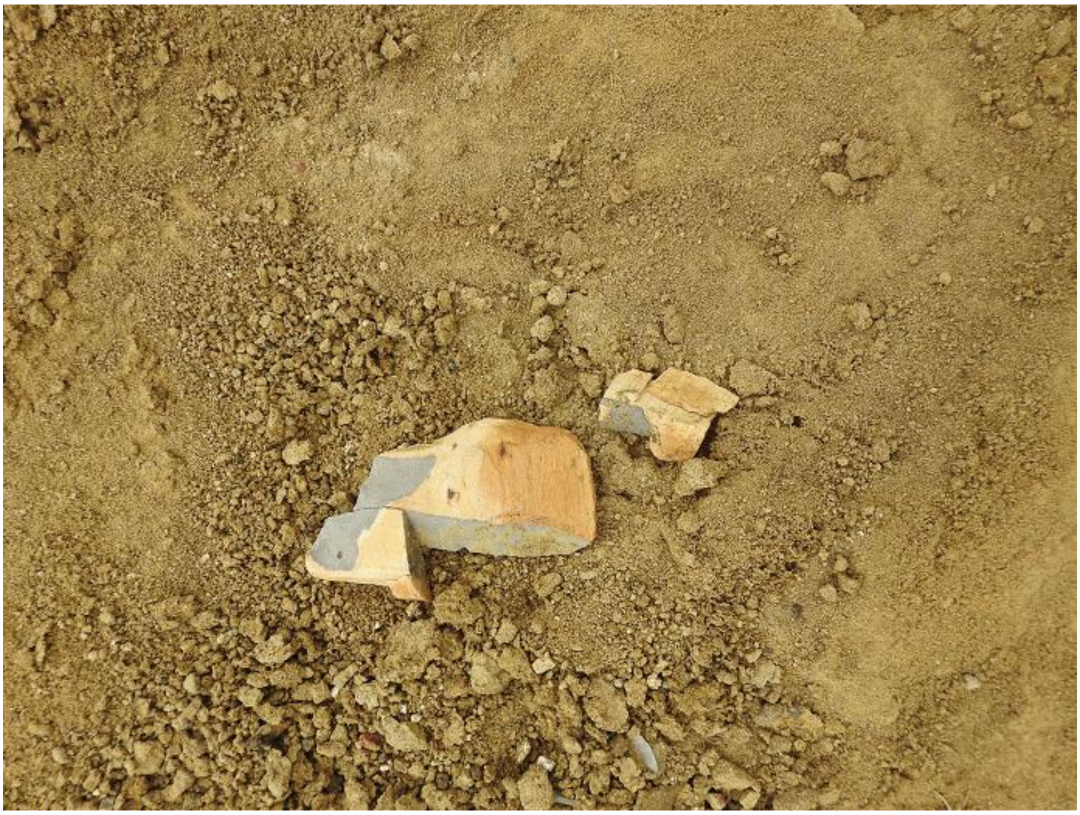

Supplementary Figure 25. Refit set of two cortical hornfels flakes at Jojosi 6 from the Surface context before excavation.

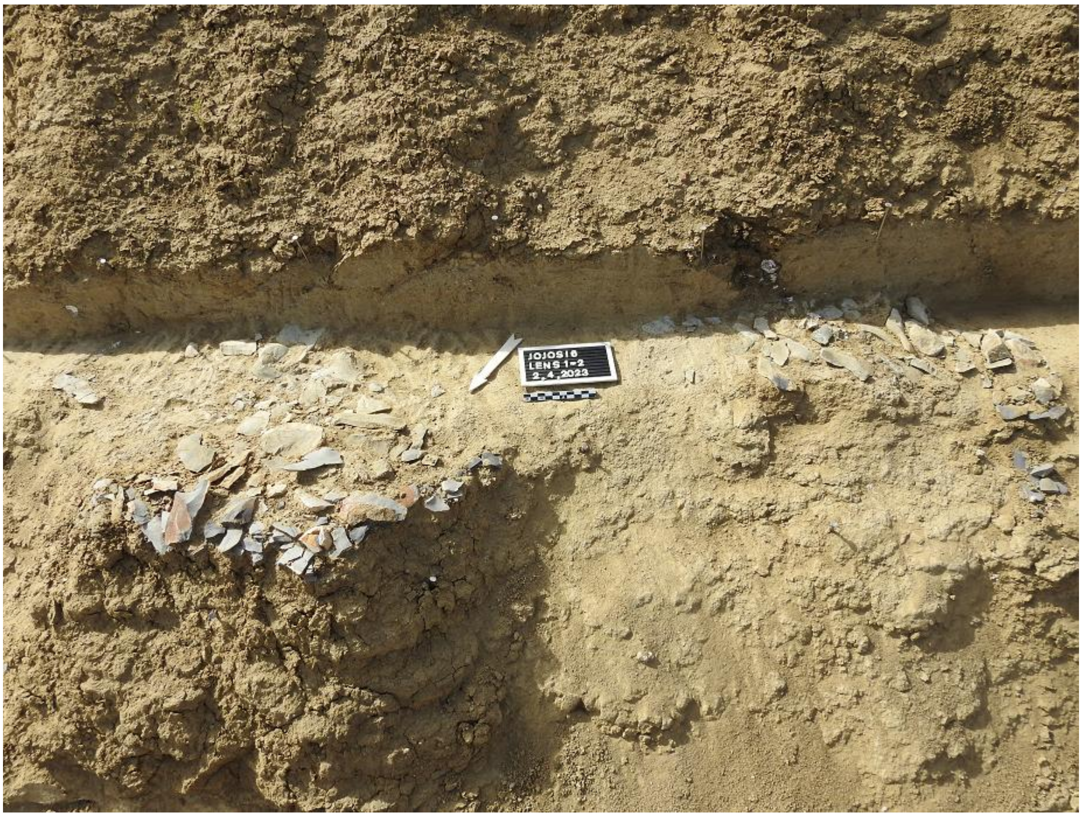

Supplementary Figure 26. Fully exposed Jojosi 6 Lens 1 (left) and partially uncovered Lens 2 (right) 6 in 2023. Note the gap between the lenses devoid of artefacts, and that Lens 2 still continues into the overhang sediments to the south.

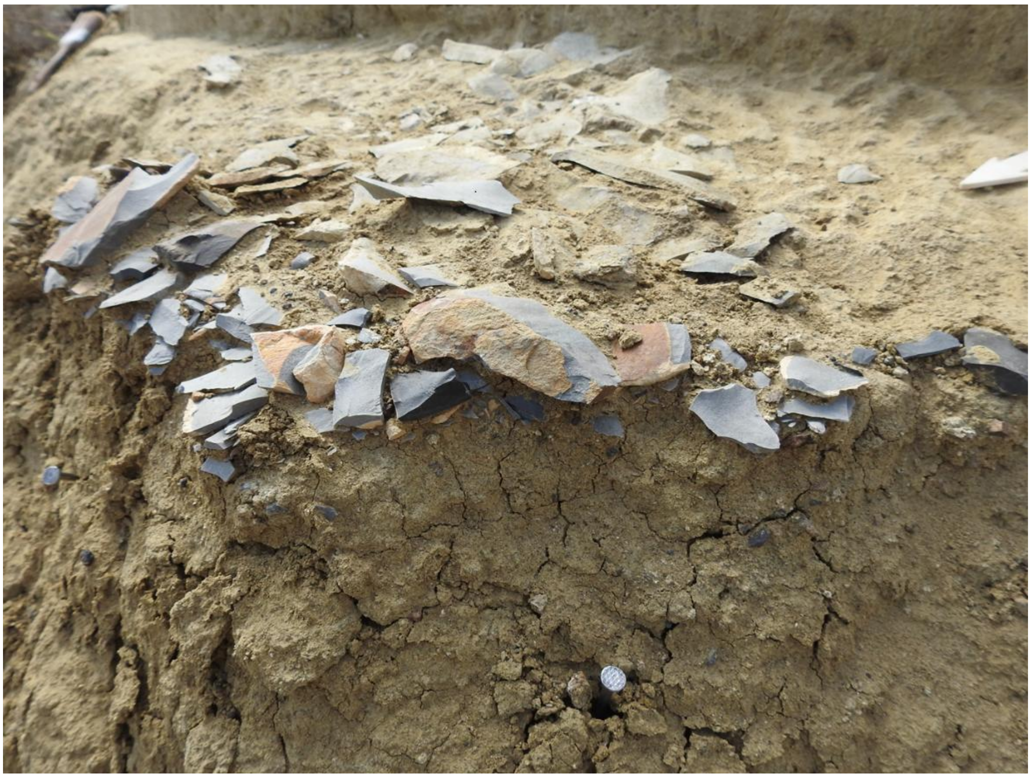

Supplementary Figure 27. Close-up of Jojosi 6 Lens 1. Note the stacked nature of the lithic artefact concentration, the fresh and homogenous nature of the artefacts, and the abundant small debitage visible.

In 2024, we continued the excavations of Jojosi 6 Lens 2 with the same methods and uncovered the entire artefact concentration. We quickly reached the remainder of Lens 2, which was approximately 50 cm x 30 cm large and again featured very high amounts of large and small lithic finds (Supplementary Figure 28).

Combining results from 2023 and 2024, Lens 2 of Jojosi 6 is approximately 60 cm in diameter (Supplementary Figure 29). It is ca. 6 cm thick and very rich in stone tools. All artefacts, regardless of size, are on hornfels. The large number of micro-artefacts is particularly noteworthy. No fauna was preserved. Interestingly, only blanks and no cores or tools were recovered from Jojosi 6, Lens 2. A large proportion of the artefacts bear traces of cortex and appear to be from the initial stages of reduction and core rejuvenation.

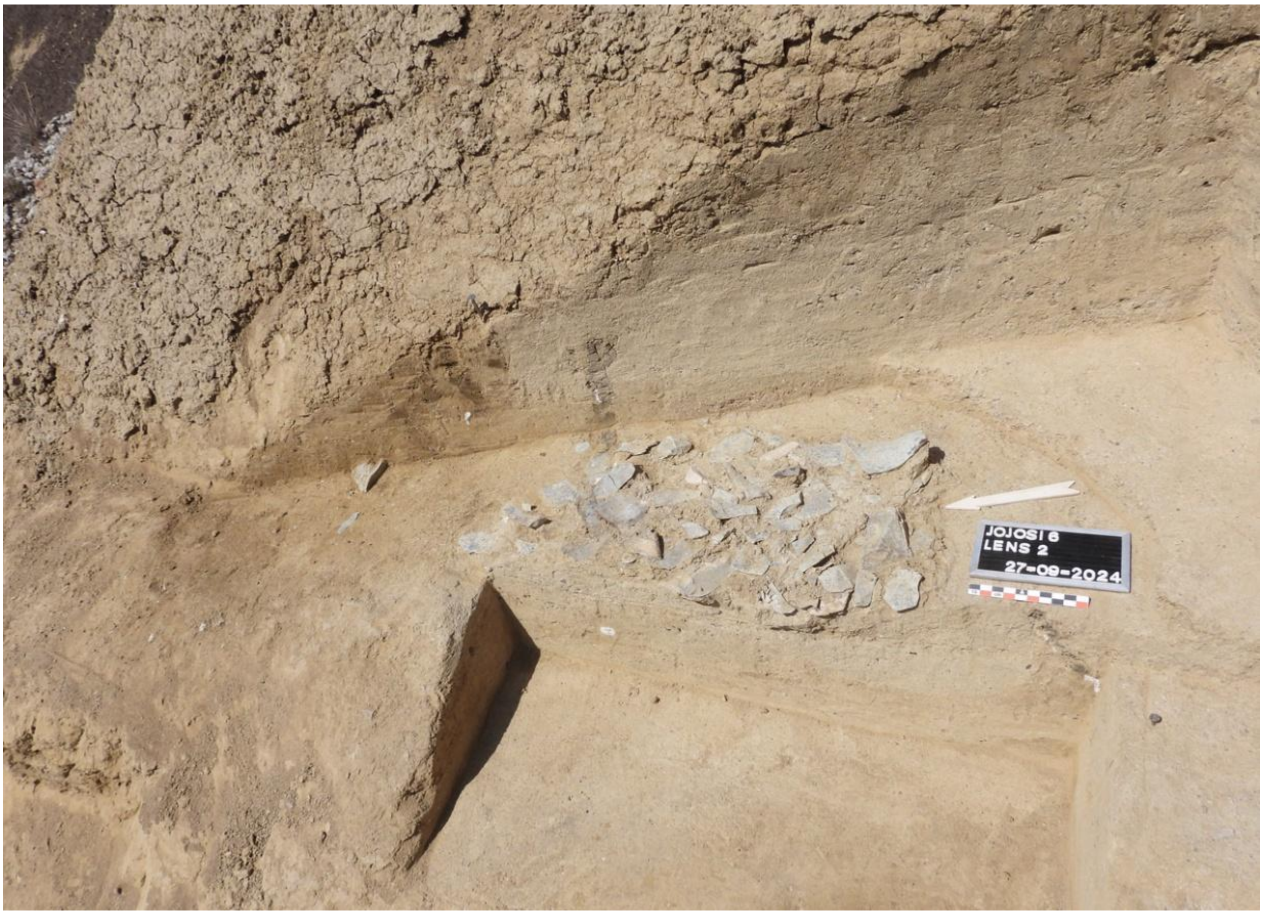

Supplementary Figure 28. The extent of the remaining Lens 2 of Jojosi 6 after exposure in 2024 (the original lens went further to the front of the picture, which was excavated already in 2023). Note the high density of small and large hornfels artefacts and the tight spatial clustering.

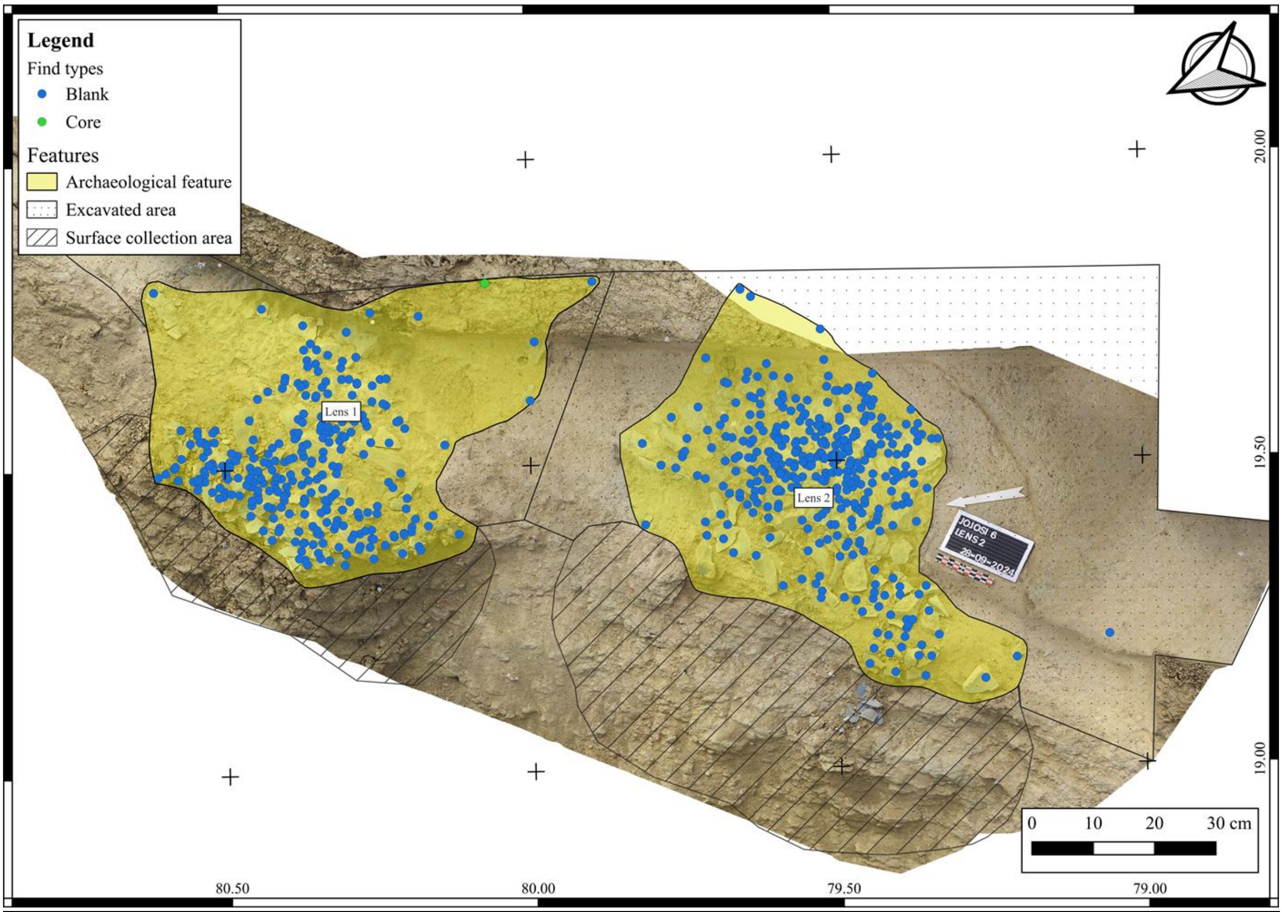

Supplementary Figure 29. Synthetic of the fully exposed Jojosi 6 Lens 1 and Lens 2 with indication of the distribution of 3D measured finds. Note the tight spatial demarcation and the absence of intervening finds between the lenses.

Jojosi 7

Jojosi 7 was identified as an actively eroding hornfels concentration in a sediment body only ca. 20 m north-north-east from Jojosi 1, the main site originally dug by A. Mazel in 1991 (Supplementary Figure 30). The main difference between Jojosi 7 to the other excavated sites consists of a much larger modern brownish soil on top of the artefact-bearing sediments (Supplementary Figure 31). These sloping sediments in the landscape are actively eroding, making it an urgent objective for excavation. At Jojosi 7, the artefact lenses are also much closer to the underlying colluvium: approximately 20 cm separates this colluvium line from the stone tool horizon (Supplementary Figure 32). Similar to the other Jojosi sites, however, only fresh, grey hornfels artefacts without any other raw materials crop out of the profile walls.

On October 2^{nd,} 2024, after finishing Jojosi 6, we started work in Jojosi 7. First, we opened a 1.5 m wide trench and removed the ca. 0.5 m thick find-empty, dark-brown, loamy and wet sediment on top of the artefact lens (Supplementary Figures 32-34). Due to the wet status of these sediments, we could not systematically screen the Overburden context. That being said, no large or small artefacts were encountered during digging or by hand inspection of excavated sediments. See Supplementary Info 1 for a more detailed description of the sediments and their interpretation at Jojosi 7. Again, we stopped removing the Overburden context once reaching the first hornfels stone artefacts. We then carefully excavated this concentration as context Lens 1 with trowels. These sediments were still clayey, baked, and wet from post-depositional changes of the original sediments (e.g., including root channels), hindering

systematic dry-screening (Supplementary Information 1 for further information). Instead, we carefully hand-picked through all sediments to recover small finds <20 mm to the extent possible. The other methods of excavations and the 3D measuring of finds >2 cm *in situ* remained like the other Jojosi sites. Due to the much larger size of the artefact concentration than initially expected (see below), we were not able to finish excavating the entire site in 2024.

A remarkable feature of Jojosi 7 is the larger extent of the artefact concentration (Supplementary Figure 36, over 100 cm and ongoing and a couple of centimetres thick), a much better preservation of bone and teeth (Supplementary Figure 35), and indications of potential burning on bone and sediment. In total, we measured 499 stone tools >2 cm, 22 bones, and 7 teeth >2 cm in situ. The lithics at Jojosi 7 mostly consist of blanks but also feature some tools and cores. The bone and teeth are mostly fragmented and exhibit a whitish to black (inner) colour. The number of small stone artefacts <20 cm laboriously hand-picked from the wet sediments goes into the many hundreds (n=3127; Supplementary Table 7).

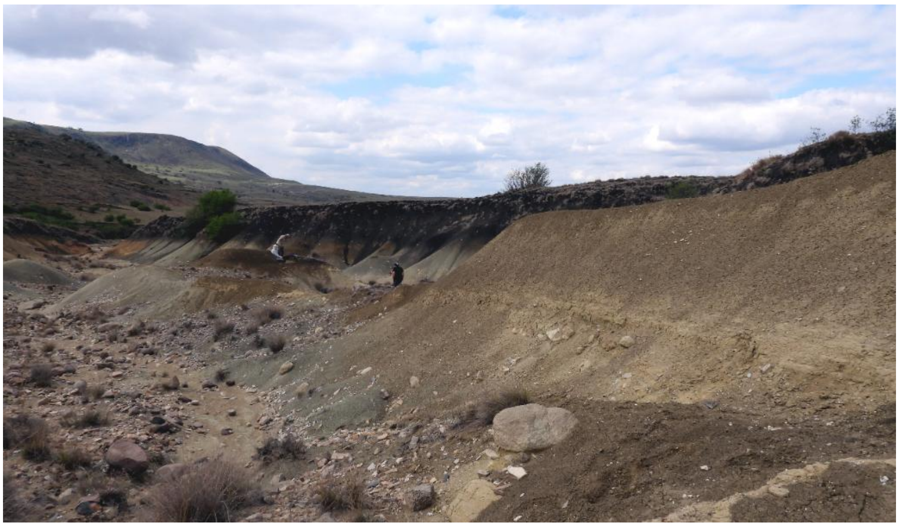

Supplementary Figure 30. View on Jojosi 7 during excavation (left side with people) and its proximity to the original Jojosi 1 excavations in 1991 by A. Mazel (sediment body to the right of the picture).

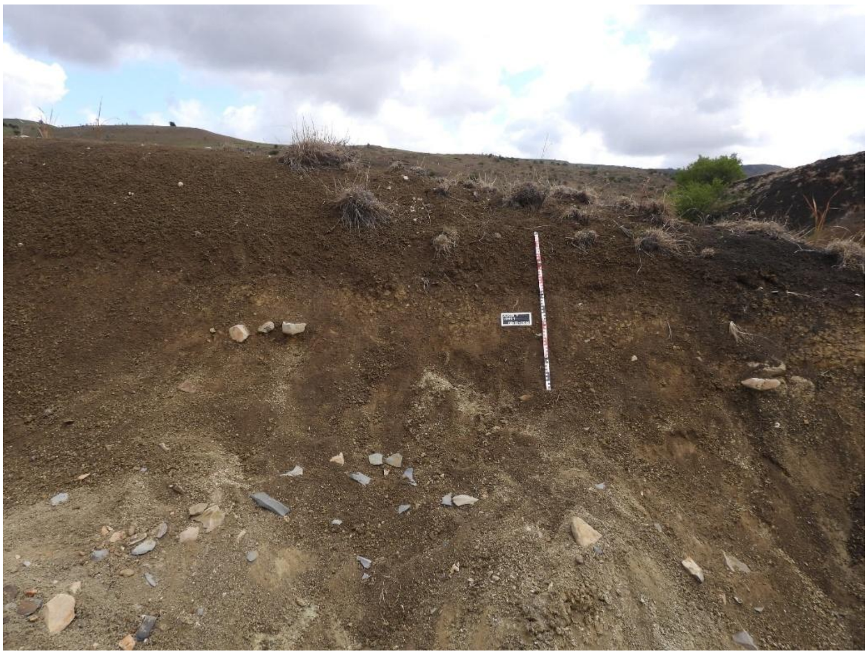

Supplementary Figure 31. Jojosi 7 before starting the excavations. Note the thick overburden of dark-brown soil.

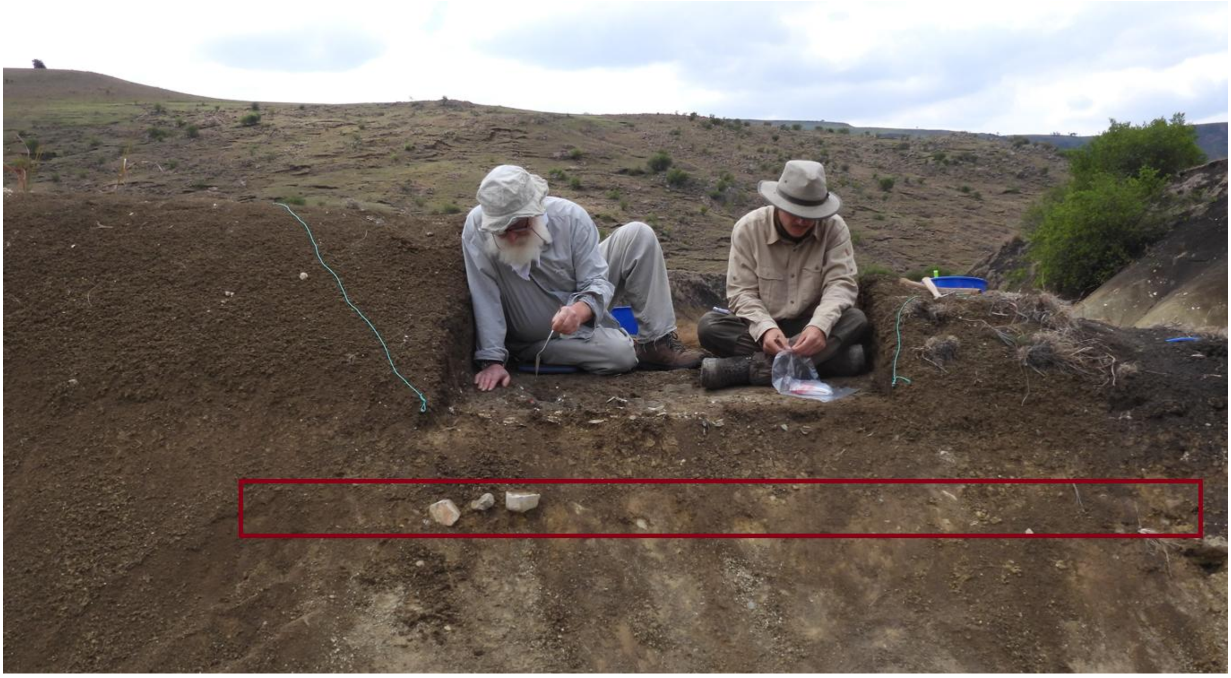

Supplementary Figure 32. Jojosi 7 during excavation by A. Mazel and G. Möller, showing the short distance between the pebble line (red box; contact between rock Unit 1 and 4) and the artefact lens that is exposed on the surface of the excavation.

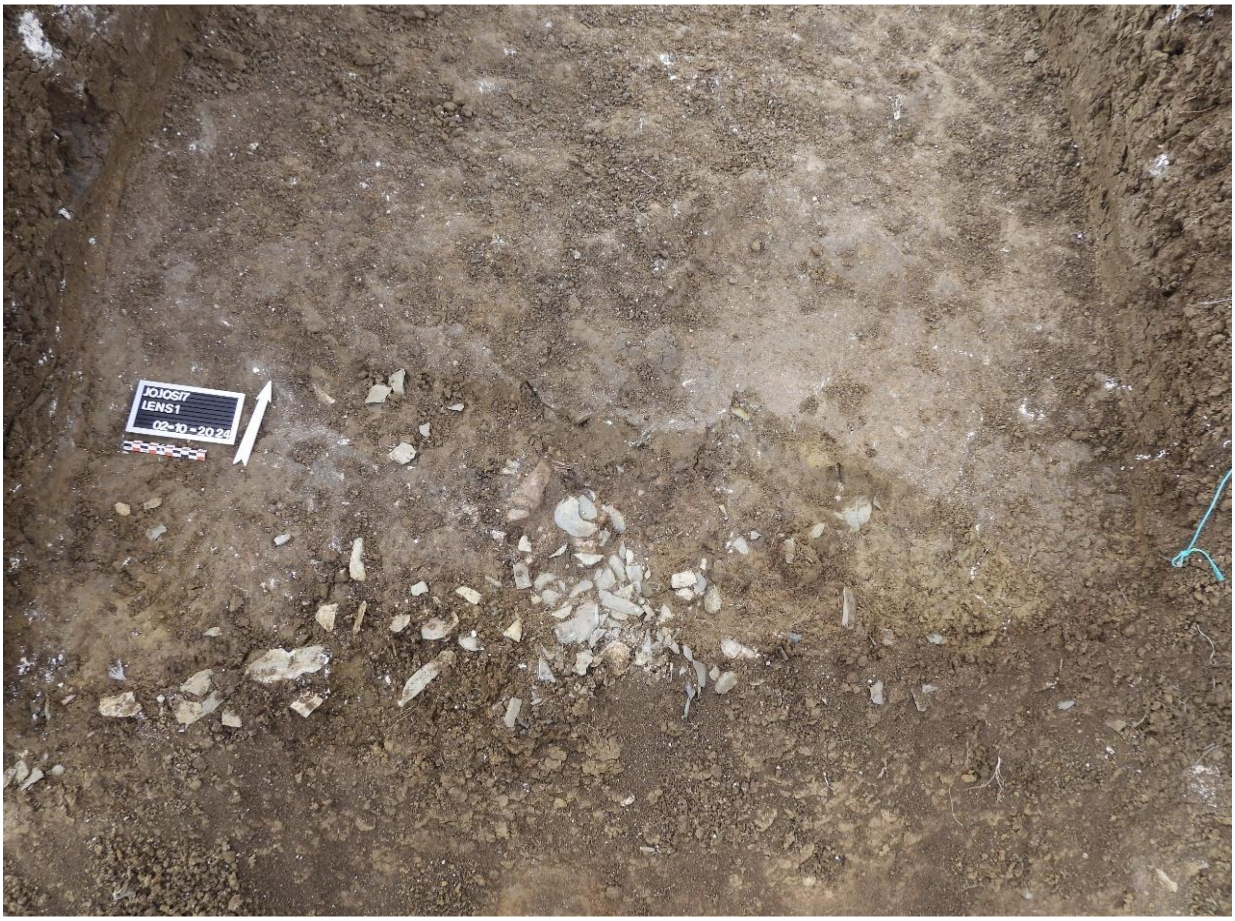

Supplementary Figure 33. View on the uppermost surface of the exposed artefact lens at Jojosi 7 on the first day of excavations. Note the wet, clayey sediments and the tight concentration of hornfels artefacts.

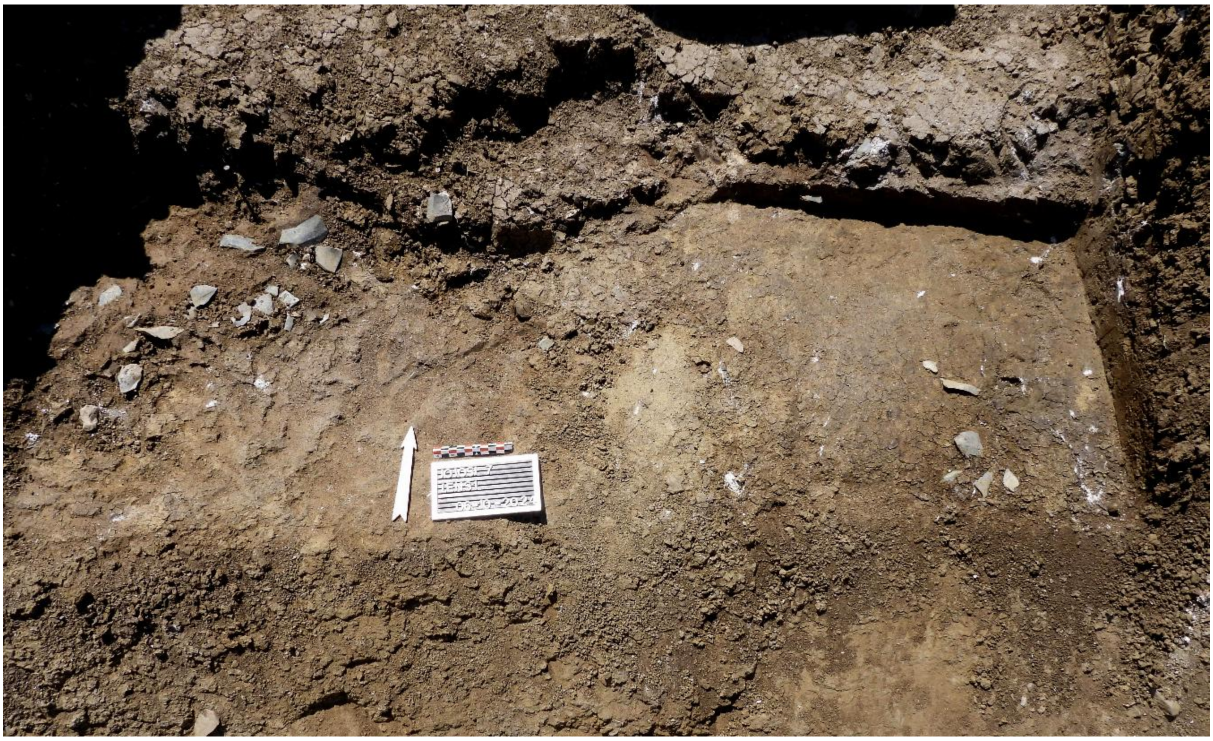

Supplementary Figure 34. Distribution of hornfels artefacts of Jojosi 7 Lens 1 after removing the central concentration of lithics. Note the wide dispersion of artefacts over a single surface of well over 1 m.

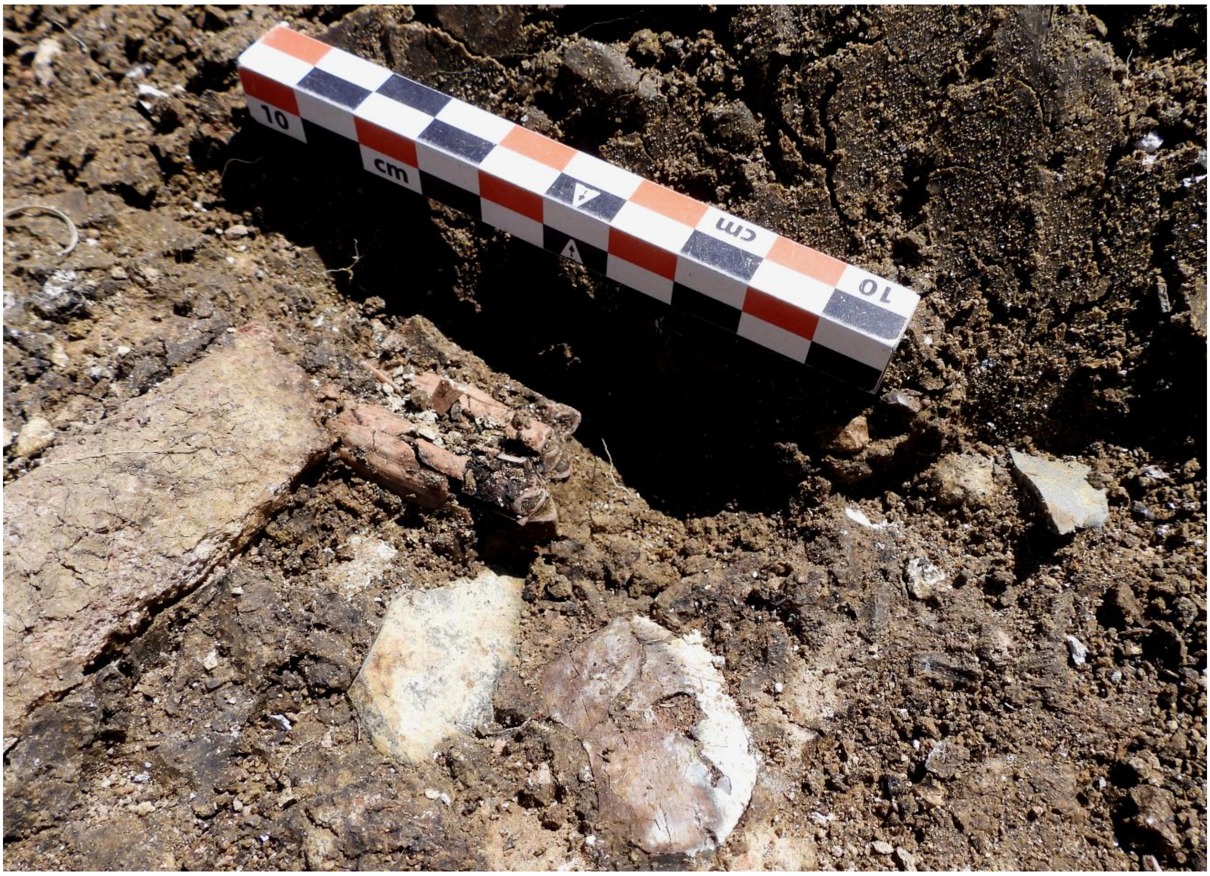

Supplementary Figure 35. Example of a tooth and bone preserved in association with hornfels artefacts in Jojosi 7.

Supplementary Discussion 3. Methods of the old excavations and legacy collections at Jojosi 1

The first written reports recognising the archaeological potential of the Jojosi Dongas originate from expeditions conducted by Lebzelter and Schmidt, who report Stone Age artefacts in the region. A synthesis of the earlier research history can be found elsewhere[26]. In the context of geological research in the area by Botha[2], he and A. Mazel recognised the high archaeological potential of the area and saw the threat of gully expansion to the intact archaeological sites interbedded with the colluvial deposits. In 1991, Mazel directed the first excavation of a stratified open-air site in KwaZulu-Natal[2]. These activities are documented by personal field notes, comprehensive photographic records, artefact counts, and excavation drawings (see[3]), which describe eroding artefact and stratified artefact concentrations such as Jojosi 1 at the side walls of the dongas (Supplementary Figures 36 & 37). Due to the combination of high artefact concentrations, the presence of large amounts of small debitage, and the spatial restriction of these in a buried lenticular feature, the site was interpreted as a potentially in situ hunter-gatherer knapping floor. Apart from the excavation of the most intact and stratified lens of Jojosi 1 (Supplementary Figures 38-40), Mazel also dug three further concentrations (Jojosi 2–4), but the extent of the documentation and the amount of material are not comparable. Apart from what is mentioned in a local newspaper (Witness Reporter, 1991), the material curated in the KwaZulu-Natal Museum (Pietermaritzburg) had not been described, analysed, or published.

In March 2023, we undertook specific foot and drone surveys assisted by old photographs and a 3D landscape model made to re-discover the original excavations at Jojosi by A. Mazel from 1991 (Jojosi 1 in particular). G. Möller was able to identify the find area, and this was confirmed by M. Will and C. Sommer (Figure 36). Unfortunately, no intact MSA artefacts are left within the slopes of the donga, even though there is a high number of fresh hornfels artefacts on the surface. We documented the current situation at the site and also took relevant luminescence samples to be able to provide an age to the old collections by A. Mazel. We also performed a detailed technological and typological study of the old MSA artefact collections of Jojosi 1 stored in the KZN Museum in Pietermaritzburg from April 10th until May 12th in 2023. These results were published in 2024 and can be found elsewhere[3].

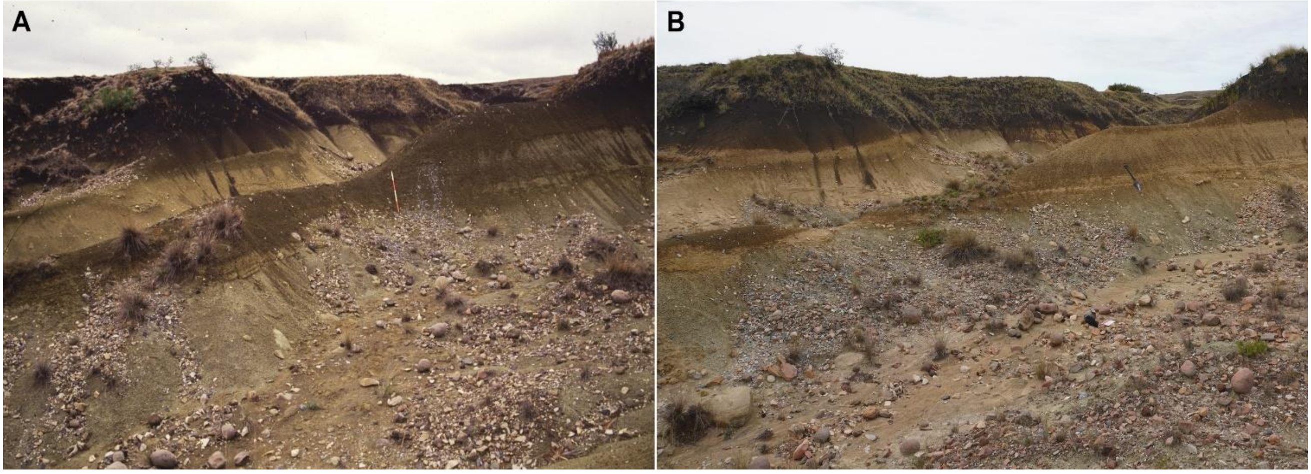

Supplementary Figure 36. Figures in 1991 of Jojosi 1 taken by A. Mazel (A) and the rediscovered site documented in March 2023 by G. Moeller (B). Note the ongoing erosion of the darker patches (topsoil) of the donga in the foreground.

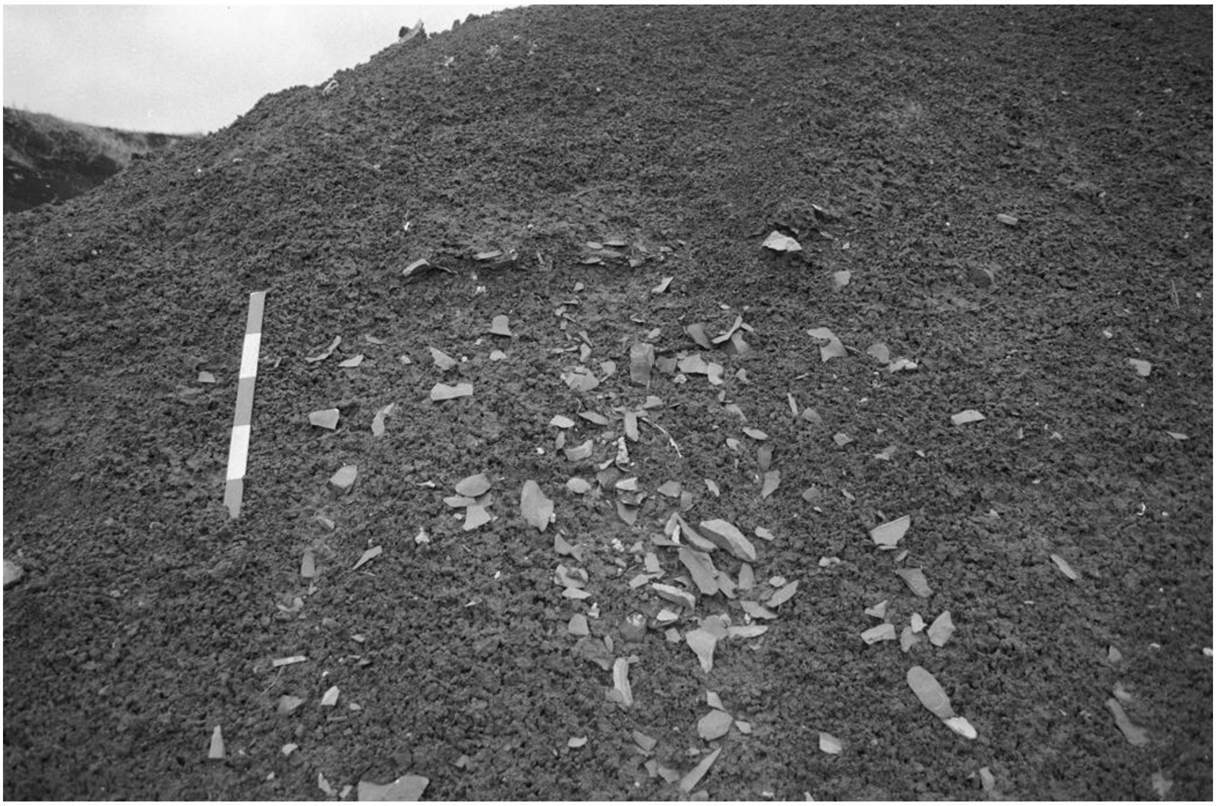

Supplementary Figure 37. Jojosi 1 before excavation in 1991. Note the richness of recently eroding artefacts on the slope and the tight concentration of in situ artefacts on top of them.

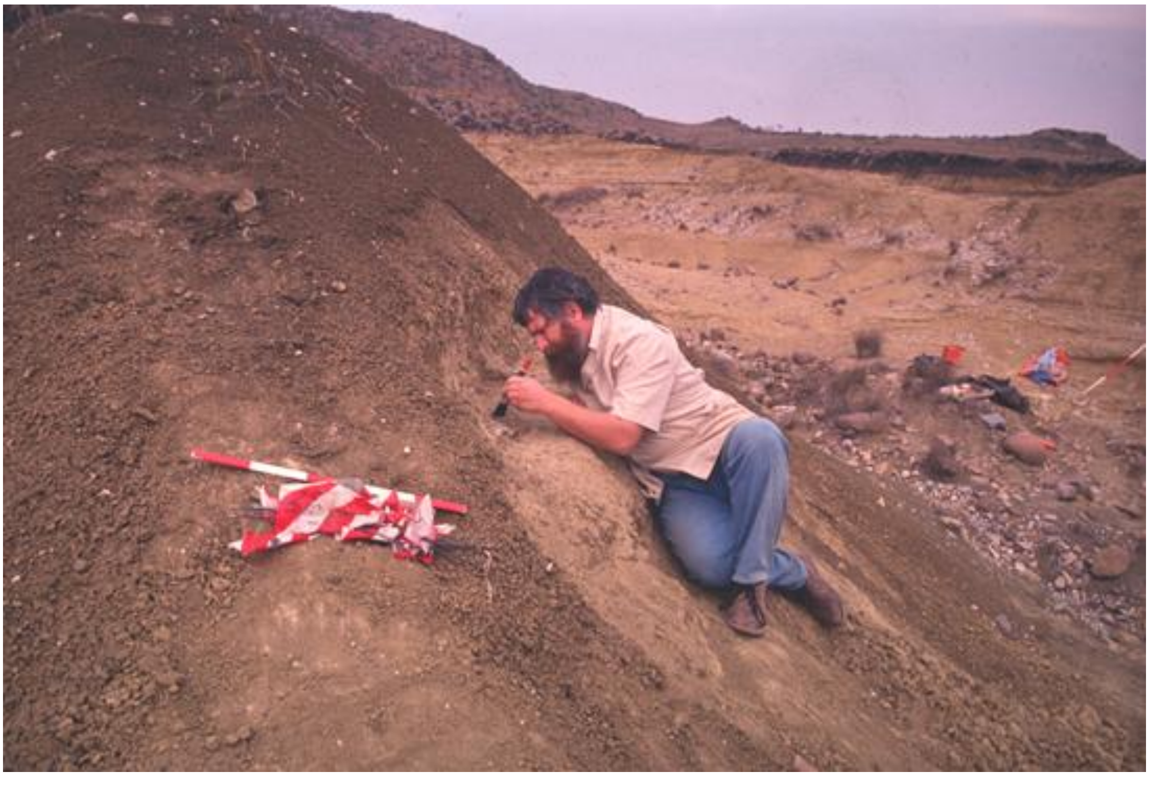

Supplementary Figure 38. A. Mazel excavating at Jojosi 1 in 1991. Note the careful uncovering of artefacts via brushes.

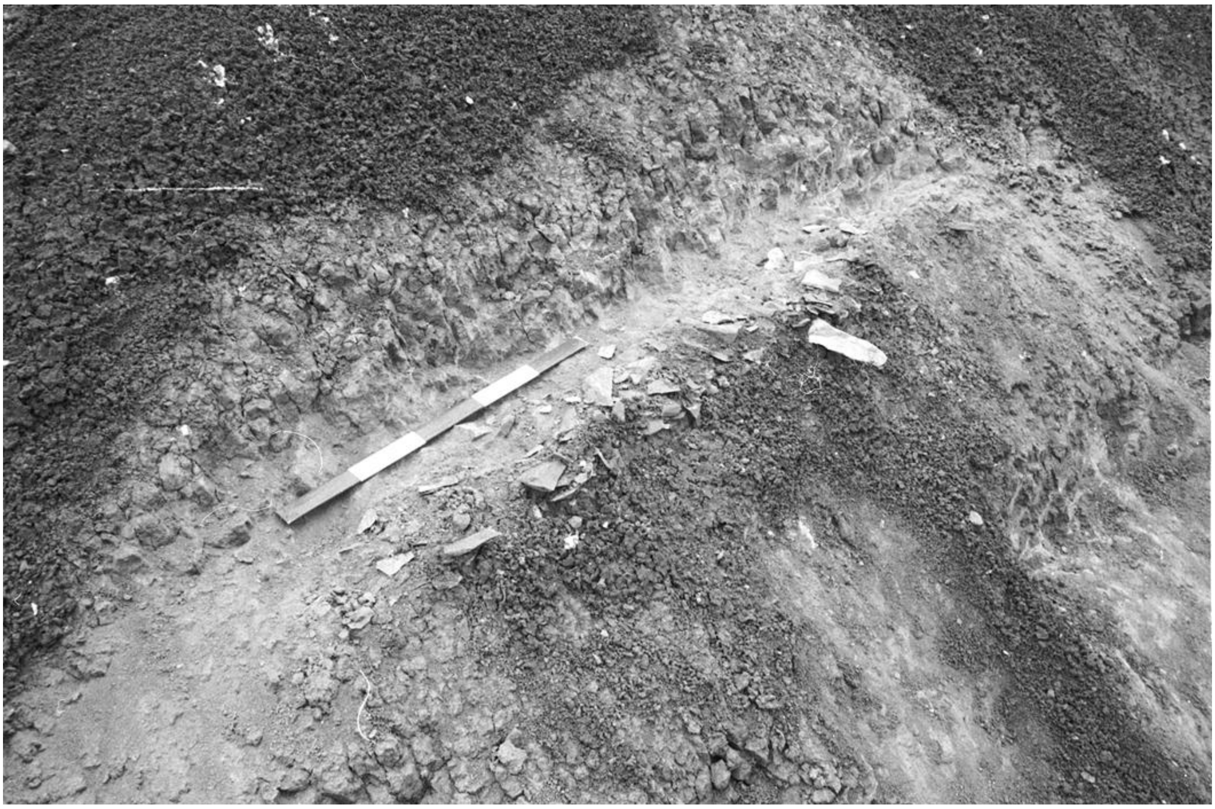

Supplementary Figure 39. Jojosi 1 during excavation in 1991. The tight clustering and multiple layers of hornfels artefacts on top of each other are visible and reminiscent of the situation at Jojosi 5, 6, and 7.

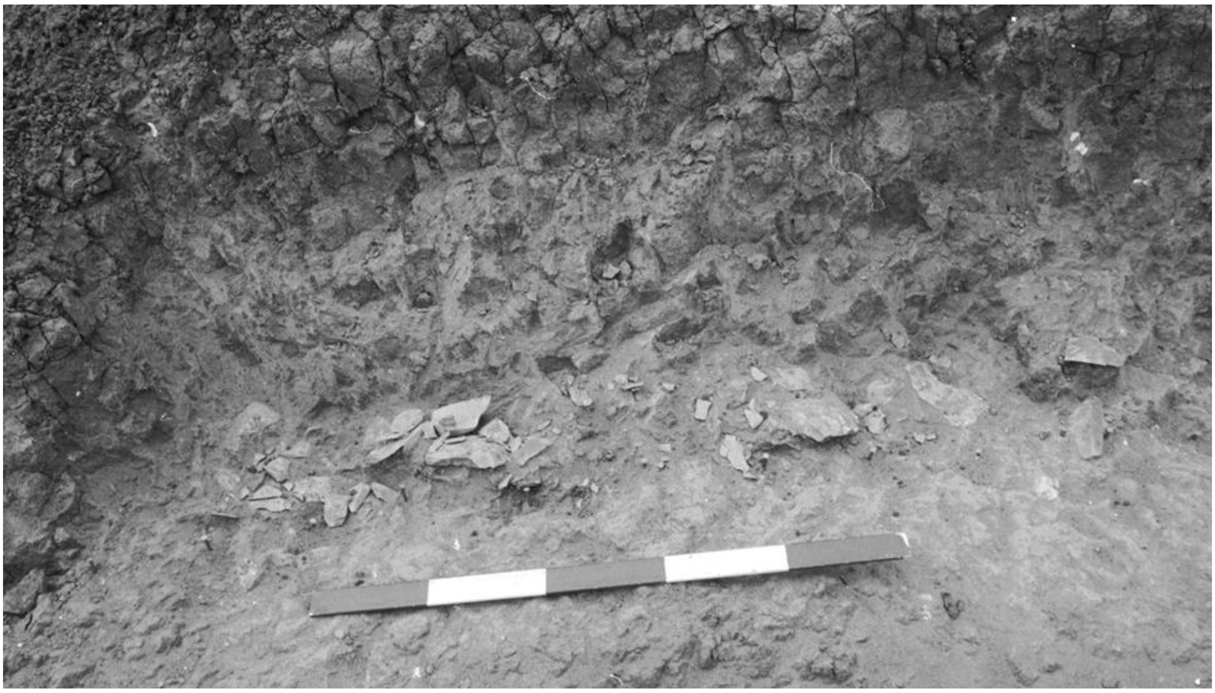

Supplementary Figure 40. Close-up on Jojosi 1 during excavations. Note the fresh state of the hornfels artefacts, their tight spatial clustering, and the presence of small debitage.

Supplementary Note 4: Lithic assemblages of Jojosi 5, 6, and 7

All basic and advanced data on Jojosi 1 can be found in Möller et al.[3] unless otherwise stated in the following. This presentation focuses on the newly excavated and studied lithic assemblages from Jojosi 5, 6, and 7.

1. Rose diagrams

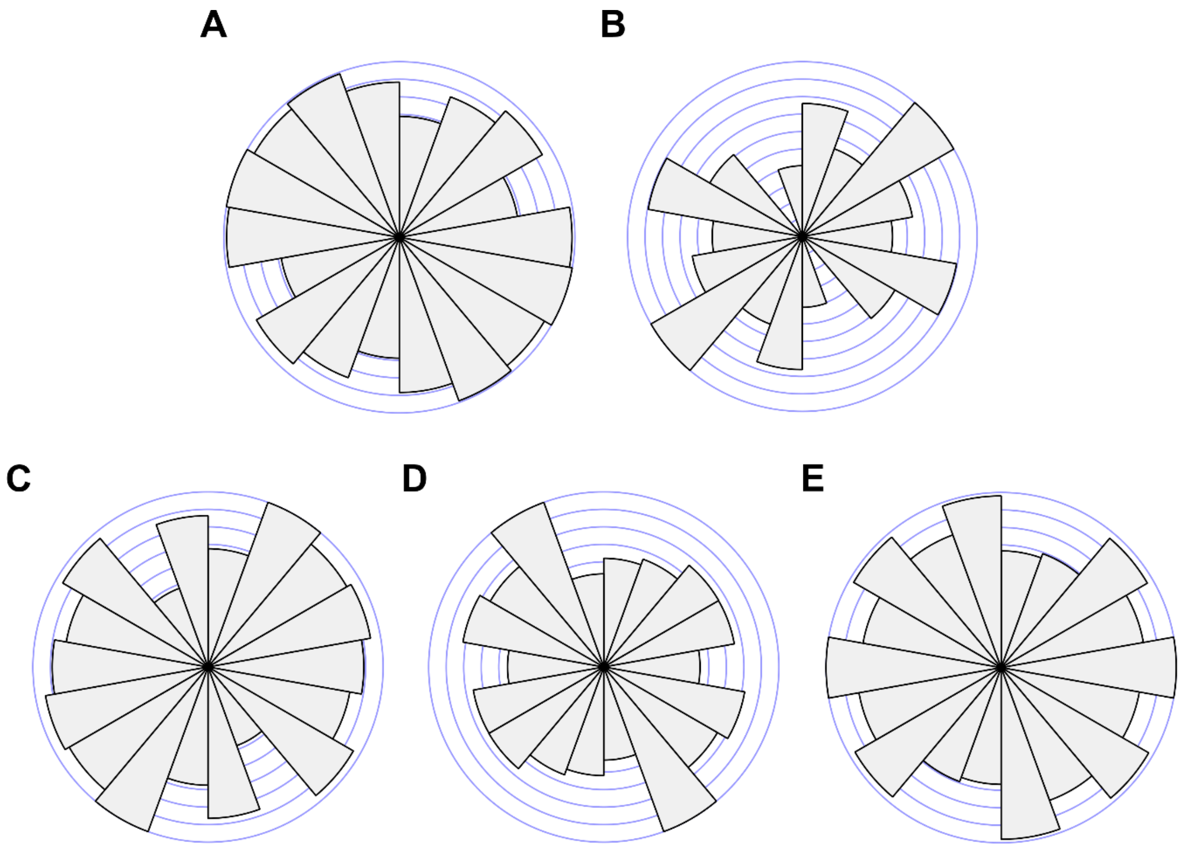

Supplementary Figure 41. Rose diagrams of artefacts from Jojosi 5, 6, and 7 whose orientation was recorded in the field with a Total Station. A: Jojosi 5 Lens 1, N=78 orientations. B: Jojosi 5 Lens 2, N=20 orientations. C: Jojosi 6 Lens 1, N=76 orientations. D: Jojosi 6 Lens 2, N=103 orientations. E: Jojosi 7 Lens 1, N=67 orientations. Data imported to QGIS from field measurements access database, creation of paths with points to path tool, and utilisation of the plugin "line direction histogram"(CITE) with 0-180° neutral orientation and 9 bins (equalling 20° bins) per mirrored half-circle projection. Following Sanderson & Peacock[27], a representation with proportional areas in the sense of an equal-area wedge diagram is chosen.

2. Size distributions and find densities

Supplementary Table 7. Overview of different size categories of artefacts recovered at all Jojosi sites. Note the very high amount and proportion of small artefacts <20 mm.

Site	1-5 mm	6-10 mm	11-20 mm	>20 mm	Total	% Lithics >20 mm
Jojosi 1*	3889		2408	1232	7529	16.4%
Jojosi 5	706	642	317	224	1889	11.9%
Jojosi 6	8763	4189	1648	720	15320	4.7%
Jojosi 7**	2080		1047	499	3626	13.8%
**Summed Total**	20269		5420	2675	28364	9.4%

*Jojosi 1 numbers derive from the 1991 Mazel excavations, with <10 mm as the smallest size category

**Due to the wet nature of the sediments, no systematic sieving and only hand-picking of small artefacts from the screens could be performed, with <10 mm as the smallest size category.

Supplementary Table 8. Overview of find densities at Jojosi 5, 6, and 7 tabulated by excavated context. Note the very high densities of lithic finds within the artefact lenses and the extreme discrepancy between densities of the artefact lenses and the overburden of several orders of magnitude.

Assemblage	Total lithics	m^3 excavated	Density (n/m^3)	Relative to overburden*
Jojosi 5, Lens 1	1107	0.004	276,750	x413
Jojosi 5, Lens 2	782	0.0035	223,428	x333
Jojosi 5 overburden**	351	0.524	670	-
Jojosi 6, Lens 1	8187	0.009	909,667	x548
Jojosi 6, Lens 2	7133	0.003	2,377,667	x1433
Jojosi 6 overburden**	506	0.305	1,659	-
**Summed Total**	18066	0.8485	n.a.	n.a.

* Multiples relative to overburden: Relative to overburden = Density (Jojosi Lens X) / Density (Overburden X)

** Overburden refers to all sediments adjacent to, above, or below the artefact lenses in a strict sense.

3. Refitting data

Supplementary Table 9. Overview of the results of the refitting studies at all 6 archaeological sites in the Jojosi dongas.

Site	Number of refit groups (n)	Number of total lithic artefacts (n)	Number of refitted and conjoined artefacts (n)	Share of refitted and conjoined artefacts (%)
Jojosi 1	20	820	48	5.9%
Jojosi 5 Lens 1	12	153	36	23.5%
Jojosi 5 Lens 2	6	71	35	49.3%
Jojosi 6 Lens 1	22	305	66	21.6%
Jojosi 6 Lens 2	33	414	90	21.7%
Jojosi 7 Lens 1	30	499	78	15.6%
Summed Total	123	2262	353	15.6%

Supplementary Table 10. Jojosi 5 Lens 1, overview of N=36 refitted and conjoined artefacts within their Refit Groups (RG). Note: RG 19 does not exist in this table due to the fact that, after data collection, the artefacts were refitted and integrated into another refit group.

RG	Inv. No. of involved artefacts	Type of refitting	No. of artefacts in RG (n)
7	716, 717, 818	Break	3
8	845, 846	Break	2
9	748, 787, 840	Break, Production sequence	3
10	704, 722, 798	Break	3
11	728, 769	Break	2
12	839,850	Break	3
13	757, 823	Break	2
14	702, 796	Production sequence	2
15	764, 788	Break	2
16	712, 715, 813	Break, Production sequence	3
17	710, 725, 753, 786, 827, 836, 838, 841, 849	Break, Production sequence	9
18	763, 835	Break	2
Summed total (n)			36
Share of total artefacts			23.5%
Arithmetic mean (n)			3.0
Standard Deviation (n)			1.9

Supplementary Table 11. Jojosi 5 Lens 2, overview of N=34 refitted and conjoined artefacts within their Refit Groups (RG).

RG	Inv. No. of involved artefacts	Type of refitting	No. of artefacts in RG (n)
1	859, 863	Break	2
2	867, 870, 887	Break, Production sequence	3
3	901, 875	Production sequence	2
4	860, 892, 893, 905	Production sequence	4
5	857, 874, 889, 894, 921	Break, Production sequence	5
6	858, 864, 865, 866, 868, 869, 878, 884, 890, 891, 898, 902, 904, 906, 910, 913, 914, 915, 918	Break, Production sequence	19
Summed total (n)			35
Share of total artefacts			49.3%
Arithmetic mean (n)			5.8
Standard Deviation (n)			6.0

Supplementary Table 12. Jojosi 6 Lens 1, overview of N=66 refitted and conjoined artefacts within their Refit Groups (RG). Note: RG 42 does not exist in this table due to the fact that, after data collection, the artefacts were refitted and integrated into another refit group.

RG	Inv. No. of involved artefacts	Type of refitting	No. of artefacts in RG (n)
20	194, 282	Break	2
21	169, 323	Break	2
22	184,289	Production sequence	2
23	100, 213,346	Break and Production sequence	3
24	133, 217, 283	Break and Production sequence	3
25	190, 237, 352	Break and Production sequence	3
26	122, 137, 332	Break and Production sequence	3
27	115, 118, 167, 226, 291	Production sequence	5
28	260, 264	Break	2
29	204, 320	Production sequence	2
30	243, 327	Break	2
31	101, 349	Production sequence	2
32	127, 223, 291	Production sequence	3
33	102, 306	Break	2
34	108, 263	Production sequence	2
35	141, 170	Break	2
36	139, 138	Break	2
37	178, 272	Production sequence	2
38	9.2, 9.3, 109, 111, 113, 114, 160, 165, 196, 209, 210, 238, 269, 305, 319	Break and Production sequence	15
39	9.15, 343, 347	Production sequence	3
40	9.12, 247	Production sequence	2
41	198, 9.28	Production sequence	2
Summed total (n)			66
Share of total artefacts			21.6%
Arithmetic mean (n)			3.0
Standard Deviation (n)			2.7

Supplementary Table 13. Jojosi 6 Lens 2, overview of N=66 refitted and conjoined artefacts within their Refit Groups (RG).

RG	Inv. No. of involved artefacts	Type of refitting	No. of artefacts in RG (n)
73	393, 603	Break	2
74	604, 741.40	Break	2
75	375, 648	Production sequence	2
76	368, 656	Production sequence	2
77	377, 654	Production sequence	2
78	377, 654	Break	2
79	431, 644, 683, 714	Break and Production sequence	4
80	380, 547, 612	Break and Production sequence	3
81	457, 493	Production sequence	2
82	449, 651	Break	2
83	453, 649	Break	2
84	430, 433	Break	2
85	459, 725	Break	2
86	539, 641	Break	2
87	533, 719	Break	2
88	598, 611	Break	2
89	425, 438, 475, 485, 614	Break and Production sequence	5
90	486, 590	Break	2
91	741.11, 741.23	Break	2
92	741.37, 581	Break	2
93	473, 489	Break	2
94	569, 599	Break	2
95	741.25, 741.42	Break	2
96	423, 517	Break	2
97	472, 506	Break	2
98	411, 707	Break	2
99	542, 628	Break	2
100	477, 478, 491, 518, 532, 545, 583, 601, 693	Break and Production sequence	9
101	527, 552, 661, 678, 679, 704	Break and Production sequence	6
102	386, 390, 391	Break and Production sequence	3
103	384, 427, 567, 573, 703	Break and Production sequence	5
104	448, 504	Break	2
105	480, 501, 502, 546, 586	Break and Production sequence	5
Summed total (n)			90
Share of total artefacts			21.7%
Arithmetic mean (n)			2.7
Standard Deviation (n)			1.6

Supplementary Table 14. Jojosi 7 Lens 1, overview of N=78 refitted and conjoined artefacts within their Refit Groups (RG).

RG	Inv. No. of involved artefacts	Type of refitting	No. of artefacts in RG (n)
43	415, 416	Break	2
44	223, 272	Production sequence	2
45	8, 228, 271	Production sequence	3
46	125, 241, 252	Break	3
47	94, 217, 401	Production sequence	3
48	280, 409	Production sequence	2
49	17.49, 269	Break	2
50	76, 142, 145, 209, 290, 363	Break and Production sequence	6
51	54, 277	Break	2
52	169, 226	Production sequence	2
53	72, 129, 218, 229, 298, 299, 303	Production sequence	7
54	112, 131, 159, 324	Break and Production sequence	4
55	92, 121, 126	Break	3
56	133, 187, 366	Break	3
57	17.71, 346. 348	Break and Production sequence	3
58	68, 198	Break	2
59	369, 370	Break	2
60	28, 31	Break	2
61	157, 207	Break	2
62	124, 141	Production sequence	2
63	17.6, 203	Break	2
64	17.37, 17.41	Production sequence	2
65	17.34, 257	Break	2
66	11, 17.47	Break	2
67	312, 420	Production sequence	2
68	40, 41	Break	2
69	405, 407, 410	Production sequence	3
70	310, 375	Break	2
71	143, 166	Break	2
72	347, 437	Break	2
Summed total (n)			78
Share of total artefacts			15.6%
Arithmetic mean (n)			2.6
Standard Deviation (n)			1.2

4. Lithic analysis: Basic results

Supplementary Table 15. Jojosi 5 Lens 1, general find types of the n=153 recorded lithic artefacts.

Site and feature	Blank (n)	Core (n)	Tool (n)	Angular Debris (n)	Summed total
Jojosi 5 Lens 1	149	0	0	4	153
Share of total	97.4%	0.0%	0.0%	2.6%	100.0%

Supplementary Table 16. Jojosi 5 Lens 1, blank types of n=149 lithic blanks.

Site and feature	Flake (n)	Blade (n)	Bladelet (n)	Point (n)	Summed total
Jojosi 5 Lens 1	119	27	2	1	149
Share of total	79.9%	18.1%	1.3%	0.7%	100.0%

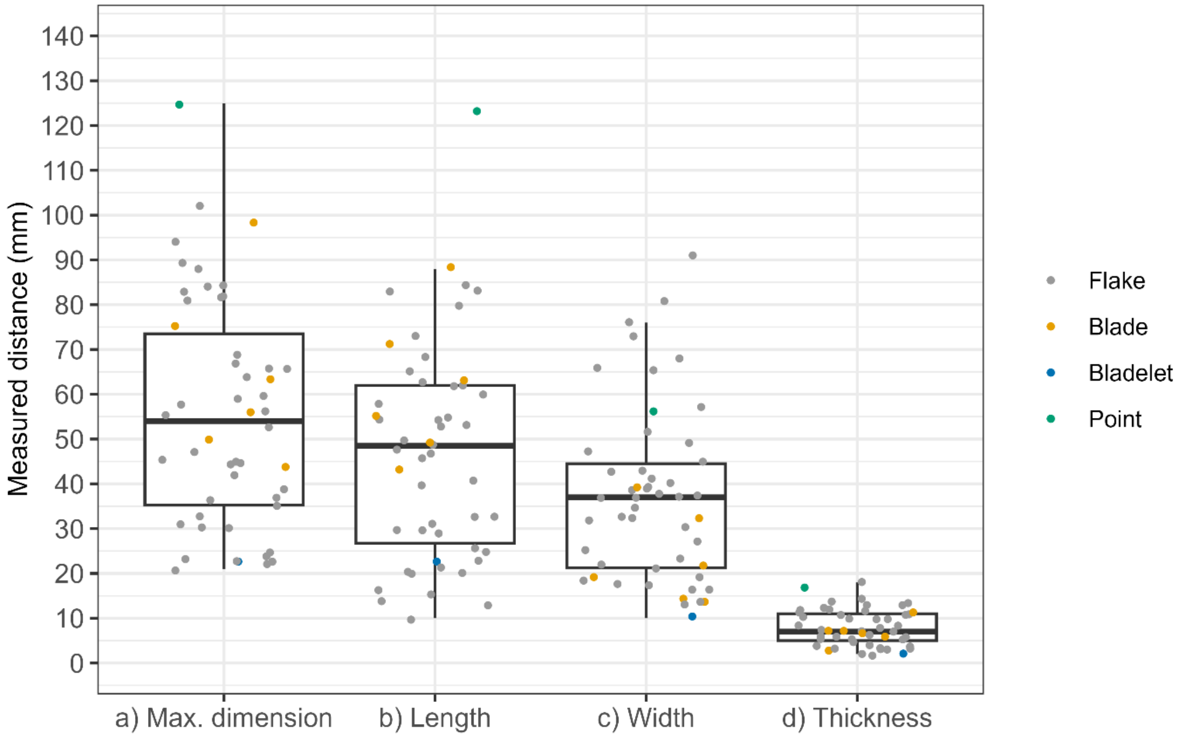

Supplementary Figure 42. Jojosi 5 Lens 1, metric dimensions of n=50 complete blanks.

Supplementary Table 17. Jojosi 5 Lens 1, descriptive statistics of the metric dimensions of n=50 complete blanks.

Measured distance		Flake	Blade	Bladelet	Point	All blanks
Maximum Dimension	x̄ (mm)	53.4	64.3	23.0	125.0	55.5
	σ (mm)	23.5	19.7	NA	NA	25.2
	Minimum (mm)	21.0	44.0	23.0	125.0	21.0
	Maximum (mm)	102.0	98.0	23.0	125.0	125.0
	Skewness	0.3	0.8	NA	NA	0.5
	Kurtosis	1.9	2.4	NA	NA	2.6
Length	x̄ (mm)	43.8	61.5	23.0	123.0	47.1
	σ (mm)	21.5	16.3	NA	NA	24.0
	Minimum (mm)	10.0	43.0	23.0	123.0	10.0
	Maximum (mm)	84.0	88.0	23.0	123.0	123.0
	Skewness	0.2	0.5	NA	NA	0.6
	Kurtosis	2.0	2.2	NA	NA	3.4
Width	x̄ (mm)	39.3	23.3	10.0	56.0	37.1
	σ (mm)	19.5	10.2	NA	NA	19.4
	Minimum (mm)	13.0	14.0	10.0	56.0	10.0
	Maximum (mm)	91.0	39.0	10.0	56.0	91.0
	Skewness	0.8	0.6	NA	NA	0.8
	Kurtosis	3.1	1.8	NA	NA	3.2
Thickness	x̄ (mm)	8.0	6.8	2.0	17.0	7.9
	σ (mm)	4.0	2.6	NA	NA	4.1
	Minimum (mm)	2.0	3.0	2.0	17.0	2.0
	Maximum (mm)	18.0	11.0	2.0	17.0	18.0
	Skewness	0.3	0.2	NA	NA	0.4
	Kurtosis	2.2	2.9	NA	NA	2.3
	N	42	6	1	1	50

Supplementary Table 18. Jojosi 5 Lens 2, general find types of the n=71 recorded lithic artefacts.

Site and feature	Blank (n)	Core (n)	Tool (n)	Summed total
Jojosi 5 Lens 2	65	4	2 (hammerstone)	71
Share of total	91.5%	5.6%	2.8%	100.0%

Supplementary Table 19. Jojosi 5 Lens 2, blank types of n=65 lithic blanks.

Site and feature	Flake (n)	Blade (n)	Bladelet (n)	Summed total
Jojosi 5 Lens 2	48	16	1	65
Share of total	73.8%	24.6%	1.5%	100.0%

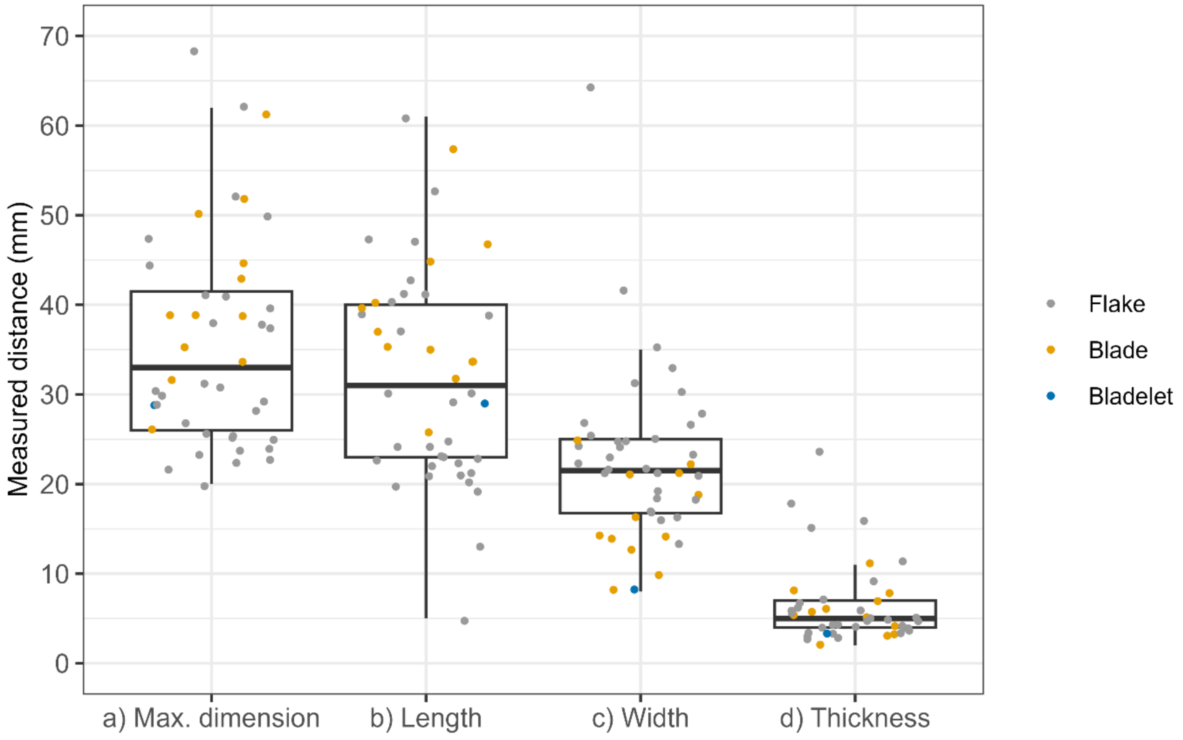

Supplementary Figure 43. Jojosi 5 Lens 2, metric dimensions of n=44 complete blanks.

Supplementary Table 20. Jojosi 5 Lens 2, descriptive statistics of the metric dimensions of n=44 complete blanks.

Measured distance		Flake	Blade	Bladelet	All blanks
Maximum Dimension	$\bar{x}$ (mm)	33.9	41.3	29.0	35.8
	σ (mm)	12.1	9.6	NA	11.7
	Minimum (mm)	20.0	26.0	29.0	20.0
	Maximum (mm)	68.0	61.0	29.0	68.0
	Skewness	1.2	0.5	NaN	0.9
	Kurtosis	3.8	2.7	NaN	3.1
Length	$\bar{x}$ (mm)	29.9	38.5	29.0	32.2
	σ (mm)	12.5	8.1	NA	11.9
	Minimum (mm)	5.0	26.0	29.0	5.0
	Maximum (mm)	61.0	57.0	29.0	61.0
	Skewness	0.6	0.8	NaN	0.3
	Kurtosis	2.8	3.4	NaN	2.8
Width	$\bar{x}$ (mm)	25.0	16.4	8.0	22.3
	σ (mm)	9.5	5.2	NA	9.5
	Minimum (mm)	13.0	8.0	8.0	8.0
	Maximum (mm)	64.0	25.0	8.0	64.0
	Skewness	2.4	0.1	NaN	2.0
	Kurtosis	10.5	2.0	NaN	10.1
Thickness	$\bar{x}$ (mm)	6.6	5.7	3.0	6.3
	σ (mm)	5.0	2.6	NA	4.5
	Minimum (mm)	3.0	2.0	3.0	2.0
	Maximum (mm)	24.0	11.0	3.0	24.0
	Skewness	2.0	0.5	NaN	2.2
	Kurtosis	6.5	2.6	NaN	8.1
	N	31	12	1	44

Supplementary Table 21. Jojosi 6 Lens 1, general find types of the n=306 recorded lithic artefacts.

Site and feature	Blank (n)	Core (n)	Tool (n)	Angular Debris (n)	Summed total
Jojosi 6 Lens 1	295	1	0	9	306
Share of total	96.7%	0.3%	0.0%	3.0%	100.0%

Supplementary Table 22. Jojosi 6 Lens 1, blank types of n=295 lithic blanks.

Site and feature	Flake (n)	Blade (n)	Bladelet (n)	Point (n)	Summed total
Jojosi 6 Lens 1	257	32	6	0	295
Share of total	87.1%	10.8%	2.0%	0.0%	100.0%

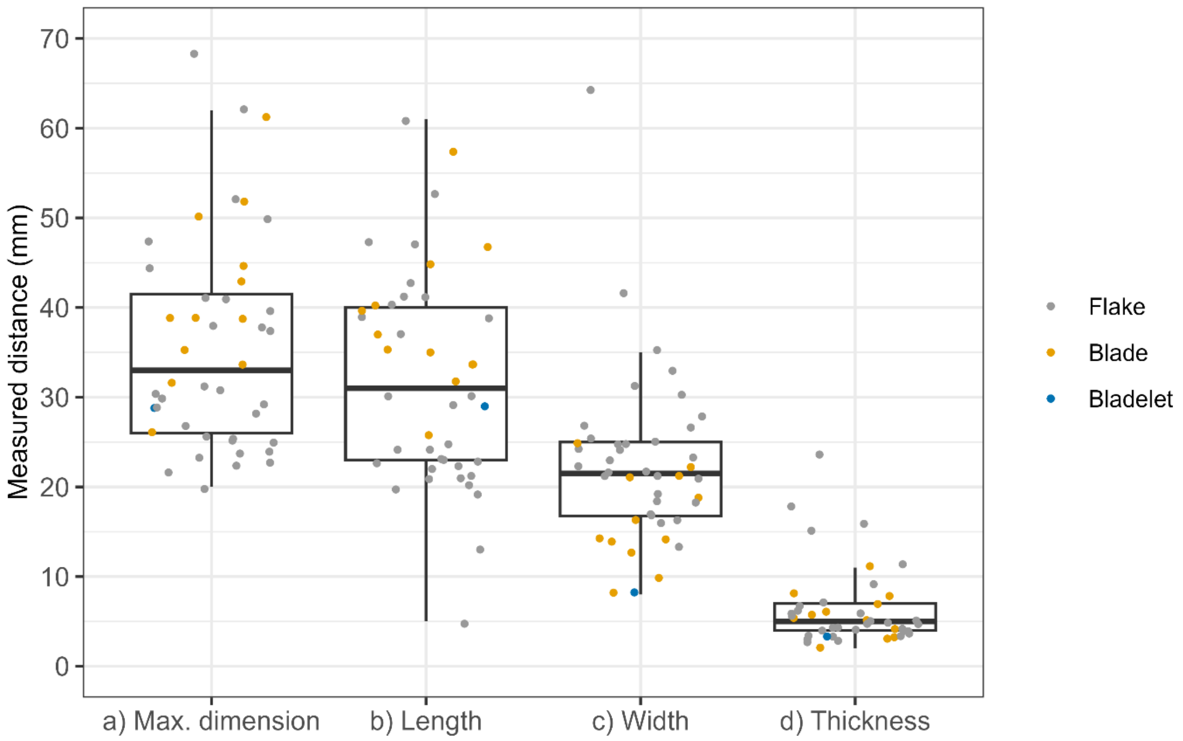

Supplementary Figure 44. Jojosi 6 Lens 1, metric dimensions of n=75 complete blanks.

Supplementary Table 23. Jojosi 6 Lens 1, descriptive statistics of the metric dimensions of n=75 complete blanks.

Measured distance		Flake	Blade	Bladelet	All blanks
Maximum Dimension	x̄ (mm)	43.6	62.7	22.7	44.5
	σ (mm)	19.9	23.3	2.3	20.9
	Minimum (mm)	20.0	33.0	20.0	20.0
	Maximum (mm)	95.0	97.0	24.0	97.0
	Skewness	1.1	0.2	-0.7	1.0
	Kurtosis	3.2	1.9	1.5	3.0
Length	x̄ (mm)	37.1	59.3	22.3	38.6
	σ (mm)	17.2	23.7	2.1	18.8
	Minimum (mm)	15.0	30.0	20.0	15.0
	Maximum (mm)	88.0	94.0	24.0	94.0
	Skewness	1.2	0.4	-0.5	1.2
	Kurtosis	3.8	1.8	1.5	3.7
Width	x̄ (mm)	31.6	20.1	9.0	29.6
	σ (mm)	16.6	9.5	2.0	16.6
	Minimum (mm)	2.0	11.0	7.0	2.0
	Maximum (mm)	97.0	35.0	11.0	97.0
	Skewness	1.2	0.7	0.0	1.3
	Kurtosis	5.4	1.9	1.5	5.4
Thickness	x̄ (mm)	6.9	5.9	2.7	6.6
	σ (mm)	5.3	3.0	1.2	5.1
	Minimum (mm)	1.0	2.0	2.0	1.0
	Maximum (mm)	23.0	9.0	4.0	23.0
	Skewness	1.3	-0.3	0.7	1.4
	Kurtosis	3.8	1.6	1.5	4.2
	N	65	7	3	75

Supplementary Table 24. Jojosi 6 Lens 2, general find types of the n=414 recorded lithic artefacts.

Site and feature	Blank (n)	Core (n)	Tool (n)	Angular Debris (n)	Summed total
Jojosi 6 Lens 2	405	0	0	9	414
Share of total	97.8%	0.0%	0.0%	2.2%	100.0%

Supplementary Table 25. Jojosi 6 Lens 2, blank types of n=405 lithic blanks.

Site and feature	Flake (n)	Blade (n)	Bladelet (n)	Point (n)	Summed total
Jojosi 6 Lens 2	347	52	6	0	405
Share of total	85.7%	12.8%	1.5%	0.0%	100.0%

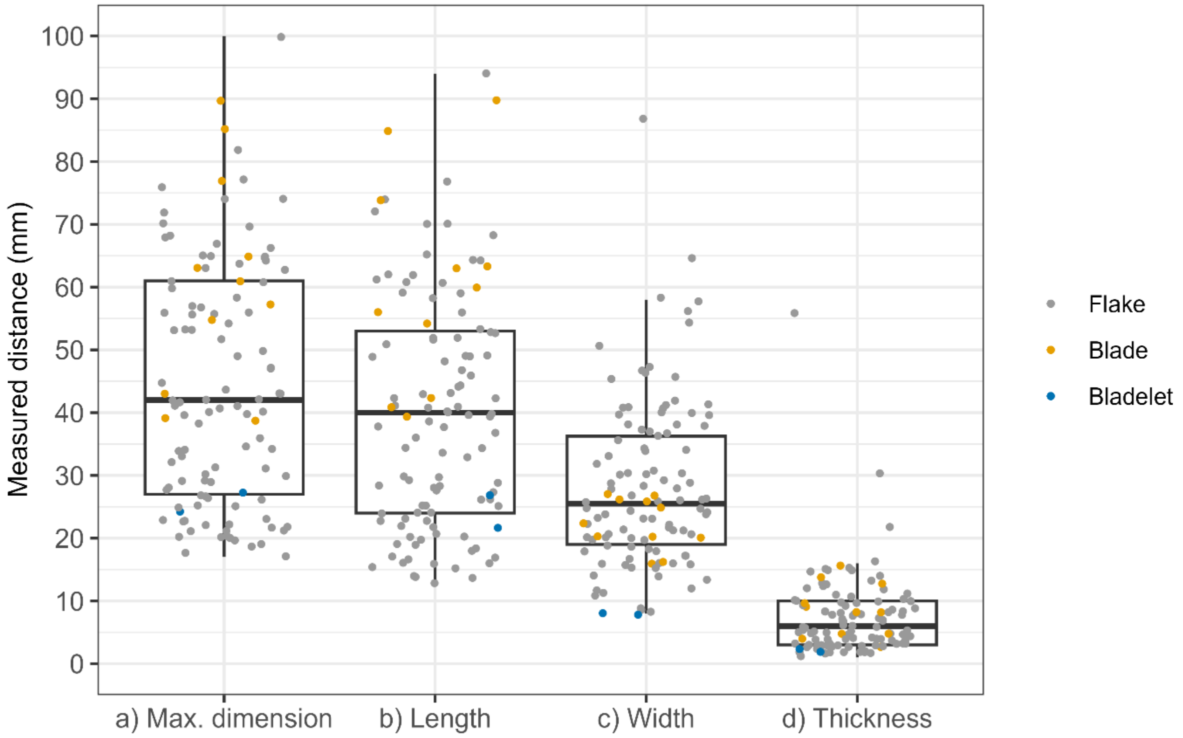

Supplementary Figure 45. Jojosi 6 Lens 2, metric dimensions of n=112 complete blanks.

Supplementary Table 26. Jojosi 6 Lens 2, descriptive statistics of the metric dimensions of n=112 complete blanks.

Measured distance		Flake	Blade	Bladelet	All blanks
Maximum Dimension	x̅ (mm)	43.2	61.3	25.5	44.7
	σ (mm)	18.7	17.4	2.1	19.3
	Minimum (mm)	17.0	39.0	24.0	17.0
	Maximum (mm)	100.0	90.0	27.0	100.0
	Skewness	0.5	0.2	0.0	0.5
	Kurtosis	2.4	2.0	1.0	2.3
Length	x̅ (mm)	38.5	60.6	24.5	40.4
	σ (mm)	17.9	17.1	3.5	19.0
	Minimum (mm)	13.0	39.0	22.0	13.0
	Maximum (mm)	94.0	90.0	27.0	94.0
	Skewness	0.6	0.4	0.0	0.5
	Kurtosis	2.6	2.1	1.0	2.6
Width	x̅ (mm)	28.9	22.3	8.0	27.9
	σ (mm)	13.6	4.2	0.0	13.3
	Minimum (mm)	8.0	16.0	8.0	8.0
	Maximum (mm)	87.0	27.0	8.0	87.0
	Skewness	1.2	-0.3	NaN	1.3
	Kurtosis	5.3	1.7	NaN	5.7
Thickness	x̅ (mm)	28.9	22.3	8.0	7.5
	σ (mm)	13.6	4.2	0.0	6.6
	Minimum (mm)	8.0	16.0	8.0	1.0
	Maximum (mm)	87.0	27.0	8.0	56.0
	Skewness	1.2	-0.3	NaN	4.0
	Kurtosis	5.3	1.7	NaN	28.1
	N	99	11	2	112

Supplementary Table 27. Jojosi 7 Lens 1, general find types of the n=499 recorded lithic artefacts.

Site and feature	Blank (n)	Core (n)	Tool (n)	Angular Debris (n)	Summed total
Jojosi 7 Lens 1	481	3	6	9	499
Share of total	96.4%	0.6%	1.2%	1.8%	100.0%

Supplementary Table 28. Jojosi 7 Lens 1, blank types of n=499 lithic blanks.

Site and feature	Flake (n)	Blade (n)	Bladelet (n)	Point (n)	Summed total
Jojosi 7 Lens 1	412	57	12	0	481
Share of total	85.7%	11.9%	2.5%	0.0%	100.0%

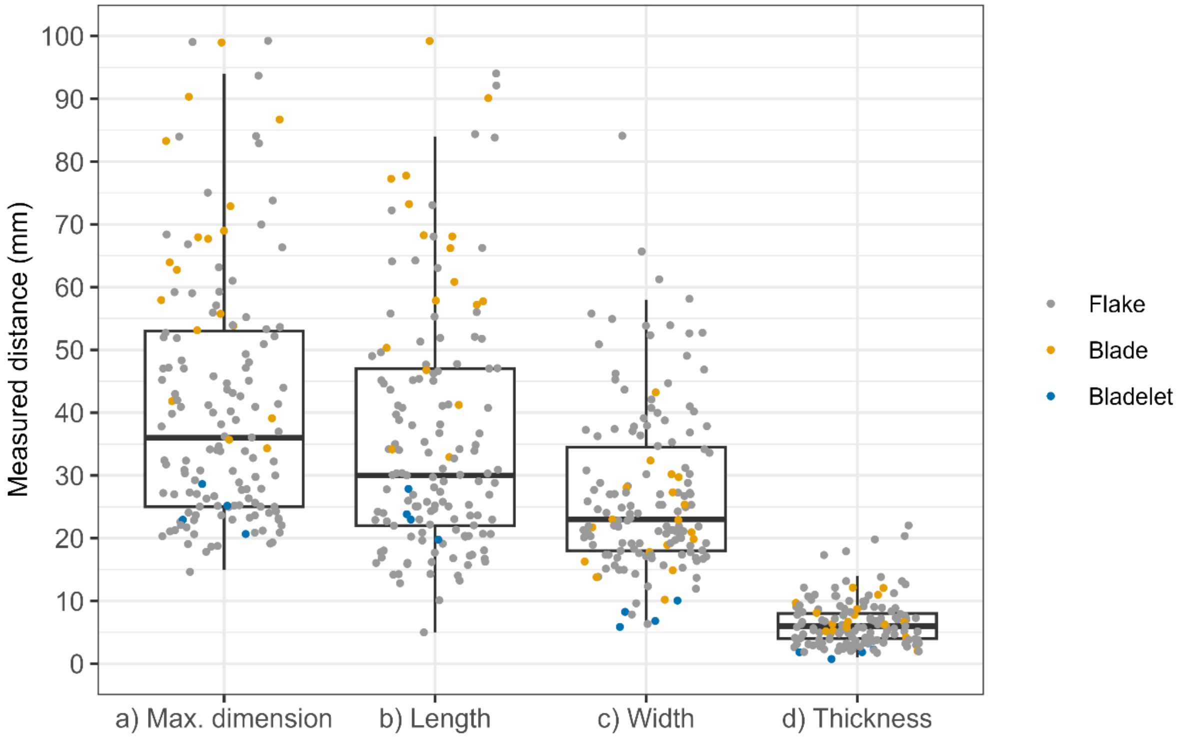

Supplementary Figure 46. Jojosi 7 Lens 1, metric dimensions of n=147 complete blanks.

Supplementary Table 29. Jojosi 7 Lens 1, descriptive statistics of the metric dimensions of n=147 complete blanks.

Measured distance		Flake	Blade	Bladelet	All blanks
Maximum Dimension	$\bar{x}$ (mm)	38.9	63.1	24.5	41.5
	σ (mm)	18.1	18.8	3.4	19.8
	Minimum (mm)	15.0	34.0	21.0	15.0
	Maximum (mm)	99.0	99.0	29.0	99.0
	Skewness	1.3	0.2	0.4	1.1
	Kurtosis	4.4	2.2	1.8	3.5
Length	$\bar{x}$ (mm)	33.6	62.2	23.8	36.7
	σ (mm)	17.5	18.3	3.3	19.7
	Minimum (mm)	5.0	33.0	20.0	5.0
	Maximum (mm)	94.0	99.0	28.0	99.0
	Skewness	1.3	0.2	0.3	1.1
	Kurtosis	4.6	2.5	2.0	3.6
Width	$\bar{x}$ (mm)	28.3	23.1	7.8	27.1
	σ (mm)	13.5	7.8	1.7	13.2
	Minimum (mm)	6.0	10.0	6.0	6.0
	Maximum (mm)	84.0	43.0	10.0	84.0
	Skewness	1.2	0.7	0.4	1.3
	Kurtosis	4.7	3.5	1.8	4.9
Thickness	$\bar{x}$ (mm)	28.3	23.1	7.8	27.1
	σ (mm)	13.5	7.8	1.7	13.2
	Minimum (mm)	6.0	10.0	6.0	6.0
	Maximum (mm)	84.0	43.0	10.0	84.0
	Skewness	1.2	0.7	0.4	1.2
	Kurtosis	4.7	3.5	1.8	4.9
	N	125	18	4	147

5. Lithic analysis: Techno-typological results

Supplementary Table 30. Jojosi 5 Lens 1, technological typology of n=119 flake blanks.

Reduction stage	Flake type	n	Share
Initial stage	Cortical flake	11	9.2%
Main production and core maintenance	debordant	29	24.4%
	debordant a dos limité	3	2.5%
	Kombewa flake	1	0.8%
	plunging flake	8	6.7%
	preferential flake	1	0.8%
Other	non-diagnostic flake	66	55.5%
Summed total		119	100.0%

Supplementary Table 31. Jojosi 5 Lens 1, technological typology of n=27 blade blanks.

Reduction stage	Blade type	n	Share
Initial stage	A1	1	3.7%
	A3	3	11.1%
Main production and core maintenance	B1	6	22.2%
	B2	1	3.7%
	B4	2	7.4%
	B6	8	29.6%
	B7	2	7.4%
	D2	2	7.4%
Other	E1	2	7.4%
Summed total		27	100.0%

Supplementary Table 32. Jojosi 5 Lens 1, the amount of cortex on the dorsal face of all blanks. Altogether, n=92 (61.7%) of all blanks exhibit some cortical surface on their dorsal face.

Dorsal cortex amount (%)	N of Flakes (%)	N of Blades (%)	N of Bladelets (%)	N of Points (%)	N of Summed Total (%)
0	45 (37.8%)	10 (37.0%)	2 (100%)	0 (0.0%)	57 (38.3%)
10	20 (16.8%)	4 (14.8%)	0 (0.0%)	0 (0.0%)	24 (16.1%)
20	11 (9.2%)	2 (7.4%)	0 (0.0%)	1 (100.0%)	14 (9.4%)
30	11 (9.2%)	4 (14.8%)	0 (0.0%)	0 (0.0%)	15 (10.1%)
40	3 (2.5%)	3 (11.1%)	0 (0.0%)	0 (0.0%)	6 (4.0%)
50	4 (3.4%)	2 (7.4%)	0 (0.0%)	0 (0.0%)	6 (4.0%)
60	3 (2.5%)	0 (0.0%)	0 (0.0%)	0 (0.0%)	3 (2%)
70	1 (0.8%)	0 (0.0%)	0 (0.0%)	0 (0.0%)	1 (0.7%)
80	7 (5.9%)	2 (7.4%)	0 (0.0%)	0 (0.0%)	9 (6.0%)
90	8 (6.7%)	0 (0.0%)	0 (0.0%)	0 (0.0%)	8 (5.4%)
100	6 (5.0%)	0 (0.0%)	0 (0.0%)	0 (0.0%)	6 (4%)
N of Summed Total (%)	119 (79.9%)	27 (18.1%)	2 (1.3%)	1 (0.7%)	149 (100.0%)

Supplementary Table 33. Jojosi 5 Lens 2, technological typology of n=48 flake flanks.

Reduction stage	Flake type	n	Share
Initial stage	Cortical flake	4	8.3%
Main production and core maintenance	débordant	18	37.5%
	débordant a dos limité	5	10.4%
	preferential flake	1	2.1%
Other	non-diagnostic flake	20	41.7%
Summed total		48	100.0%

Supplementary Table 34. Jojosi 5 Lens 2, technological typology of n=16 blade blanks.

Reduction stage	Blade type	n	Share
Initial stage	A3	1	6.3%
Main production and core maintenance	B1	3	18.8%
	B3	1	6.3%
	B4	2	12.5%
	B6	4	25.0%
	B7	2	12.5%
	B8	2	12.5%
Other	E1	1	6.3%
Summed total		16	100.0%

Supplementary Table 35. Jojosi 5 Lens 2, amount of cortex on the dorsal face of all blanks. Altogether, n=35 (53.8%) of all blanks exhibit some cortical surface on their dorsal face.

Dorsal cortex amount (%)	N of Flakes (%)	N of Blades (%)	N of Bladelets (%)	N of Summed Total (%)
0	20 (41.7%)	9 (56.3%)	1 (100%)	30 (46.2%)
10	14 (29.2%)	3 (18.8%)	0 (0.0%)	17 (26.2%)
20	3 (6.3%)	2 (12.5%)	0 (0.0%)	5 (7.7%)
30	4 (8.3%)	1 (6.3%)	0 (0.0%)	5 (7.7%)
40	0 (0.0%)	0 (0.0%)	0 (0.0%)	0 (0.0%)
50	0 (0.0%)	0 (0.0%)	0 (0.0%)	0 (0.0%)
60	0 (0.0%)	0 (0.0%)	0 (0.0%)	0 (0.0%)
70	2 (4.2%)	0 (0.0%)	0 (0.0%)	2 (3.1%)
80	1 (2.1%)	1 (6.3%)	0 (0.0%)	2 (3.1%)
90	1 (2.1%)	0 (0.0%)	0 (0.0%)	1 (1.5%)
100	3 (6.3%)	0 (0.0%)	0 (0.0%)	3 (4.6%)
N of Summed Total (%)	48 (73.8%)	16 (24.6%)	1 (1.5%)	65 (100.0%)

Supplementary Table 36. Jojosi 6 Lens 1, technological typology of n=257 flake blanks.

Reduction stage	Flake type	n	Share
Initial stage	Cortical flake	24	9.3%
Main production and core maintenance	debordant	58	22.6%
	debordant a dos limité	10	3.9%
	plunging flake	11	4.3%
	preferential flake	1	0.4%
Other	non-diagnostic flake	153	59.5%
Summed total		257	100.0%

Supplementary Table 37. Jojosi 6 Lens 1, technological typology of n=32 blade blanks.

Reduction stage	Blade type	n	Share
Initial stage	A2	1	3.1%
	A3	4	12.5%
Main production and core maintenance	B1	16	50.0%
	B3	1	3.1%
	B6	4	12.5%
	B7	5	15.6%
Other	E1	1	3.1%
Summed total		32	100.0%

Supplementary Table 38. Jojosi 6 Lens 1, amount of cortex on the dorsal face of all blanks. Altogether, n=143 (48.5%) of all blanks exhibit some cortical surface on their dorsal face.

Dorsal cortex amount (%)	N of Flakes (%)	N of Blades (%)	N of Bladelets (%)	N of Summed Total (%)
0	127 (49.4%)	20 (62.5%)	5 (83.3%)	152 (51.5%)
10	40 (15.6%)	4 (12.5%)	1 (16.7%)	45 (15.3%)
20	19 (7.4%)	1 (3.1%)	0 (0%)	20 (6.8%)
30	7 (2.7%)	0 (0%)	0 (0%)	7 (2.4%)
40	12 (4.7%)	1 (3.1%)	0 (0%)	13 (4.4%)
50	4 (1.6%)	1 (3.1%)	0 (0%)	5 (1.7%)
60	8 (3.1%)	1 (3.1%)	0 (0%)	9 (3.1%)
70	2 (0.8%)	1 (3.1%)	0 (0%)	3 (1%)
80	12 (4.7%)	2 (6.3%)	0 (0%)	14 (4.7%)
90	5 (1.9%)	0 (0%)	0 (0%)	5 (1.7%)
100	21 (8.2%)	1 (3.1%)	0 (0%)	22 (7.5%)
N of Summed Total (%)	257 (87.1%)	32 (10.8%)	6 (2%)	295 (100%)

Supplementary Table 39. Jojosi 6 Lens 2, technological typology of n=347 flake blanks.

Reduction stage	Flake type	n	Share
Initial stage	Cortical flake	17	4.9%
Main production and core maintenance	Debordant	81	23.3%
	Debordant a dos limité	6	1.7%
	Kombewa flake	1	0.3%
	Plunging flake	9	2.6%
	Partial core tablet	1	0.3%
Other	non-diagnostic flake	232	66.9%
Summed total		347	100.0%

Supplementary Table 40. Jojosi 6 Lens 2, technological typology of n=52 blade blanks.

Reduction stage	Blade type	n	Share
Initial stage	A1	3	5.8%
	A2	2	3.9%
	A3	3	5.8%
Main production and core maintenance	B1	24	46.2%
	B6	10	19.2%
	B7	5	9.6%
Other	E1	5	9.6%
Summed total		52	100.0%

Supplementary Table 41. Jojosi 6 Lens 2, amount of cortex on the dorsal face of all blanks. Altogether, n=134 (33.1%) of all blanks exhibit some cortical surface on their dorsal face.

Dorsal cortex amount (%)	N of Flakes (%)	N of Blades (%)	N of Bladelets (%)	N of Summed Total (%)
0	233 (67.1%)	32 (61.5%)	6 (100%)	271 (66.9%)
10	24 (6.9%)	4 (7.7%)	0 (0.0%)	28 (6.9%)
20	20 (5.8%)	4 (7.7%)	0 (0.0%)	24 (5.9%)
30	17 (4.9%)	4 (7.7%)	0 (0.0%)	21 (5.2%)
40	8 (2.3%)	0 (0.0%)	0 (0.0%)	8 (2.0%)
50	6 (1.7%)	1 (1.9%)	0 (0.0%)	7 (1.7%)
60	4 (1.2%)	1 (1.9%)	0 (0.0%)	5 (1.2%)
70	9 (2.6%)	1 (1.9%)	0 (0.0%)	10 (2.5%)
80	6 (1.7%)	1 (1.9%)	0 (0.0%)	7 (1.7%)
90	12 (3.5%)	1 (1.9%)	0 (0.0%)	13 (3.2%)
100	8 (2.3%)	3 (5.8%)	0 (0.0%)	11 (2.7%)
N of Summed Total (%)	347 (85.7%)	52 (12.8%)	6 (1.5%)	405 (100%)

Supplementary Table 42. Jojosi 7 Lens 1, technological typology of n=411 flake blanks.

Reduction stage	Flake type	n	Share
Initial stage	Cortical flake	29	7.1%
Main production and core maintenance	Core preparation flake	1	0.2%
	debordant	104	25.3%
	debordant a dos limité	13	3.2%
	Kombewa flake	2	0.5%
	Plunging flake	8	1.9%
	Preferential flake	2	0.5%
Other	non-diagnostic flake	252	61.3%
Summed total		411	100.0%

Supplementary Table 43. Jojosi 7 Lens 1, technological typology of n=58 blade blanks.

Reduction stage	Blade type	n	Share
Initial stage	A1	1	1.7%
	A2	6	10.3%
	A3	3	5.2%
Main production and core maintenance	B1	29	50.0%
	B4	1	1.7%
	B6	13	22.4%
	B7	2	3.4%
	D2	2	3.4%
Other	E1	1	1.7%
Summed total		58	100.0%

Supplementary Table 44. Jojosi 6 Lens 2, amount of cortex on the dorsal face of all blanks. Altogether, 171 (35.6%) of all blanks exhibit some cortical surface on their dorsal face.

Dorsal cortex amount (%)	N of Flakes (%)	N of Blades (%)	N of Bladelets (%)	N of Summed Total (%)
0	270 (65.7%)	34 (58.6%)	6 (50%)	310 (64.4%)
10	47 (11.4%)	3 (5.2%)	0 (0.0%)	50 (10.4%)
20	22 (5.4%)	3 (5.2%)	3 (25%)	28 (5.8%)
30	16 (3.9%)	7 (12.1%)	1 (8.3%)	24 (5%)
40	2 (0.5%)	1 (1.7%)	0 (0.0%)	3 (0.6%)
50	10 (2.4%)	2 (3.4%)	0 (0.0%)	12 (2.5%)
60	8 (1.9%)	0 (0.0%)	1 (8.3%)	9 (1.9%)
70	2 (0.5%)	0 (0.0%)	0 (0.0%)	2 (0.4%)
80	5 (1.2%)	0 (0.0%)	0 (0.0%)	5 (1.0%)
90	10 (2.4%)	6 (10.3%)	0 (0.0%)	16 (3.3%)
100	19 (4.6%)	2 (3.4%)	1 (8.3%)	22 (4.6%)
N of Summed Total (%)	411 (85.4%)	58 (12.1%)	12 (2.5%)	481 (100%)

Supplementary Note 5: Use-wear analysis

We sampled 38 non-cortical blanks made of hornfels and 2 potential hammerstones from the Jojosi 5, 6 , and 7 locations (Table 45). The goal of the sampling strategy was to select complete flakes or blades with potentially active edges that are either rectilinear or convex in profile. Additionally, pieces displaying edge scarring or retouch were also chosen for in-depth microscopic observations. Methods for the analysis are described in the Main Text.

Supplementary Table 45. List of the artefacts subjected to microscopic investigation and related functional interpretation.

Progressive number	Find ID	Site	Layer/Feature	Find	Functional interpretation
1	718	Jojosi 5	Lens 1	Blank	No macro or microwear detected
2	721	Jojosi 5	Lens 1	Blank	No macro or microwear detected
3	728	Jojosi 5	Lens 1	Blank	No macro or microwear detected
4	743	Jojosi 5	Lens 1	Blank	No macro or microwear detected
5	769	Jojosi 5	Lens 1	Blank	No macro or microwear detected
6	771	Jojosi 5	Lens 1	Blank	No macro or microwear detected
7	780	Jojosi 5	Lens 1	Blank	No macro or microwear detected
8	790	Jojosi 5	Lens 1	Blank	No macro or microwear detected
9	802	Jojosi 5	Lens 1	Blank	Butchery
10	809	Jojosi 5	Lens 1	Blank	No macro or microwear detected
11	845	Jojosi 5	Lens 1	Blank	No macro or microwear detected
12	846	Jojosi 5	Lens 1	Blank	No macro or microwear detected
13	856	Jojosi 5	Lens 2	Blank	No macro or microwear detected
14	858	Jojosi 5	Lens 2	Blank	No macro or microwear detected
15	878	Jojosi 5	Lens 2	Blank	No macro or microwear detected
16	888	Jojosi 5	Lens 2	Blank	No macro or microwear detected
17	889	Jojosi 5	Lens 2	Blank	No macro or microwear detected
18	892	Jojosi 5	Lens 2	Blank	No macro or microwear detected
19	899	Jojosi 5	Lens 2	Blank	No macro or microwear detected
20	900	Jojosi 5	Lens 2	Hammerstone	No pitting or battering detected
21	910	Jojosi 5	Lens 2	Blank	No macro or microwear detected
22	924	Jojosi 5	Overburden	Hammerstone	No pitting or battering detected
23	929	Jojosi 5	Overburden	Blank	No macro or microwear detected
24	931	Jojosi 5	Lens 2	Blank	No macro or microwear detected
25	199	Jojosi 6	Lens 1	Blank	No macro or microwear detected
26	217	Jojosi 6	Lens 1	Blank	No macro or microwear detected
27	241	Jojosi 6	Lens 1	Blank	No macro or microwear detected
28	255	Jojosi 6	Lens 1	Blank	No macro or microwear detected
29	260	Jojosi 6	Lens 1	Blank	No macro or microwear detected
30	264	Jojosi 6	Lens 1	Blank	No macro or microwear detected
31	105	Jojosi 6	Lens 1	Blank	No macro or microwear detected
32	125	Jojosi 6	Lens 1	Blank	No macro or microwear detected
33	136	Jojosi 6	Lens 1	Blank	No macro or microwear detected
34	174	Jojosi 6	Lens 1	Blank	No macro or microwear detected
35	320	Jojosi 6	Lens 1	Blank	No macro or microwear detected
36	50	Jojosi 7	Lens 1	Blank	Non-diagnostic
37	340	Jojosi 7	Lens 1	Blank	Non-diagnostic
38	417	Jojosi 7	Lens 1	Blank	Non-diagnostic
39	434	Jojosi 7	Lens 1	Blank	Non-diagnostic
40	317	Jojosi 7	Lens 1	Blank	Non-diagnostic

The overall state of preservation of the sample was good, and we considered the sample suitable for a traceological investigation. All the blanks in Jojosi 5 and 6 had remarkably sharp and intact edges, with no evidence of mechanical changes such as fractures (whether modern or ancient), scars, or rounding of the edges or ridges due to taphonomic agents. This observation is consistent with the geological results and the spatial distribution of the lithic material, which suggests that the finds were buried rapidly in an environment of low-energy water action. However, all artefacts have a distinct white patina, characterised by small whitish spots that uniformly cover both surfaces. This observation indicates the beginning of a chemical change in the mineralogical composition of the hornfels that affected the Jojosi 5 and 6 localities (Figure 47a-b). This phenomenon is especially clear at Jojosi 7, where examining the rock surfaces of five artefacts revealed a distinct, porous, and hollow structure in the rock texture (Figure 47c-e).

The rock surfaces of the blanks in Jojosi 7 were too altered for any reliable reading of their wear. In addition, pieces recovered from this location showed evidence of sediment encrustations firmly attached to their surfaces. Even after being immersed in hot water, washed with demineralised water, and placed in an ultrasonic tank, large areas of the pieces remained covered with sediment, which prevented any visible traces from being detected (Figure 47f).

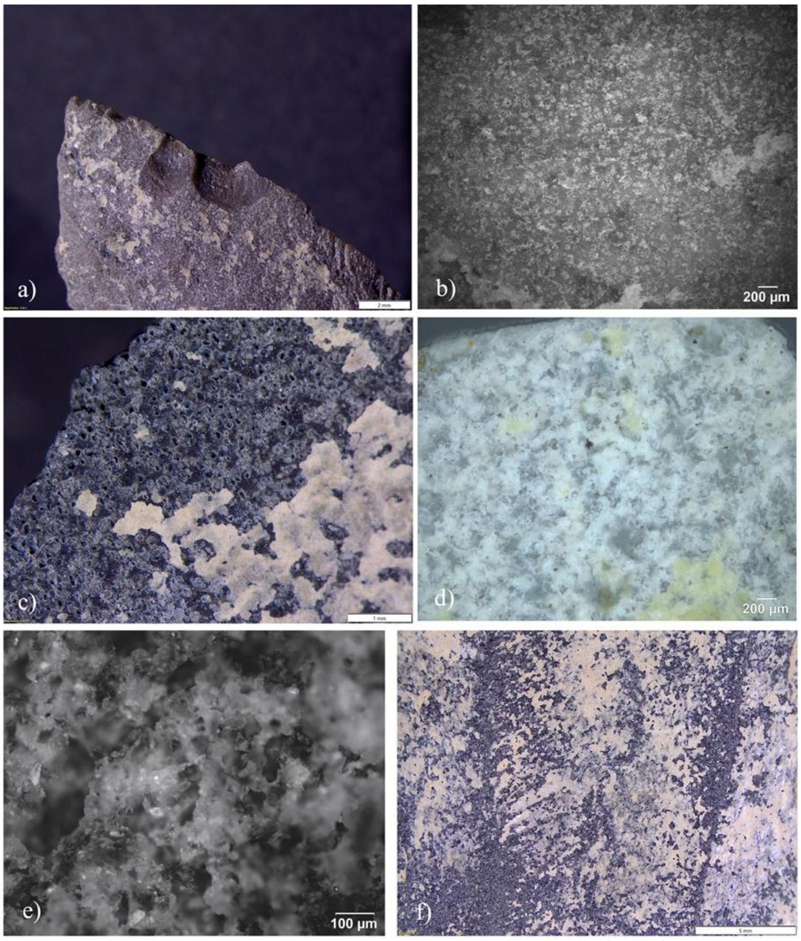

Supplementary Figure 47. Different degrees of alteration were observed on the sampled artefacts. a) ID 856, modern edge removals and light patina; notice the contrast between the fresh surface in the removals and the surrounding surface. b) ID 802, a light patina observed in dark field view. c) ID 50, a heavily altered surface with hollowed texture. d) ID 434, heavy white patination. e) ID 434, hollowed texture seen at higher magnification. f) ID 50, soil encrustations.

Out of 33 chipped stone artefacts from Jojosi 5 Lens 1 and 2, and Jojosi 6 that were examined at low and high power magnification, only one showed edge damage, rounding, and polishing that indicate use.

Blank n.802 from Jojosi 5 Lens 1 displayed moderate rounding and continuous aligned edge damage, mainly along the dorsal distal edge (Figure 48a-b). The edge damage consists of close-regular quadrangular scars with a feather termination. Additionally, isolated semicircular step-to-hinge terminating scarring is also observed (Figure 48b). A consistent polish, mainly along the ventral edge, has a rough texture and greasy topography (Figure 48c-d). When comparing the artefacts with the experimental reference collection that we created from Jojosi hornfels for this study, both macro and micro evidence suggest the artefact was most likely used for processing animal materials, such as skin, meat, and bone. The edge damage implies that the item was used on medium to hard materials through scraping.

The results from the traceological analysis reinforce and confirm the interpretation that these sites are raw material provision localities, exploited specifically for obtaining high-quality hornfels. Occasionally, modern humans might have used unretouched flakes or blades expediently, when needed, while formal tools were stored and carried elsewhere in the landscape. Lastly, the two items identified as hammerstones in Jojosi 5 did not exhibit any concentration of pitting and battering traces, which are typically observed on hammerstones used for lithic knapping.

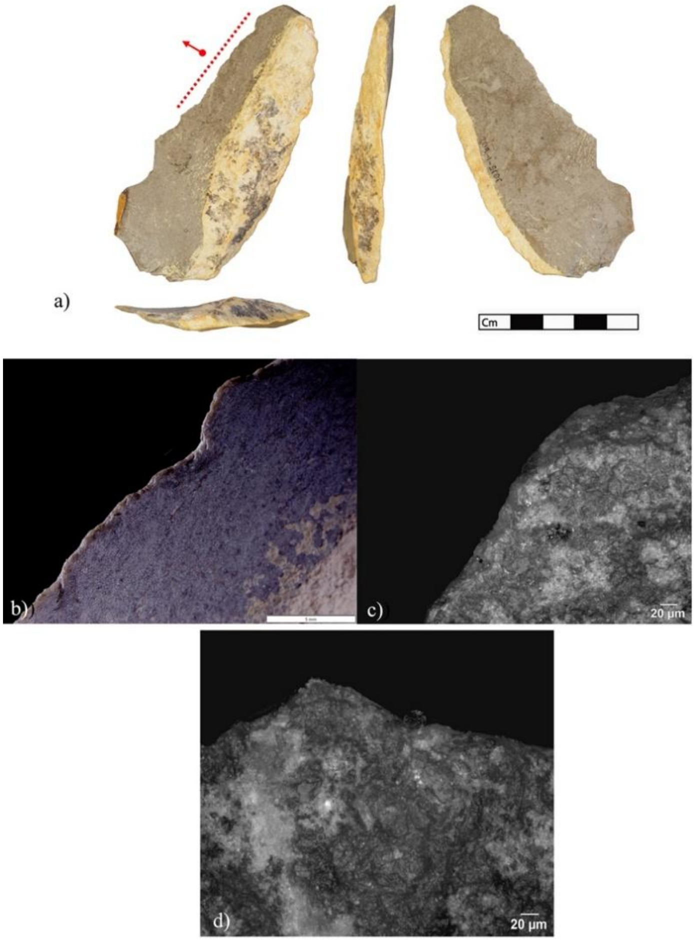

Supplementary Figure 48. Macro and microscopic use-related traces observed on item ID 802. a) Artefact ID 802 from Jojosi 5 lens 1; b) edge damage localised along the mesial and distal dorsal edge; c-d) polish observed along the ventral edge

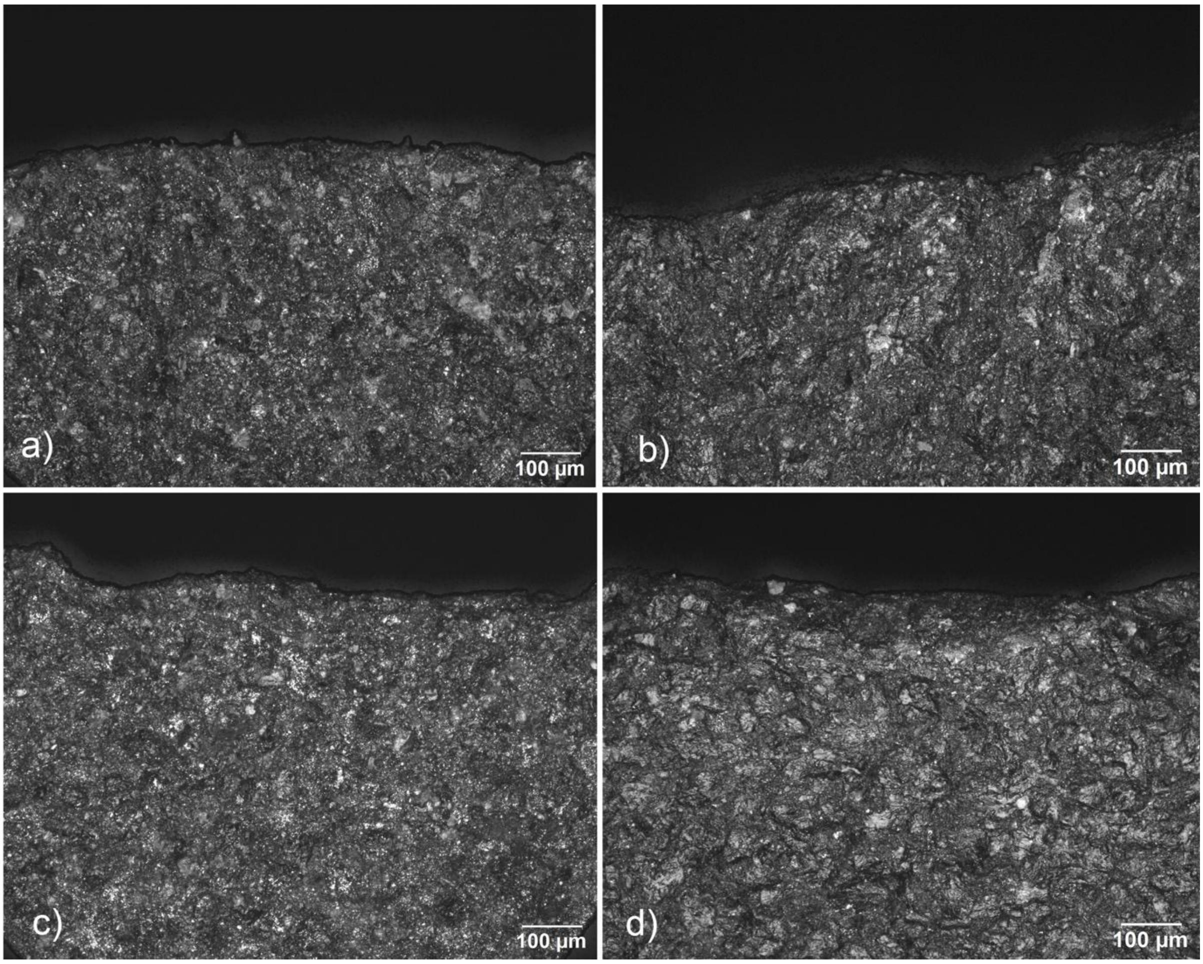

Supplementary Figure 49: Pre and post-use comparison on 2 experimental replicas on hornfels. a) pre-use flake HF5, dorsal face; b) post-use flake HF5 after cutting fresh wood, dorsal face; c) pre-use flake HF9, ventral face; d) post-use flake HF9 after cutting dry wood, ventral face.

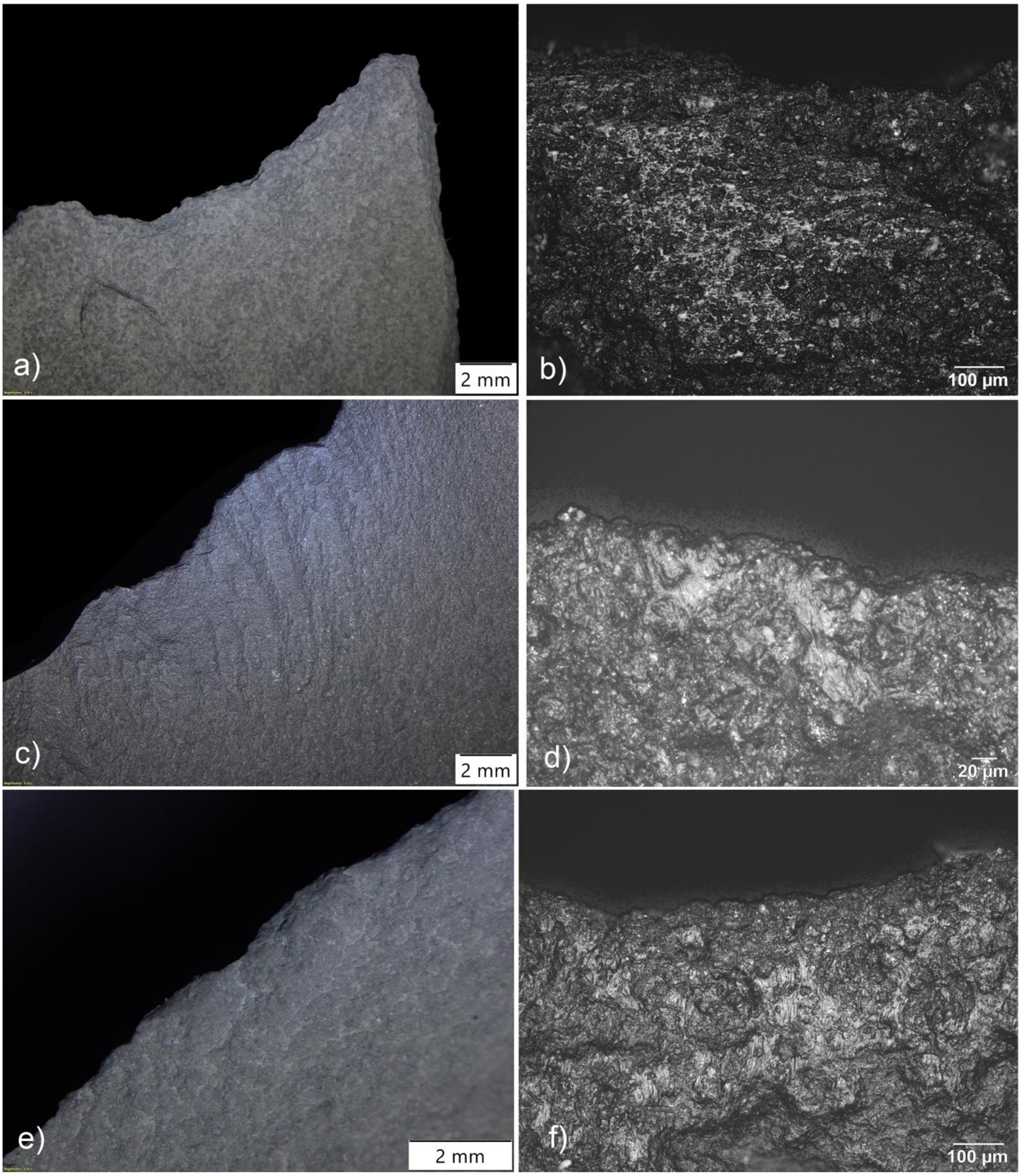

Supplementary Figure 50: Selection of edge damage and micro wear on the hornfels replicas. a) edge damage and rounding after debarking dry wood on the active edge of replica HF1; b) smooth and domed polish after sawing fresh wood on the active edge of replica HF8; c) edge damage and rounding after cleaning fresh bone from periosteum on the active edge of replica HF4; d) polish after cleaning fresh bone from periosteum on replica HF4; e) edge damage and rounding after cutting fresh bone on the active edge of replica HF27; f) polish after scraping fresh bone on the active edge of replica HF26.

Supplementary Table 46: List of the main experimental variables used in the reference collection.

ID	Edge angle	Contact material	Motion	Activity	Contact angle	Duration in minutes
HF1	~29°	Dry wood	Transversal unidirectional	Debarking	30° and 90°	60’
HF2	~15°	Fresh wood	Longitudinal unidirectional	Cutting	90°	60’
HF3	~22°	Fresh bone and fresh skin	Transversal uni and bidirectional	Removing periosteum	45° + 90°	60’
HF4	62°	Fresh bone and fresh skin	Longitudinal uni and bidirectional + Transversal uni and bidirectional	Cutting skin + removing periosteum and bone cleaning	45°	60’
HF5	~26°	Fresh wood	Longitudinal unidirectional	Cutting	90°	60’
HF6	19°	Fresh wood	Transversal unidirectional	Debarking	>45°	60’
HF7	~13°	Fresh bone and fresh skin	Longitudinal uni and bidirectional + Transversal uni and bidirectional	Longitudinal uni and bidirectional + Transversal uni and bidirectional	45° + 90°	60’
HF8	31°	Fresh wood	Longitudinal bidirectional	Sawing	90°	60’
HF9	~55°	Dry wood	Longitudinal unidirectional	Cutting	90°	60’
HF10	~28°	Fresh wood	Longitudinal unidirectional	Cutting	90°	60’
HF11	~53	Fresh bone and fresh skin	Longitudinal uni and bidirectional + Transversal uni and bidirectional	Longitudinal uni and bidirectional + Transversal uni and bidirectional	90° + 45°	60’
HF12	~43°	Fresh wood	Transversal unidirectional	Debarking	45°	60’
HF13	~74°	Fresh wood	Transversal bidirectional	Debarking	90°	60’
HF14		Fresh wood	Longitudinal unidirectional	Cutting	90°	60’
HF15	~22°	Fresh wood	Transversal bidirectional	Scraping	45°	60’
HF16	~27°	Fresh wood	Longitudinal bidirectional	Sawing	90°	60’
HF17	~40°	Fresh wood	Transversal unidirectional	Whittling	45°	60’
HF18	~21°	Hide	Longitudinal unidirectional	Cutting	90°	60’
HF19	~22°	Dray wood	Longitudinal bidirectional	Sawing	90°	60’
HF20	~35°	Dry wood	Longitudinal unidirectional	Cutting	90°	60’
HF21	41°	Dry wood	Transversal bidirectional	Scraping	45°	60’
HF22	~32°	Dry wood	Transversal unidirectional	Whittling	45°	60’
HF23	~25°	Fresh bone, meat and fat	Longitudinal unidirectional	Cutting	90°	60’
HF24	~18°	Fresh bone, meat and fat	Transversal uni + bidirectional	Bone cleaning	45°	60’
HF25	31°	Fresh bone, meat and fat	Longitudinal bidirectional	Sawing	90°	60’
HF26	~24°	Fresh bone	Transversal bidirectional	Scraping	45°	60’
HF27	20°	Fresh bone	Longitudinal unidirectional	Cutting	90°	60’

Supplementary Note 6: Zooarchaeological analysis of Jojosi 7

Lens 1 at locality Jojosi 7 is the only archaeological unit that has yielded identifiable faunal remains. The faunal sample comprises 26 piece-plotted remains, including seven tooth fragments and 19 bone fragments. Additional tooth fragments (enamel fragments, all characteristic of premolar/molar ungulate teeth; weight = 6 grams) and bone fragments (weight = 88 grams) were collected during sieving of the excavated sediment.

Specimen 394 (Figure 51) can confidently be assigned to the diastema of a bovid class size III (following the class size system proposed by[28]) mandible, consistent with the anatomical portion located between the incisors and the premolars. The other plotted bone fragments that can be assigned securely to a flat bone have very little or no spongy bone, which is also consistent with a mandible. The tooth fragments are almost exclusively premolar or molar enamel fragments of a large ungulate, while only two plotted tooth fragments could be enamel fragments of incisors, also from a large ungulate. Given their high fragmentation degree, these enamel fragments are conservatively assigned to a bovid class size II to IV. There is no indication that juvenile remains are present in the sample. Other, non-identifiable faunal remains are fragments of flat bones or fragments of non-identifiable bone. No bone fragment can be securely attributed to a long bone. Based on the cortical thickness of the bone fragments, there is no indication that the remains of smaller ungulates or smaller mammals are represented in the faunal assemblage.

We therefore suggest that all remains belong to a single element, namely the mandible of a large class III bovid. The faunal assemblage represents a minimum number of one individual. Large bovids from this class size that occur today in the area include grazers such as the black wildebeest *Connochaetes gnou*, the red hartebeest *Alcelaphus buselaphus*, and the waterbuck *Kobus ellipsiprymnus*[29].

The faunal material is extremely fragile, and the cortical surface is poorly preserved on most remains. The material is heavily fragmented, burnt, and it has a crumbly aspect. The majority of the remains are heavily burnt and have reached a black (carbonised) or white/greyish colour (calcined), or a combination of both (white/calcined on the outer, cortical surface and black/carbonised on the inner, medullar surface)[30]. This suggests that the mandible was exposed to high temperatures and/or to the heat of a fire for a prolonged period of time.

Specimen 394 bears butchery marks (Figure 51). It is not possible to attribute a definitive side to this fragment, given its fragmentary nature, but we propose tentatively that it is the fragment of a left mandible, in which case the cut marks are located on the buccal portion and can be interpreted as the result of skinning. One tiny bone fragment from the sieving fraction also has butchery marks on its surface (Figure 51).

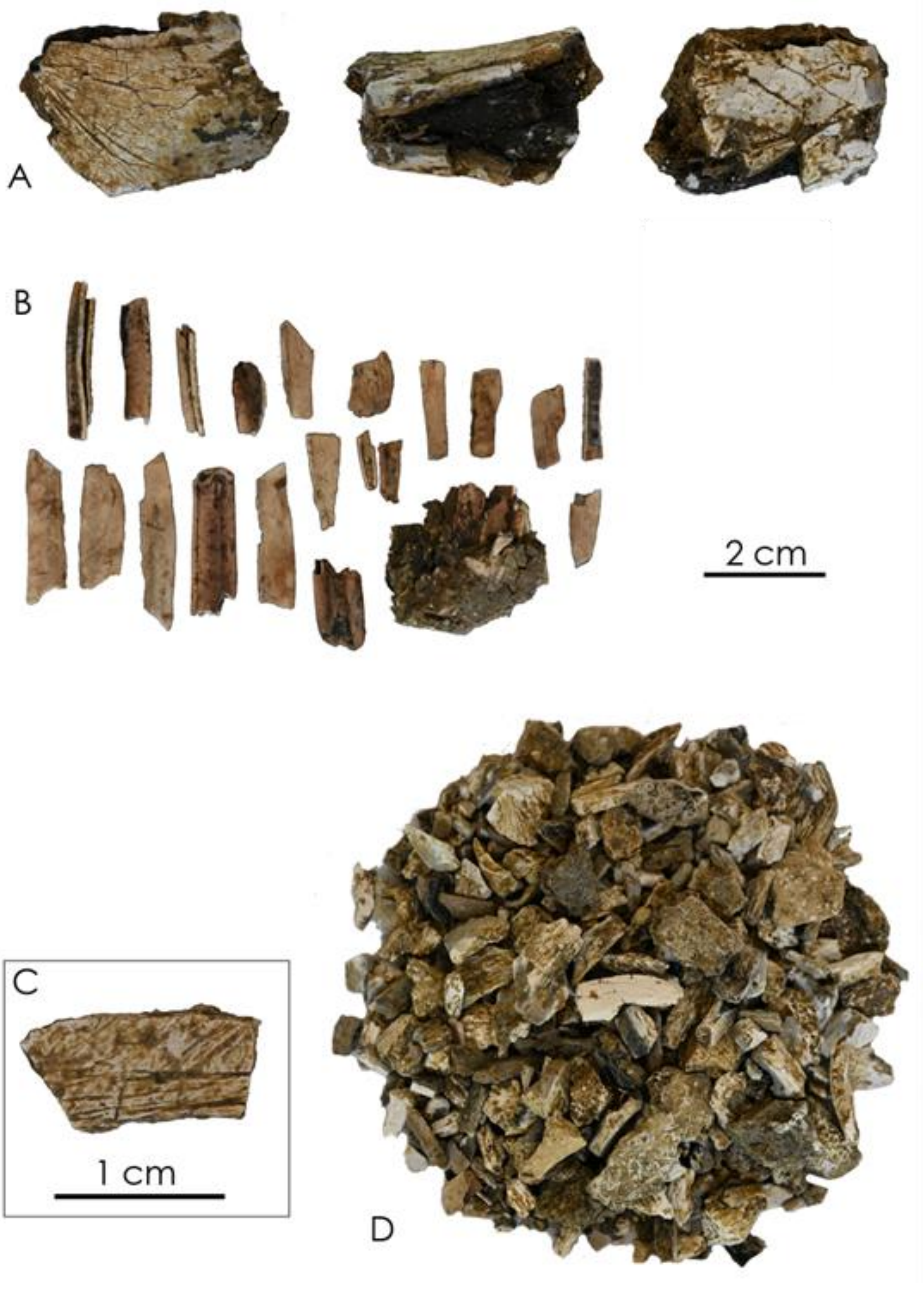

Supplementary Figure 51. Selected faunal remains from Jojosi 7, Lens 1. A) Specimen 394, fragment of the mandibular diastema of a large ungulate. From left to right: buccal view (with possible butchery marks), occlusal view, lingual view. B) Specimen 192, fragments of a single shattered large bovid (class size III) molar or premolar. C) Non-identifiable bone fragment from the sieving fraction, with possible butchery marks. D) Tiny bone fragments retrieved during sieving; note the abundance of calcined (white, white, and grey) fragments.

Supplementary References

1) Botha, G.A., Wintle, A.G. and Vogel, J.C. 1994. Episodic Late Quaternary palaeogully erosion in northern Kwazulu-Natal, South Africa. Catena, 23, p. 327-340. https://doi.org/10.1016/0341-8162(94)90076-0
2) Botha, G.A. 1996. The geology and palaeopedology of late Quaternary colluvial sediments in northern KwaZulu-Natal, South Africa. Memoir of the Geological Survey of South Africa, 83, Council for Geoscience, Pretoria. 165pp.
3) Möller, G. H. D. et al. Revisited and revalorised: Technological and refitting studies at the Middle Stone Age open-air knapping site Jojosi 1 (KwaZulu-Natal, South Africa). *J. Paleolith. Archaeol.* **8**, 5 (2025).
4) Aitken, M.J., 1985. Thermoluminescence Dating. Academic Press, London, United Kingdom.
5) Duller, G.A.T., 2008. Single-grain optical dating of Quaternary sediments: why aliquot size matters in luminescence dating, Boreas, 37, 589-612, doi: 10.1111/j.1502-3885.2008.00051.x
6) Bøtter-Jensen, L., Andersen, C.E., Duller, G.A.T., Murray, A.S., 2003. Developments in radiation, stimulation and observation facilities in luminescence measurements, Radiat. Meas., 37, 535-541, doi:10.1016/S1350-4487(03)00020-9
7) Huntley, D.J., Godfrey-Smith, D.I., Haskell, E.H., 1991. Light-induced emission spectra from some quartz and feldspars, Nucl. Tracks Radiat. Meas., 18, 127-131
8) Murray, A.S., Wintle, A.G., 2000. Luminescence dating of quartz using an improved single-aliquot regenerative-dose protocol. Radiat. Meas. 32, 57-73, doi: 10.1016/S1350-4487(99)00253-X
9) Thomsen, K.J., Murray, A.S., Jain, M., Bøtter-Jensen, L., 2008. Laboratory fading rates of various luminescence signals from feldspar-rich sediment extracts, Radiation Measurements 43, 1474-1486, doi: 10.1016/j.radmeas.2008.06.002
10) Riedesel, S., Guérin, G., Thomsen, K., Sontag-González, M., Blessing, M., Botha, G., Möller, G., Peffeköver, A., Sommer, C., Zander, A., Will, M., 2025. A direct comparison of single grain and multiple grain aliquot measurements of feldspars from donga deposits in KwaZulu-Natal, South Africa. Geochronology 7, 59-81. Doi: 10.5194/gchron-7-59-2025
11) Auclair, M., Lamothe, M., Huot, S., 2003. Measurement of anomalous fading for feldspar IRSL using SAR, Radiat. Meas., 37, 487-492, doi:10.1016/S1350-4487(03)00018-0.
12) Heydari, M., Guérin, G., 2018. OSL signal saturation and dose rate variability: Investigating the behaviour of different statistical models, Radiat. Meas., 120, 96-103, doi: 10.1016/j.radmeas.2018.05.005
13) Chapot, M.S., Duller, G.A.T., Barham, L.S., 2022. Challenges of dating quartz OSL samples with saturated grains: Lessons from single-grain analyses of low dose-rate samples from Victoria Falls, Zambia, Quat. Geochronol., 72, 101344, doi: 10.1016/j.quageo.2022.101344
14) Arce-Chamorro, C., Guérin, G., 2024. Comparison of De values from Late Pleistocene alluvial deposits on the coast of Galicia (NW Spain) using BayLum or Analyst-based procedures, Quat. Geochronol., 82, 101540, doi:10.1016/j.quageo.2024.101540
15) Philippe, A., Guerin, G., Kreutzer, S., 2019. BayLum – An R package for Bayesian analysis of OSL ages: An introduction. Quat. Geochronol. 49, 16-24, doi: 10.1016/j.quageo.2018.05.009
16) Krapp, M., Beyer, R. M., Edmundson, S. L., Valdes, P. J., & Manica, A. (2021). A statistics-based reconstruction of high-resolution global terrestrial climate for the last 800,000 years. Scientific Data, 8(1), 228.
17) Bøtter-Jensen, L., Mejdahl, V., 1985. Determination of potassium in feldspars by beta counting using a GM multicounter system, Nucl. Tracks, 10, 663-666, doi:10.1016/0735-245X(85)90073-0
18) Govindaraju, K., 1995. Working values with confidence limits for twenty-six CRPG, ANRT and IWG-GIT geostandards, Geostand. Newsl., 19 (special), 1-32.
19) Durcan, J.A., King, G.E., Duller, G.A.T., 2015. DRAC: Dose rate and age calculator for trapped charge dating, Quat. Geochronol., 28, 54-61, doi: 10.1016/j.quageo.2015.03.012
20) Riedesel, S., Autzen, M., Burow, C., 2023. scale_GammaDose(): Calculate the gamma dose deposited within a sample taking layer-to-layer variations in radioactivity into account (according to Aitken, 1985). Function version 0.1.2. In: Kreutzer, S., Burow, C., Dietze, M., Fuchs, M.C., Schmidt, C., Fischer, M., Friedrich, J., Mercier, N., Philippe, A., Riedesel, S., Autzen, M., Mittelstrass, D., Gray, H.J., and Galharret, J.: Luminescence: Comprehensive Luminescence Dating Data Analysis. R package version 0.9.22, https://CRAN.R-project.org/package=Luminescence
21) Guérin, G., Mercier, N., Adamiec, G., 2011. Dose-rate conversion factors: update, Ancient TL, 29, 5-8.
22) Bell, W. T. (1980). Alpha dose attenuation in quartz grains for thermoluminescence dating. Ancient Tl, 4, 4-8.
23) Guérin, G., Mercier, N., Nathan, R., Adamiec, G., & Lefrais, Y. (2012). On the use of the infinite matrix assumption and associated concepts: a critical review. Radiation Measurements, *47*(9), 778-785.
24) Balescu, S., Lamothe, M., 1993. Thermoluminescence dating of the Holsteinian marine formation of Herzeele, northern France. Journal of Quaternary Science 8, 117-124. Doi: 10.1002/jqs.3390080204
25) Prescott, J.R., Hutton, J.T., 1994. Cosmic ray contributions to dose rates for luminescence and ESR dating: Large depths and long-term time variations, Radiat. Meas., 23, 497-500, doi: 10.1016/1350-4487(94)90086-8
26) Will, M. et al. (2024) The Jojosi Dongas: an interdisciplinary project to study the evolution of human behaviour and landscapes in open-air contexts. South. Afr. Field Archaeol. 19, 1–28.
27) Sanderson, D. J., & Peacock, D. C. (2020). Making rose diagrams fit-for-purpose. Earth-Science Reviews, 201, 103055.
28) Brain, C.K., 1974. Some suggested procedures in the analysis of bone accumulations from southern African Quaternary sites. Annals of the Transvaal Museum 29 (1), 1–8.
29) Skinner, J.D. & Chimimba, C.T. 2005. *The Mammals of the Southern African Sub-Region*. Cambridge University Press.
30) Stiner, M. C., Kuhn, S. L., Weiner, S., Bar-Yosef, O., 1995. Differential burning, recrystallization, and fragmentation of archaeological bone. Journal of Archaeological Science 22, 223–237.
