# Supplementary material for: Specialised and persistent raw material procurement by humans in the Middle Pleistocene
Source: Nat Commun. 2026 Apr 7;17:2702. doi: 10.1038/s41467-026-70783-8 (PMC13057256; doi:10.1038/s41467-026-70783-8)
Supplement: Supplementary file 2 — Reporting Summary [file 41467_2026_70783_MOESM2_ESM.pdf]

nature portfolio

Corresponding author(s): Dr. Manuel Will

Last updated by author(s): Mar 2, 2026

Reporting Summary

Nature Portfolio wishes to improve the reproducibility of the work that we publish. This form provides structure for consistency and transparency in reporting. For further information on Nature Portfolio policies, see our Editorial Policies and the Editorial Policy Checklist.

Statistics

For all statistical analyses, confirm that the following items are present in the figure legend, table legend, main text, or Methods section.

n/a	Confirmed	
☐	☒	The exact sample size (*n*) for each experimental group/condition, given as a discrete number and unit of measurement
☒	☐	A statement on whether measurements were taken from distinct samples or whether the same sample was measured repeatedly
☒	☐	The statistical test(s) used AND whether they are one- or two-sided<br>*Only common tests should be described solely by name; describe more complex techniques in the Methods section.*
☒	☐	A description of all covariates tested
☒	☐	A description of any assumptions or corrections, such as tests of normality and adjustment for multiple comparisons
☐	☒	A full description of the statistical parameters including central tendency (e.g. means) or other basic estimates (e.g. regression coefficient) AND variation (e.g. standard deviation) or associated estimates of uncertainty (e.g. confidence intervals)
☒	☐	For null hypothesis testing, the test statistic (e.g. *F*, *t*, *r*) with confidence intervals, effect sizes, degrees of freedom and *P* value noted<br>*Give P values as exact values whenever suitable.*
☒	☐	For Bayesian analysis, information on the choice of priors and Markov chain Monte Carlo settings
☒	☐	For hierarchical and complex designs, identification of the appropriate level for tests and full reporting of outcomes
☒	☐	Estimates of effect sizes (e.g. Cohen's *d*, Pearson's *r*), indicating how they were calculated

Our web collection on statistics for biologists contains articles on many of the points above.

Software and code

Policy information about availability of computer code

Data collection	Microsoft Excel, Microsoft Access
Data analysis	Microsoft Excel, R (version 4.4.1), Agisoft Metashape (versions 2.2.1 and 2.1.4), QGIS (version 3.34)

For manuscripts utilizing custom algorithms or software that are central to the research but not yet described in published literature, software must be made available to editors and reviewers. We strongly encourage code deposition in a community repository (e.g. GitHub). See the Nature Portfolio guidelines for submitting code & software for further information.

Data

Policy information about availability of data

All manuscripts must include a data availability statement. This statement should provide the following information, where applicable:

- Accession codes, unique identifiers, or web links for publicly available datasets
- A description of any restrictions on data availability
- For clinical datasets or third party data, please ensure that the statement adheres to our policy

The data generated in this study are provided in the main article and the Supplementary Information. Source data are provided as a Source Data file. All archaeological material of this study is permanently stored at the KwaZulu-Natal Museum in Pietermaritzburg, 237 Jabu Ndlovu St., South Africa, with access via the Principal Curator, Dr. Geoffrey Blundell (gblundell@nmsa.org.za).

Research involving human participants, their data, or biological material

Policy information about studies with human participants or human data. See also policy information about sex, gender (identity/presentation), and sexual orientation and race, ethnicity and racism.

Reporting on sex and gender	n/a
Reporting on race, ethnicity, or other socially relevant groupings	n/a
Population characteristics	n/a
Recruitment	n/a
Ethics oversight	n/a

Note that full information on the approval of the study protocol must also be provided in the manuscript.

Field-specific reporting

Please select the one below that is the best fit for your research. If you are not sure, read the appropriate sections before making your selection.

☐ Life sciences ☐ Behavioural & social sciences ☒ Ecological, evolutionary & environmental sciences

For a reference copy of the document with all sections, see nature.com/documents/nr-reporting-summary-flat.pdf

Ecological, evolutionary & environmental sciences study design

All studies must disclose on these points even when the disclosure is negative.

Study description	We performed archaeological and geological fieldwork and studied the excavated archaeological material at the site of Jojosi (South Africa) using geomorphological and geological methods, luminescence dating as well as technological, use wear, and refitting analyses of the recovered stone tools. The main objectives of the study were to reconstruct the site formation processes of the artefact assemblage and associated deposits, understand the behaviors that took place at the site in the context of the geological surroundings (e.g., a primary raw material outcrop), and provide absolute ages for these human occupations.
Research sample	The research sample consists of a total of four excavated sites (Jojosi 1, 5, 6 and 7), their deposits and archaeological material. The archaeological material encompasses a total of 20,853 stone artefacts in situ (n=1443 >20 mm; n=19410 <20 mm) as well as n=26 fragmentary animal bones.
Sampling strategy	We analyzed the sedimentary contexts around the three modernly-excavated sites (Jojosi 5,6 and 7) and studied the archaeological material of all four Jojosi localities. A total of n=1443 stone artefacts (with a size cut-off of 20 mm) were analyzed individually for this study. A total of n=40 artefacts were selected for further use-wear analysis. All recovered artifacts <20 mm were counted and classified by raw material.
Data collection	Geomorphological and sedimentary analyses were performed by C. Sommer and G. Botha for archaeological sites as well as selected geological sequences. Relevant data in the field was documented in notebooks and laptops. Lithic analysis by combined attribute analysis, chaine operatoire and refitting was performed by G. Möller, supervised by M. Will. Data were entered in a Microsoft Access database with the help of a scale, caliper and goniometer. Use wear analysis was conducted by F. Venditti. S. Riedesel undertook the luminescence dating of the sites. Zooarchaeological analyses of Jojosi 7 was performed by A. Val. All authors helped in data collection during fieldwork. Detailed methods of data collection are described in the Methods section of the article and the Supplementary Information.
Timing and spatial scale	Geological and archaeological fieldwork for this study took place in 1991 (directed by A. Mazel, Jojosi 1, legacy collections of this study), as well as in 2022, 2023 and 2024 (directed by M. Will, main focus of this study). Each of these fieldwork campaigns had a duration of between 2-3 weeks. Geological and archaeological surveys took place over the entire dongas of the Jojosi areas (~2 km^2) with excavations being constricted to small-scale excavations at the four Jojosi locales with a total of approximately 15 m^2.
Data exclusions	We did not exclude any data for analysis.
Reproducibility	We provide all relevant method details and data to reproduce our results in the main text and Supplementary Information.
Randomization	n/a
Blinding	n/a

Did the study involve field work? ☒ Yes ☐ No

Field work, collection and transport

Field conditions	Fieldwork took place in the Jojosi dongas, located near the town of Nquthu (KwaZulu-Natal). Being an open-air site, field conditions depended entirely on the current weather. The weather was mostly dry, hot and sunny, but during some days rain and wind complicated excavations and sieving. Overall, as we adapted our field and recovery methods to the weater, no negative effects were observed on the recovered archaeological material.
Location	The Jojosi dongas have the following coordinates: 28°08'29.9"S 30°39'00.1"E; approximately 140 km from the coast at an elevation of ~1200 m
Access & import/export	For the fieldwork and export (stone tools) of archaeological material all relevant permits were obtained prior to any work. For the archaeological excavations and collection of archaeological material the permits were issued by the local heritage agency AMAFA (PermitID: 3848 REF: SAH22/18276; 3850 REF: SAH22/18276) to M. Will, valid from 05/12/2022 to 05/12/2025. The permit for analysis and temporary export of the Jojosi 5 & 6 stone tools was issued by AMAFA (PermitID: 3989 REF: SAH23/21517 & 23/087) and SAHRA (CaseID: 22070; PermitID: 3968) to M. Will. All exported artefacts were returned to the KwaZulu-Natal Museum in Pietermaritzburg in January 2025.
Disturbance	Archaeological excavations always cause the destruction of the sites being dug. Due to this reason, we took extra care to excavate the site with modern standards to recover all remains and spatial contexts, including 3D point plotting and sieving of all sediments.

Reporting for specific materials, systems and methods

We require information from authors about some types of materials, experimental systems and methods used in many studies. Here, indicate whether each material, system or method listed is relevant to your study. If you are not sure if a list item applies to your research, read the appropriate section before selecting a response.

Materials & experimental systems

n/a	Involved in the study	
☒	☐	Antibodies
☒	☐	Eukaryotic cell lines
☐	☒	Palaeontology and archaeology
☒	☐	Animals and other organisms
☒	☐	Clinical data
☒	☐	Dual use research of concern
☒	☐	Plants

Methods

n/a	Involved in the study	
☒	☐	ChIP-seq
☒	☐	Flow cytometry
☒	☐	MRI-based neuroimaging

Palaeontology and Archaeology

Specimen provenance	All archaeological material was recovered within the Jojosi dongas (sites Jojosi 1, 5, 6 and 7). For the fieldwork and export (stone tools) of archaeological material all relevant permits were obtained prior to this work. For the archaeological excavations and collection of archaeological material the permits were issued by the local heritage agency AMAFA (PermitID: 3848 REF: SAH22/18276; 3850 REF: SAH22/18276) to M. Will, valid from 05/12/2022 to 05/12/2025. The permit for analysis and temporary export of the Jojosi 5 & 6 stone tools was issued by AMAFA (PermitID: 3989 REF: SAH23/21517 & 23/087) and SAHRA (CaseID: 22070; PermitID: 3968) to M. Will.
Specimen deposition	All archaeological material is permanently stored at the KwaZulu-Natal Museum in Pietermaritzburg, 237 Jabu Ndlovu St., South Africa.
Dating methods	Fourteen luminescence samples were collected in total by hammering opaque stainless-steel tubes into cleaned outcrop surfaces or by carving blocks from exposed outcrops. For dose rate determination, additional samples were taken from the sediment surrounding the luminescence samples. High-resolution gamma spectrometry and beta counting were used to determine the sediment dose rate delivered to the samples, and for internal dose rate determination, respectively. Variability in U, Th, and K concentrations in layers influencing some of the luminescence samples necessitated scaling the gamma dose rate. Samples for dose determination were prepared under subdued red-light conditions in the Cologne Luminescence Laboratory (CLL; University of Cologne) where also all other steps were performed. The Dose Rate and Age Calculator (DRAC) was used to calculate environmental dose rates for each luminescence sample.<br><br>For luminescence dating, sand-sized feldspar grains (200-250 µm) were retrieved from the samples under red light conditions using chemical treatments, sieving, and heavy liquid density separation. Luminescence measurements of small multi-grain aliquots (1 mm diameter) were performed using Risø luminescence instruments. Luminescence measurements were performed following a post-infrared infrared stimulated luminescence (post-IR IRSL225) protocol.<br><br>Further details are given in the supplementary material.

☒ Tick this box to confirm that the raw and calibrated dates are available in the paper or in Supplementary Information.

Ethics oversight

No ethical approvance and guidance was required but all legal requirements were followed (i.e. obtaining relevant permits for excavation and colleciton). Morena Molefe of Batlokoa Ba Molefe and the Tribal Council granted us permission to do the research in their traditional authority area on which the Jojosi sites are located. We are in constant exchange with the Tribal Council on the results of our work.

Note that full information on the approval of the study protocol must also be provided in the manuscript.

Plants

Seed stocks

Report on the source of all seed stocks or other plant material used. If applicable, state the seed stock centre and catalogue number. If plant specimens were collected from the field, describe the collection location, date and sampling procedures.

Novel plant genotypes

Describe the methods by which all novel plant genotypes were produced. This includes those generated by transgenic approaches, gene editing, chemical/radiation-based mutagenesis and hybridization. For transgenic lines, describe the transformation method, the number of independent lines analyzed and the generation upon which experiments were performed. For gene-edited lines, describe the editor used, the endogenous sequence targeted for editing, the targeting guide RNA sequence (if applicable) and how the editor was applied.

Authentication

Describe any authentication procedures for each seed stock used or novel genotype generated. Describe any experiments used to assess the effect of a mutation and, where applicable, how potential secondary effects (e.g. second site T-DNA insertions, mosiacism, off-target gene editing) were examined.
